# Author Correction: Healthy forests safeguard traditional wild meat food systems in Amazonia

**DOI:** 10.1038/s41586-026-10332-x

**Published:** 2026-03-03

**Authors:** André Pinassi Antunes, Pedro de Araujo Lima Constantino, Julia E. Fa, Daniel P. Munari, Thais Q. Morcatty, Michelle C. M. Jacob, Bruce W. Nelson, Mariana Franco Cassino, Elildo A. R. Carvalho, Amy Ickowitz, Lauren Coad, Richard E. Bodmer, Pedro Mayor, Cecile Richard-Hansen, João Valsecchi, João V. Campos-Silva, Juarez C. B. Pezzuti, Miguel Aparício, Eduardo M. von Muhlen, Marcela Alvares Oliveira, Milton J. de Paula, Natalia C. Pimenta, Marina A. R. de Mattos Vieira, Marcelo A. Santos Junior, André V. Nunes, Jean P. Boubli, Luan M. G. Suruí, Eneias C. S. Paumari, Abimael V. C. Paumari, José Lino V. S. Paumari, Germano C. Paumari, Ana Paula L. R. Katukina, Dzoodzo Baniwa, Valencio S. M. Baniwa, Walter S. L. Baniwa, Abel O. F. Baniwa, Armindo B. Baniwa, Isaías J. S. Baniwa, Yaukuma Waura, Jairo Silvestre Apurinã, Valdir S. S. Apurinã, Josiane O. G. Tikuna, Elias P. A. L. Tikuna, José L. Kaxinauá, Kussugi B. Kuikuro, Jorge T. Penaforth Kaixana, George H. Rebelo, Dione Torquato, Vanessa S. F. Apurinã, Miguel Antúnez, Pedro E. Perez-Peña, Tula G. Fang, Pablo E. Puertas, Rolando M. Aquino, Louise Maranhão, Guillaume Longin, Cíntia K. M. Lopes, Hani R. El Bizri

**Affiliations:** 1 Rede de Pesquisa em Conservação, Uso e Manejo da Fauna da Amazônia (RedeFauna), Manaus, Brazil; 2 Instituto Nacional de Pesquisas da Amazônia (INPA), Manaus, Brazil; 3 Comunidad de Manejo de Fauna Silvestre en América, Iquitos, Peru; 4 Instituto Juruá, Manaus, Brazil; 5 Centro Nacional de Pesquisa e Conservação de Mamíferos Carnívoros, Instituto Chico Mendes de Conservação da Biodiversidade, Atibaia, Brazil; 6 Department of Natural Sciences, Faculty of Science and Engineering, Manchester Metropolitan University, Manchester, UK; 7 Center for International Forestry Research, World Agroforestry (CIFOR-ICRAF), Bogor, Indonesia; 8 Natural Sciences and Environment Hub, University of Gibraltar, Gibraltar, Gibraltar; 9 Wildlife Conservation Society Brazil Program, Manaus, Brazil; 10 Department of Geography, University College London, London, UK; 11 Instituto de Desenvolvimento Sustentável Mamirauá, Tefé, Brazil; 12 Laboratory of Biodiversity and Nutrition, Federal University of Rio Grande do Norte, Natal, Brazil; 13 Núcleo de Estudos da Amazônia Indígena (NEAI), Universidade Federal do Amazonas (UFAM), Manaus, Brazil; 14 ICCS, University of Oxford, Oxford, UK; 15 Museo de Culturas Indígenas Amazónicas, Fundamazonia, Iquitos, Peru; 16 Durrell Institute of Conservation and Ecology (DICE), University of Kent, Canterbury, Kent UK; 17 Departament de Sanitat i Anatomia Animals, Universitat Autonoma de Barcelona, Barcelona, Spain; 18 OFB/DRAS/SEE, UMR Ecofog, Campus Agronomique Kourou, Kourou, French Guiana; 19 Núcleo de Altos Estudos Amazônicos, Universidade Federal do Pará, Belém, Brazil; 20 Departamento de Vertebrados, Museu Nacional do Rio de Janeiro, Rio de Janeiro, Brazil; 21 Centro Universitário Aparício Carvalho, Porto Velho, Brazil; 22 Instituto Federal de Educação, Ciência e Tecnologia do Pará, Altamira, Brazil; 23 Instituto Socioambiental, Manaus, Brazil; 24 Universidade Estadual de Campinas, Campinas, Brazil; 25 Fundação Vitória Amazônica (FVA), Manaus, Brazil; 26 Instituto Nacional de Pesquisas Espaciais (INPE), São José dos Campos, Brazil; 27 Instituto de Pesquisas Ecológicas (IPE), Campo Grande, Brazil; 28 School of Science Engineering and the Environment, University of Salford, Salford, UK; 29 Paiter Suruí Indigenous People, Cacoal, Brazil; 30 Paumari Indigenous People, Tapauá, Brazil; 31 Katukina Indigenous People, Tapauá, Brazil; 32 Baniwa Indigenous People, São Gabriel da Cachoeira, Brazil; 33 Waura Indigenous People, Gaúcha do Norte, Brazil; 34 Apurinã Indigenous People, Lábrea, Brazil; 35 Tikuna Indigenous People, São Paulo de Olivença, Brazil; 36 Huni Kuin Indigenous People, Tarauacá, Brazil; 37 Kuikuro Indigenous People, Gaúcha do Norte, Brazil; 38 Fundação Nacional do Povos Indígenas, São Paulo de Olivença, Brazil; 39 Conselho Nacional das Populações Extrativistas (CNS), Brasília, Brazil; 40 Coordenação das Organizações Indígenas da Amazônia Brasileira (COIAB), Manaus, Brazil; 41 Instituto del Bien Común, Paisaje Putumayo Amazonas, Loreto, Perú; 42 Instituto de Investigaciones de la Amazonía Peruana (IIAP), Loreto, Perú; 43 Asociación Centro de Innovación Científica Amazónica (CINCIA), Loreto, Perú; 44 Instituto de Ciencias Biológicas, Facultad de Ciencias Biológicas, Universidad Nacional Mayor de San Marcos, San Marcos, Perú; 45 Parc Amazonien de Guyane, Remire-Montjoly, French Guiana; 46 Programa de Pós-Graduação em Zoologia, Universidade Estadual do Pará, Belém, Brazil; 47 Museu Paraense Emílio Goeldi, Belém, Brazil

**Keywords:** Sustainability, Biodiversity

Correction to: *Nature* 10.1038/s41586-025-09743-z Published online 26 November 2025

In the version of this article originally published, due to a figure preparation mistake, the map shown as Fig. 1e was an inadvertent duplicate of Fig. 1d. The error is in presentation only, and the caption and data remain correct. The figure is now updated in the HTML and PDF versions of the article.

