# Supplementary material for: Healthy forests safeguard traditional wild meat food systems in Amazonia
Source: Nature. 2025 Nov 26;648(8094):625–33. doi: 10.1038/s41586-025-09743-z (PMC12711560; doi:10.1038/s41586-025-09743-z)
Supplement: Supplementary file 1 — Supplementary Methods 1–7, Supplementary Data 1–5 and Supplementary Tables 1–11 [file 41586_2025_9743_MOESM1_ESM.pdf]

nature portfolio

https://doi.org/10.1038/s41586-025-09743-z

Supplementary information

Healthy forests safeguard traditional wild meat food systems in Amazonia

In the format provided by the
authors and unedited

Supplementary information guide

Table of Contents

Supplementary Method 1. Environmental and anthropogenic spatial variables used for modeling Harvest Productivity (HP) and Taxon-Specific Offtake Proportion (TSOP): Enhanced Vegetation Index (EVI) (A), Annual Gross Primary Productivity (GPP) (B), Annual Net Primary Productivity (NPP) (C), Soil Fertility (D), Proportion of flooded areas (E), Elevation (F), Height above the nearest drainage (HAND) (G), Historical distribution of Indigenous family languages (H), Current distribution of Indigenous and non-Indigenous peoples (I), Current distribution of family languages (J), Proportion of habitat loss (K), and Urban-rural catchment areas (URCA) (L). 1

Supplementary Method 2. Historical distribution of Indigenous family languages in Amazonia. Raster produced based on maps provided in Loukotka (1967)[71] and Eriksen (2011)[72]. 2

Supplementary Method 3. Current distribution of family languages. A categorical digital database built from the Amazon Network of Georeferenced Socio-Environmental Information (RAISG) database of the distribution of the Indigenous Lands[73], classified into their respective Indigenous family languages. Regions outside the Indigenous Lands are tentatively classified as Latin/German languages. 3

Supplementary Data 1. Variable importance output of the Overall Individual Animals Harvest Productivity (HP) Random Forest model. For the purpose to show the variable importance for HP, we ran just one model with all the data. 4

Supplementary Data 2. Distribution of tetrapod vertebrate species (mammals, birds, reptiles and amphibians) richness in Amazonia. Raster representing the number of tetrapod species per pixel built using information from the IUCN spatial database (IUCN 2024)[20]. 5

Supplementary Table 1. List of species hunted in Amazonia recorded in this study. 6

Supplementary Table 2. Estimated number of individual animals hunted, animal biomass extracted (kg), body mass (kg), density (ind/km2), geographic distribution area in Amazonia (km2) and number of animals hunted/km2 per taxon per year in Amazonia....21

Supplementary Data 3. Estimated body mass for the 174 hunted taxa recorded in this study....27

Supplementary Table 3. List of the 63 species pertaining to the 20 key dominant hunted taxa in Amazonia classified into one of nine IUCN Red List of Threatened Species Categories[20]. Extinct (EX), Extinct in the Wild (EW), Critically Endangered (CR), Endangered (EN), Vulnerable (VU), Near Threatened (NT), Least Concern (LC), Data Deficient (DD) and Not Evaluated. 28

Supplementary Table 4. Estimated number of animals hunted (Individual Animals Offtake) for the 30 most hunted taxa and its proportion in relation to the total offtake in each Amazonian region. 30

Supplementary Table 5. Estimated proportion of the number of animals hunted (Individual Animals Offtake) per animal group in relation to the total offtake in each Amazonian region....36

Supplementary Table 6. Estimated number of animals hunted (Individuals Animals Offtake) for the 30 most hunted taxa and their proportion in relation to the total offtake in upland terra firme forests (regions > 50 % of upland terra firme forests) and flooded forests (regions > 50 % of flooded forests). 38

Supplementary Table 7. Estimated proportion of the number of animals hunted (Individuals Animals Offtake) per animal group in relation to the total offtake in upland terra firme forests (regions > 50 % of upland terra firme forests) and flooded forests (regions > 50 % of flooded forests). 40

Supplementary Data 4. Variable importance output of the 174 Taxon-Specific Offtake Proportion (TSOP) Random Forest models. For the purpose to show the variable importance for TSOP, we ran just one model with all the data. 42

Supplementary Table 8. Estimated proportion of the number of animals hunted (Individual Animals Offtake) for the 30 most hunted in relation to the total offtake in areas with < 70% of habitat loss and areas with > 70% of habitat loss. 100

Supplementary Discussion 1. The role of traditional wild meat food systems in advancing the Sustainable Development Goals (SDGs) in Amazonia. 102

Supplementary Method 4. Overview of primary data collection methods and related ethical procedures. 104

Supplementary Method 5. List of literature containing hunting data in rural areas of Amazonia. 107

Supplementary Data 5. Estimated densities (individuals per km^2) for 139 hunted taxa recorded in this study. 121

Supplementary Table 9. Mean, standard deviation, and sample size of energy, macro- and micronutrients per 100 g of wild meat in 26 hunted Amazonian species. 122

Supplementary Table 10. Daily values of Acceptable Macronutrient Distribution Range (AMDR), Estimated Average Requirement (EAR), Adequate Intake (AI), and Recommended Dietary Allowances (RDA) for protein, vitamin, minerals, and energy per life stage group. Source: IOM (1998[90], 2002[91], 2005[92]). 123

Supplementary Table 11. Daily values for energy per life stage based on Estimated Energy Requirements (EER). Source: IOM (2005)[92] 124

Supplementary Method 6. Formal endorsement by the Coordination of Indigenous Organizations of the Brazilian Amazon (COIAB) for the ethical aspects of primary data collection, article content, and participation in the Evaluation Committee for future research utilizing the Marupiara Dataset. 125

Supplementary Method 7. Formal endorsement by the National Council of Extractive Populations (CNS) for the ethical aspects of primary data collection, article content, and participation in the Evaluation Committee for future research utilizing the Marupiara Dataset. 126

Supplementary Method 1. Environmental and anthropogenic spatial variables used for modeling Harvest Productivity (HP) and Taxon-Specific Offtake Proportion (TSOP): Enhanced Vegetation Index (EVI) (A), Annual Gross Primary Productivity (GPP) (B), Annual Net Primary Productivity (NPP) (C), Soil Fertility (D), Proportion of flooded areas (E), Elevation (F), Height above the nearest drainage (HAND) (G), Historical distribution of Indigenous family languages (H), Current distribution of Indigenous and non-Indigenous peoples (I), Current distribution of family languages (J), Proportion of habitat loss (K) and Urban-rural catchment areas (URCA) (L). Amazonian regions: Guiana Shield (GS), north-western Amazonia (WAN), central Amazonia (CA), south-western Amazonia (WAS), southern Amazonia (SA), and eastern Amazonia (EA). See Methods for detailed information on the spatial prediction process.

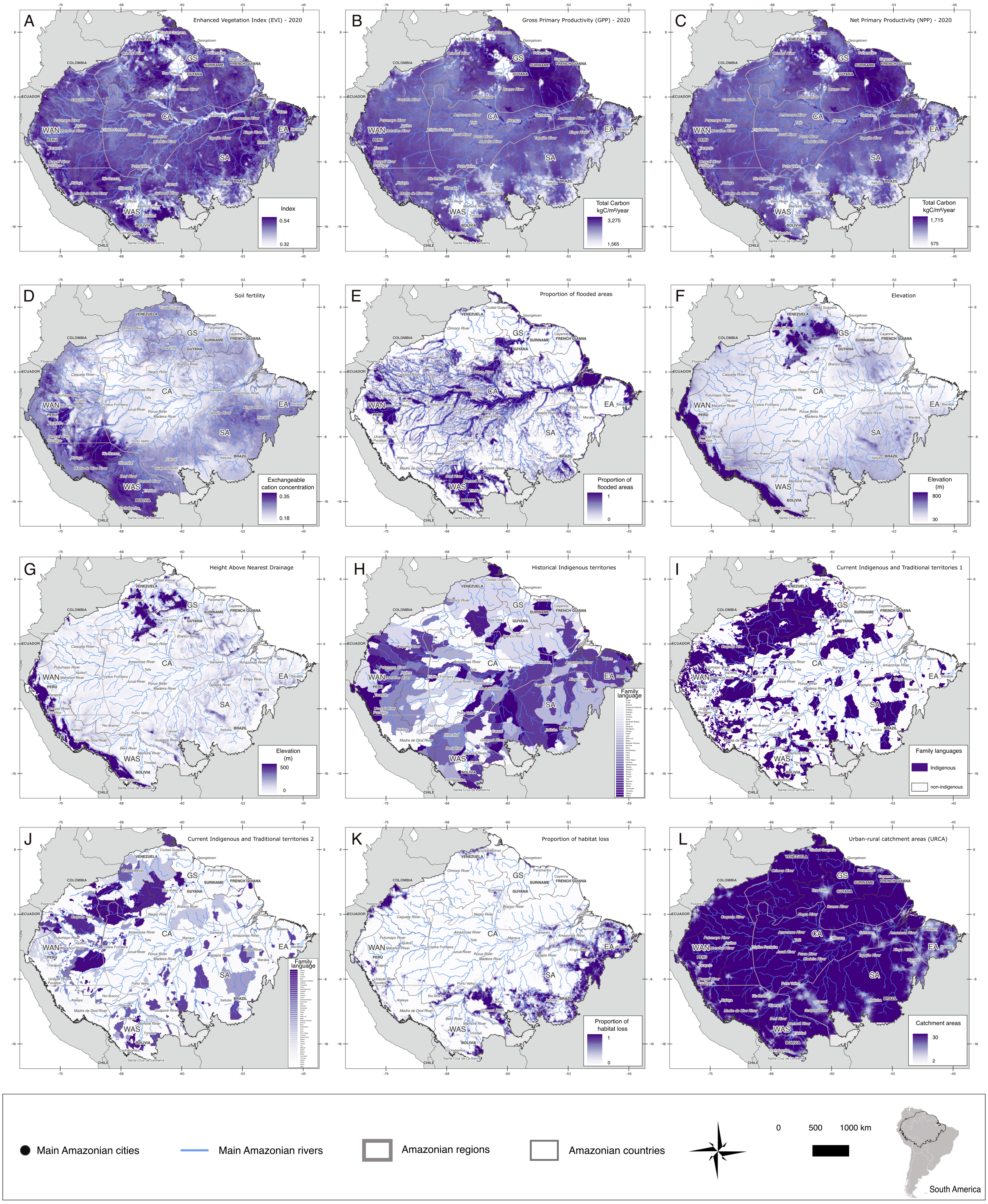

Supplementary Method 2. Historical distribution of Indigenous family languages in Amazonia. Raster produced based on maps provided in Loukotka (1967)[71] and Eriksen (2011)[72]. Amazonian regions: Guiana Shield (GS), north-western Amazonia (WAN), central Amazonia (CA), south-western Amazonia (WAS), southern Amazonia (SA), and eastern Amazonia (EA). See Methods for detailed information on the spatial prediction process.

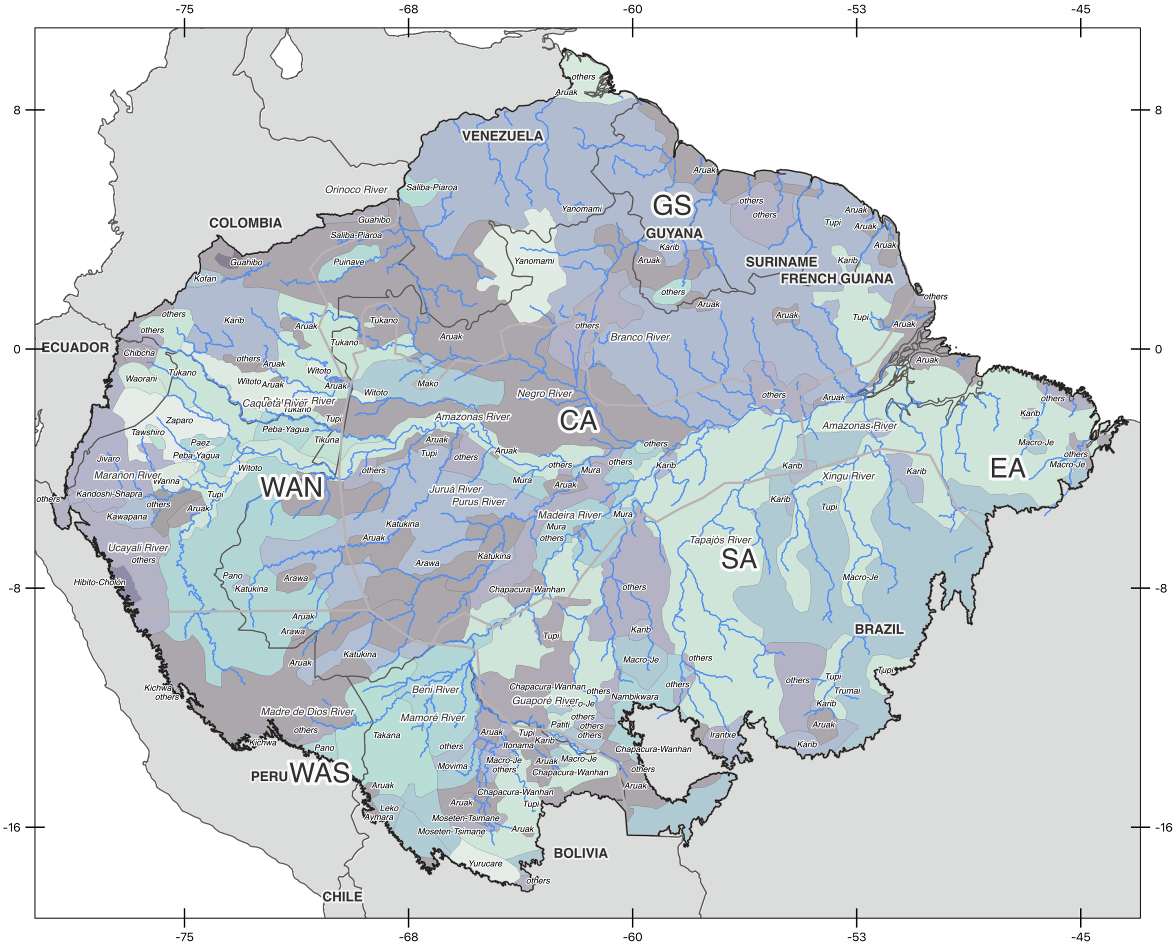

Supplementary Method 3. Current distribution of family languages. A categorical digital database built from the Amazon Network of Georeferenced Socio-Environmental Information (RAISG) database of the distribution of the Indigenous Lands[73], classified into their respective Indigenous family languages. Regions outside the Indigenous Lands are tentatively classified as Latin/German languages. Amazonian regions: Guiana Shield (GS), north-western Amazonia (WAN), central Amazonia (CA), south-western Amazonia (WAS), southern Amazonia (SA), and eastern Amazonia (EA). See Methods for detailed information on the spatial prediction process.

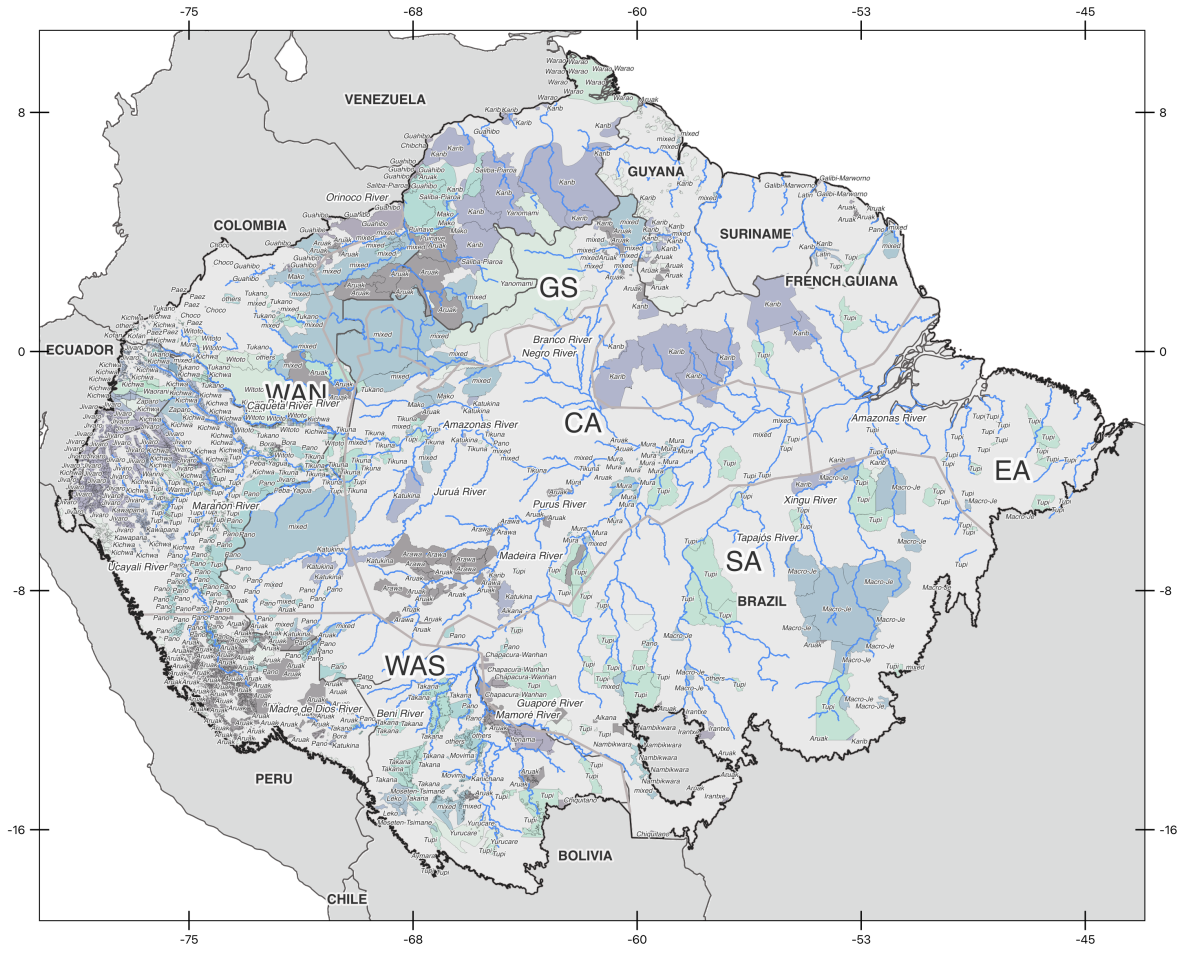

Supplementary Data 1. Variable importance output of the Overall Individual Animals Harvest Productivity (HP) Random Forest model. For the purpose to show the variable importance for HP, we ran just one model with all the data.

Soil fertility	0.00553
Annual Gross Primary Productivity (GPP)	0.00257
Elevation	0.00244
Annual Net Primary Productivity (NPP)	0.00237
Current distribution of family languages	0.00213
Hunting recording time span	0.0018
Urban-rural catchment areas (URCA)	0.00122
Historical distribution of Indigenous family languages	0.00104
Enhanced Vegetation Index (EVI)	0.00086
Proportion of habitat loss	0.00078
Current distribution of Indigenous and non-indigenous peoples	0.00031
Proportion of flooded areas	0.00027
Height above the nearest drainage (HAND)	0.00026
r2	0.222

Supplementary Data 2. Distribution of tetrapod vertebrate species (mammals, birds, reptiles and amphibians) richness in Amazonia. Raster representing the number of tetrapod species per pixel built using information from the IUCN spatial database (IUCN 2024)[20].

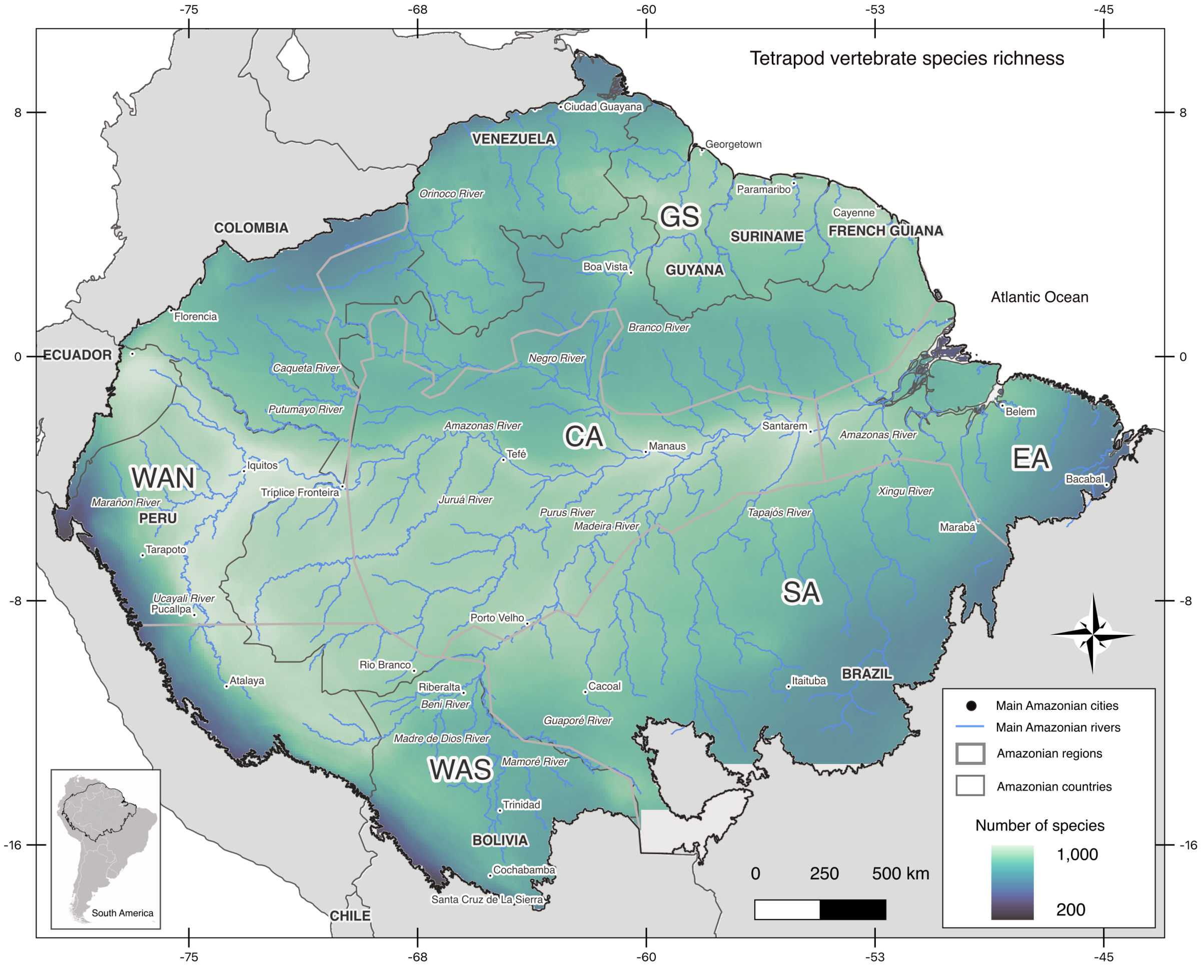

Supplementary Table 1. List of species hunted in Amazonia recorded in this study.

#	Species	Taxon	Class	Animal group
1	Desmodidae (non-identified sp.)	Chiroptera	Mammals	Bats
2	Molossidae (non-identified sp.)	Chiroptera	Mammals	Bats
3	Phyllostomus sp. 1	Chiroptera	Mammals	Bats
4	Phyllostomus sp. 2	Chiroptera	Mammals	Bats
5	Atelocynus microtis	Atelocynus microtis	Mammals	Canids
6	Cerdocyon thous	Cerdocyon thous	Mammals	Canids
7	Speothos venaticus	Speothos venaticus	Mammals	Canids
8	Inia geoffrensis	Inia geoffrensis	Mammals	Cetaceans
9	Sotalia fluviatilis	Sotalia fluviatilis	Mammals	Cetaceans
10	Cabassous unicinctus	Cabassous unicinctus	Mammals	Cingulates
11	Chaetophractus vellerosus	Chaetophractus vellerosus	Mammals	Cingulates
12	Chaetophractus villosus	Chaetophractus villosus	Mammals	Cingulates
13	Dasypus kappleri	Dasypus kappleri	Mammals	Cingulates
14	Dasypus novemcinctus	Dasypus novemcinctus	Mammals	Cingulates
15	Dasypus sabanicola	Dasypus sabanicola	Mammals	Cingulates
16	Dasypus septemcinctus	Dasypus septemcinctus	Mammals	Cingulates
17	Euphractus sexcinctus	Euphractus sexcinctus	Mammals	Cingulates
18	Priodontes maximus	Priodontes maximus	Mammals	Cingulates
19	Tolypeutes matacus	Tolypeutes matacus	Mammals	Cingulates
20	Tolypeutes tricinctus	Tolypeutes tricinctus	Mammals	Cingulates
21	Herpailurus yagouaroundi	Herpailurus yagouaroundi	Mammals	Felids
22	Leopardus pardalis	Leopardus pardalis	Mammals	Felids
23	Leopardus wiedii	Leopardus wiedii	Mammals	Felids
24	Panthera onca	Panthera onca	Mammals	Felids
25	Puma concolor	Puma concolor	Mammals	Felids
26	Bradypus tridactylus	Bradypus	Mammals	Folivores
27	Bradypus variegatus	Bradypus	Mammals	Folivores
28	Choloepus didactylus	Choloepus	Mammals	Folivores
29	Choloepus hoffmanni	Choloepus	Mammals	Folivores
30	Sylvilagus brasiliensis	Sylvilagus	Mammals	Lagomorphs
31	Sylvilagus floridanus	Sylvilagus	Mammals	Lagomorphs
32	Coendou bicolor	Coendou	Mammals	Large rodents
33	Coendou prehensilis	Coendou	Mammals	Large rodents
34	Cuniculus paca	Cuniculus paca	Mammals	Large rodents

#	Species	Taxon	Class	Animal group
35	Dasyprocta azarae	Dasyprocta	Mammals	Large rodents
36	Dasyprocta croconota	Dasyprocta	Mammals	Large rodents
37	Dasyprocta fuliginosa	Dasyprocta	Mammals	Large rodents
38	Dasyprocta leporina	Dasyprocta	Mammals	Large rodents
39	Dasyprocta prymnolopha	Dasyprocta	Mammals	Large rodents
40	Dasyprocta variegata	Dasyprocta	Mammals	Large rodents
41	Dinomys branickii	Dinomys branickii	Mammals	Large rodents
42	Galea musteloides	Galea	Mammals	Large rodents
43	Hydrochoerus hydrochaeris	Hydrochoerus hydrochaeris	Mammals	Large rodents
44	Myoprocta acouchy	Myoprocta	Mammals	Large rodents
45	Myoprocta pratti	Myoprocta	Mammals	Large rodents
46	Philander andersoni	Didelphidae (others)	Mammals	Marsupials
47	Didelphis marsupialis	Didelphis	Mammals	Marsupials
48	Eira barbara	Eira barbara	Mammals	Mustelids
49	Galictis vittata	Galictis vittata	Mammals	Mustelids
50	Lontra longicaudis	Lontra longicaudis	Mammals	Mustelids
51	Pteronura brasiliensis	Pteronura brasiliensis	Mammals	Mustelids
52	Cyclopes didactylus	Cyclopes didactylus	Mammals	Myrmecophagids
53	Myrmecophaga tridactyla	Myrmecophaga tridactyla	Mammals	Myrmecophagids
54	Tamandua tetradactyla	Tamandua tetradactyla	Mammals	Myrmecophagids
55	Alouatta belzebul	Alouatta	Mammals	Primates
56	Alouatta caraya	Alouatta	Mammals	Primates
57	Alouatta discolor	Alouatta	Mammals	Primates
58	Alouatta juara	Alouatta	Mammals	Primates
59	Alouatta macconnelli	Alouatta	Mammals	Primates
60	Alouatta nigerrima	Alouatta	Mammals	Primates
61	Alouatta puruensis	Alouatta	Mammals	Primates
62	Alouatta sara	Alouatta	Mammals	Primates
63	Alouatta seniculus	Alouatta	Mammals	Primates
64	Aotus azarae	Aotus	Mammals	Primates
65	Aotus nancymaae	Aotus	Mammals	Primates
66	Aotus nigriceps	Aotus	Mammals	Primates
67	Aotus trivirgatus	Aotus	Mammals	Primates

#	Species	Taxon	Class	Animal group
68	Aotus vociferans	Aotus	Mammals	Primates
69	Ateles belzebuth	Ateles	Mammals	Primates
70	Ateles chamek	Ateles	Mammals	Primates
71	Ateles marginatus	Ateles	Mammals	Primates
72	Ateles paniscus	Ateles	Mammals	Primates
73	Cacajao calvus	Cacajao	Mammals	Primates
74	Cacajao hosomi	Cacajao	Mammals	Primates
75	Cacajao melanocephalus	Cacajao	Mammals	Primates
76	Callimico goeldii	Callimico goeldii	Mammals	Primates
77	Cebuella niveiventris	Cebuella	Mammals	Primates
78	Cebuella pygmaea	Cebuella	Mammals	Primates
79	Cebus albifrons	Cebus	Mammals	Primates
80	Cebus castaneus	Cebus	Mammals	Primates
81	Cebus cuscinus	Cebus	Mammals	Primates
82	Cebus kaapori	Cebus	Mammals	Primates
83	Cebus leucocephalus	Cebus	Mammals	Primates
84	Cebus olivaceus	Cebus	Mammals	Primates
85	Cebus unicolor	Cebus	Mammals	Primates
86	Cebus yuracus	Cebus	Mammals	Primates
87	Cheracebus lucifer	Cheracebus	Mammals	Primates
88	Cheracebus lugens	Cheracebus	Mammals	Primates
89	Cheracebus purinus	Cheracebus	Mammals	Primates
90	Cheracebus torquatus	Cheracebus	Mammals	Primates
91	Chiropotes albinasus	Chiropotes	Mammals	Primates
92	Chiropotes sagulatus	Chiropotes	Mammals	Primates
93	Chiropotes satanas	Chiropotes	Mammals	Primates
94	Chiropotes utahicki	Chiropotes	Mammals	Primates
95	Lagothrix lagothricha cana	Lagothrix	Mammals	Primates
96	Lagothrix lagothricha flavicauda	Lagothrix	Mammals	Primates
97	Lagothrix lagothricha lagothricha	Lagothrix	Mammals	Primates
98	Lagothrix lagothricha lugens	Lagothrix	Mammals	Primates
99	Lagothrix lagothricha poeppigii	Lagothrix	Mammals	Primates
100	Lagothrix lagothricha tschudii	Lagothrix	Mammals	Primates
101	Lagothrix lagotricha lagotricha	Lagothrix	Mammals	Primates
102	Leontocebus fuscicollis	Leontocebus	Mammals	Primates

#	Species	Taxon	Class	Animal group
103	Leontocebus lagonotus	Leontocebus	Mammals	Primates
104	Leontocebus nigricollis graellsi	Leontocebus	Mammals	Primates
105	Leontocebus nigricollis nigricollis	Leontocebus	Mammals	Primates
106	Leontocebus sp.	Leontocebus	Mammals	Primates
107	Leontocebus tripartitus	Leontocebus	Mammals	Primates
108	Leontocebus weddelli melanoleucus	Leontocebus	Mammals	Primates
109	Leontocebus weddelli weddelli	Leontocebus	Mammals	Primates
110	Mico melanurus	Mico	Mammals	Primates
111	Mico sp.	Mico	Mammals	Primates
112	Pithecia aequatorialis	Pithecia	Mammals	Primates
113	Pithecia chrysocephala	Pithecia	Mammals	Primates
114	Pithecia hirsuta	Pithecia	Mammals	Primates
115	Pithecia irrorata	Pithecia	Mammals	Primates
116	Pithecia isabela	Pithecia	Mammals	Primates
117	Pithecia milleri	Pithecia	Mammals	Primates
118	Pithecia mittermeieri	Pithecia	Mammals	Primates
119	Pithecia monachus	Pithecia	Mammals	Primates
120	Pithecia napensis	Pithecia	Mammals	Primates
121	Pithecia pithecia	Pithecia	Mammals	Primates
122	Pithecia vanzolinii	Pithecia	Mammals	Primates
123	Plecturocebus aureipalatii	Plecturocebus	Mammals	Primates
124	Plecturocebus cupreus	Plecturocebus	Mammals	Primates
125	Plecturocebus discolor	Plecturocebus	Mammals	Primates
126	Plecturocebus donacophilus	Plecturocebus	Mammals	Primates
127	Plecturocebus hoffmannsi	Plecturocebus	Mammals	Primates
128	Plecturocebus toppini	Plecturocebus	Mammals	Primates
129	Plecturocebus urubambensis	Plecturocebus	Mammals	Primates
130	Saguinus imperator	Saguinus	Mammals	Primates
131	Saguinus imperator subgrisescens	Saguinus	Mammals	Primates
132	Saguinus inustus	Saguinus	Mammals	Primates
133	Saguinus midas	Saguinus	Mammals	Primates
134	Saimiri boliviensis boliviensis	Saimiri	Mammals	Primates
135	Saimiri boliviensis peruviensis	Saimiri	Mammals	Primates

#	Species	Taxon	Class	Animal group
136	Saimiri cassiquiarensis cassiquiarensis	Saimiri	Mammals	Primates
137	Saimiri cassiquiarensis macrodon	Saimiri	Mammals	Primates
138	Saimiri sciureus	Saimiri	Mammals	Primates
139	Sapajus apella	Sapajus	Mammals	Primates
140	Bassaricyon alleni	Bassaricyon	Mammals	Procyonids
141	Bassaricyon beddardi	Bassaricyon	Mammals	Procyonids
142	Bassaricyon gabbi	Bassaricyon	Mammals	Procyonids
143	Nasua nasua	Nasua nasua	Mammals	Procyonids
144	Nasuella olivacea	Nasuella olivacea	Mammals	Procyonids
145	Potos flavus	Potos flavus	Mammals	Procyonids
146	Procyon cancrivorus	Procyon cancrivorus	Mammals	Procyonids
147	Trichechus inunguis	Trichechus inunguis	Mammals	Sirenians
148	Cavia aperea	Cavia	Mammals	Small rodents
149	Cricetidae (non-identified sp. 1)	Cricetidae	Mammals	Small rodents
150	Cricetidae (non-identified sp. 2)	Cricetidae	Mammals	Small rodents
151	Cricetidae (non-identified sp. 3)	Cricetidae	Mammals	Small rodents
152	Holochilus sciureus	Cricetidae	Mammals	Small rodents
153	Dactylomys dactylinus	Echimyidae	Mammals	Small rodents
154	Makalata macrura	Echimyidae	Mammals	Small rodents
155	Mesomys hispidus	Echimyidae	Mammals	Small rodents
156	Proechimys quadruplicatus	Echimyidae	Mammals	Small rodents
157	Proechimys semispinosus	Echimyidae	Mammals	Small rodents
158	Proechimys simonsi	Echimyidae	Mammals	Small rodents
159	Toromys grandis	Echimyidae	Mammals	Small rodents
160	Microsciurus flaviventer	Microsciurus flaviventer	Mammals	Small rodents
161	Sciurus (Hadrosciurus) igniventris	Sciurus (Hadrosciurus)	Mammals	Small rodents
162	Sciurus (Hadrosciurus) spadiceus	Sciurus (Hadrosciurus)	Mammals	Small rodents
163	Sciurus (Notosciurus) aestuans	Sciurus (Notosciurus)	Mammals	Small rodents
164	Sciurus (Notosciurus) granatensis	Sciurus (Notosciurus)	Mammals	Small rodents
165	Sciurus (Notosciurus) ignitus	Sciurus (Notosciurus)	Mammals	Small rodents
166	Blastocerus dichotomus	Blastocerus dichotomus	Mammals	Ungulates
167	Dicotyles tajacu	Dicotyles tajacu	Mammals	Ungulates
168	Mazama americana	Mazama americana	Mammals	Ungulates
169	Mazama gouazoubira	Mazama gouazoubira	Mammals	Ungulates
170	Mazama nemorivaga	Mazama nemorivaga	Mammals	Ungulates

#	Species	Taxon	Class	Animal group
171	Odocoileus virginianus	Odocoileus virginianus	Mammals	Ungulates
172	Ozotoceros bezoarticus	Ozotoceros bezoarticus	Mammals	Ungulates
173	Tapirus terrestris	Tapirus terrestris	Mammals	Ungulates
174	Tayassu pecari	Tayassu pecari	Mammals	Ungulates
175	Tremarctos ornatus	Tremarctos ornatus	Mammals	Ursid
176	Chloroceryle americana	Alcedinidae	Birds	Aquatic birds
177	Chloroceryle sp.	Alcedinidae	Birds	Aquatic birds
178	Megaceryle torquata	Alcedinidae	Birds	Aquatic birds
179	Amazonetta brasiliensis	Anatidae (others)	Birds	Aquatic birds
180	Anas bahamensis	Anatidae (others)	Birds	Aquatic birds
181	Anas platyrhynchos	Anatidae (others)	Birds	Aquatic birds
182	Dendrocygna bicolor	Anatidae (others)	Birds	Aquatic birds
183	Nomonyx dominicus	Anatidae (others)	Birds	Aquatic birds
184	Anhima cornuta	Anhima cornuta	Birds	Aquatic birds
185	Anhinga anhinga	Anhinga anhinga	Birds	Aquatic birds
186	Aramus guarauna	Aramidae	Birds	Aquatic birds
187	Ardea alba	Ardea alba	Birds	Aquatic birds
188	Ardea cocoi	Ardea cocoi	Birds	Aquatic birds
189	Agamia agami	Ardeidae (others)	Birds	Aquatic birds
190	Botaurus pinnatus	Ardeidae (others)	Birds	Aquatic birds
191	Bubulcus ibis	Ardeidae (others)	Birds	Aquatic birds
192	Butorides striata	Ardeidae (others)	Birds	Aquatic birds
193	Cochlearius cochlearius	Ardeidae (others)	Birds	Aquatic birds
194	Egretta caerulea	Ardeidae (others)	Birds	Aquatic birds
195	Egretta thula	Ardeidae (others)	Birds	Aquatic birds
196	Nyctanassa violacea	Ardeidae (others)	Birds	Aquatic birds
197	Nycticorax nycticorax	Ardeidae (others)	Birds	Aquatic birds
198	Pilherodius pileatus	Ardeidae (others)	Birds	Aquatic birds
199	Syrigma sibilatrix	Ardeidae (others)	Birds	Aquatic birds
200	Tigrisoma fasciatum	Ardeidae (others)	Birds	Aquatic birds
201	Tigrisoma lineatum	Ardeidae (others)	Birds	Aquatic birds
202	Burhinus bistriatus	Burhinus bistriatus	Birds	Aquatic birds
203	Cairina moschata	Cairina moschata	Birds	Aquatic birds

#	Species	Taxon	Class	Animal group
204	Chauna torquata	Chauna torquata	Birds	Aquatic birds
205	Ciconia maguari	Ciconia maguari	Birds	Aquatic birds
206	Dendrocygna autumnalis	Dendrocygna autumnalis	Birds	Aquatic birds
207	Eudocimus ruber	Eudocimus ruber	Birds	Aquatic birds
208	Eurypyga helias	Eurypyga helias	Birds	Aquatic birds
209	Heliornis fulica	Heliornis fulica	Birds	Aquatic birds
210	Jabiru mycteria	Jabiru mycteria	Birds	Aquatic birds
211	Jacana jacana	Jacana jacana	Birds	Aquatic birds
212	Mesembrinibis cayennensis	Mesembrinibis cayennensis	Birds	Aquatic birds
213	Mycteria americana	Mycteria americana	Birds	Aquatic birds
214	Nannopterum brasilianus	Nannopterum brasilianus	Birds	Aquatic birds
215	Neochen jubata	Neochen jubata	Birds	Aquatic birds
216	Opisthocomus hoazin	Opisthocomus hoazin	Birds	Aquatic birds
217	Phoenicoparrus sp.	Phoenicoparrus	Birds	Aquatic birds
218	Aramides cajaneus	Rallidae	Birds	Aquatic birds
219	Porphyrio flavirostris	Rallidae	Birds	Aquatic birds
220	Porphyrio martinica	Rallidae	Birds	Aquatic birds
221	Numenius phaeopus	Scolopacidae	Birds	Aquatic birds
222	Scolopacidae (non-identified sp. 1)	Scolopacidae	Birds	Aquatic birds
223	Scolopacidae (non-identified sp. 2)	Scolopacidae	Birds	Aquatic birds
224	Scolopacidae (non-identified sp. 3)	Scolopacidae	Birds	Aquatic birds
225	Scolopacidae (non-identified sp. 4)	Scolopacidae	Birds	Aquatic birds
226	Scolopacidae (non-identified sp. 5)	Scolopacidae	Birds	Aquatic birds
227	Scolopacidae (non-identified sp. 6)	Scolopacidae	Birds	Aquatic birds
228	Tringa flavipes	Scolopacidae	Birds	Aquatic birds
229	Spatula discors	Spatula discors	Birds	Aquatic birds
230	Theristicus caudatus	Theristicus caudatus	Birds	Aquatic birds
231	Threskiornithidae	Threskiornithidae (others)	Birds	Aquatic birds
232	Claravis pretiosa	Columbidae (others)	Birds	Columbids
233	Columbina minuta	Columbidae (others)	Birds	Columbids
234	Columbina talpacoti	Columbidae (others)	Birds	Columbids
235	Geotrygon montana	Columbidae (others)	Birds	Columbids
236	Leptotila rufaxilla	Columbidae (others)	Birds	Columbids
237	Leptotila verreauxi	Columbidae (others)	Birds	Columbids
238	Zenaida auriculata	Columbidae (others)	Birds	Columbids

#	Species	Taxon	Class	Animal group
239	Patagioenas cayennensis	Patagioenas	Birds	Columbids
240	Patagioenas plumbea	Patagioenas	Birds	Columbids
241	Patagioenas speciosa	Patagioenas	Birds	Columbids
242	Patagioenas subvinacea	Patagioenas	Birds	Columbids
243	Chamaepetes goudotii	Chamaepetes goudotii	Birds	Cracids
244	Crax alector	Crax	Birds	Cracids
245	Crax fasciolata	Crax	Birds	Cracids
246	Crax globulosa	Crax	Birds	Cracids
247	Crax pinima	Crax	Birds	Cracids
248	Mitu salvini	Mitu	Birds	Cracids
249	Mitu tomentosum	Mitu	Birds	Cracids
250	Mitu tuberosum	Mitu	Birds	Cracids
251	Nothocrax urumutum	Nothocrax urumutum	Birds	Cracids
252	Ortalis guttata	Ortalis	Birds	Cracids
253	Ortalis motmot	Ortalis	Birds	Cracids
254	Penelope jacquacu	Penelope	Birds	Cracids
255	Penelope marail	Penelope	Birds	Cracids
256	Penelope montagnii	Penelope	Birds	Cracids
257	Penelope obscura	Penelope	Birds	Cracids
258	Penelope pileata	Penelope	Birds	Cracids
259	Penelope purpurascens	Penelope	Birds	Cracids
260	Penelope superciliaris	Penelope	Birds	Cracids
261	Pipile cujubi	Pipile	Birds	Cracids
262	Pipile cumanensis	Pipile	Birds	Cracids
263	Pipile grayi	Pipile	Birds	Cracids
264	Amazona aestiva	Amazona	Birds	Large psittacids
265	Amazona amazonica	Amazona	Birds	Large psittacids
266	Amazona farinosa	Amazona	Birds	Large psittacids
267	Amazona festiva	Amazona	Birds	Large psittacids
268	Amazona mercenarius	Amazona	Birds	Large psittacids
269	Amazona ochrocephala	Amazona	Birds	Large psittacids
270	Anodorhynchus hyacinthinus	Anodorhynchus hyacinthinus	Birds	Large psittacids
271	Ara ararauna	Ara	Birds	Large psittacids

#	Species	Taxon	Class	Animal group
272	Ara chloropterus	Ara	Birds	Large psittacids
273	Ara macao	Ara	Birds	Large psittacids
274	Ara militaris	Ara	Birds	Large psittacids
275	Ara severus	Ara	Birds	Large psittacids
276	Nyctibius grandis	miscellaneous	Birds	miscellaneous
277	Pteroglossus aracari	Pteroglossus	Birds	Ramphastids
278	Pteroglossus azara	Pteroglossus	Birds	Ramphastids
279	Pteroglossus beauharnaisii	Pteroglossus	Birds	Ramphastids
280	Pteroglossus castanotis	Pteroglossus	Birds	Ramphastids
281	Pteroglossus flavirostris	Pteroglossus	Birds	Ramphastids
282	Pteroglossus inscriptus	Pteroglossus	Birds	Ramphastids
283	Pteroglossus pluricinctus	Pteroglossus	Birds	Ramphastids
284	Pteroglossus viridis	Pteroglossus	Birds	Ramphastids
285	Ramphastos culminatus	Ramphastos	Birds	Ramphastids
286	Ramphastos cuvieri	Ramphastos	Birds	Ramphastids
287	Ramphastos toco	Ramphastos	Birds	Ramphastids
288	Ramphastos tucanus	Ramphastos	Birds	Ramphastids
289	Ramphastos vitellinus	Ramphastos	Birds	Ramphastids
290	Selenidera nattereri	Selenidera	Birds	Ramphastids
291	Selenidera piperivora	Selenidera	Birds	Ramphastids
292	Selenidera reinwardtii	Selenidera	Birds	Ramphastids
293	Accipiter poliogaster	Accipitridae	Birds	Raptor birds
294	Accipiter sp. 1	Accipitridae	Birds	Raptor birds
295	Accipiter sp. 2	Accipitridae	Birds	Raptor birds
296	Busarellus nigricollis	Accipitridae	Birds	Raptor birds
297	Buteo nitidus	Accipitridae	Birds	Raptor birds
298	Chondrohierax uncinatus	Accipitridae	Birds	Raptor birds
299	Geranospiza caerulescens	Accipitridae	Birds	Raptor birds
300	Harpagus bidentatus	Accipitridae	Birds	Raptor birds
301	Ictinia plumbea	Accipitridae	Birds	Raptor birds
302	Pandion haliaetus	Accipitridae	Birds	Raptor birds
303	Rostrhamus sociabilis	Accipitridae	Birds	Raptor birds
304	Rupornis magnirostris	Accipitridae	Birds	Raptor birds
305	Spizaetus melanoleucus	Accipitridae	Birds	Raptor birds
306	Spizaetus ornatus	Accipitridae	Birds	Raptor birds

#	Species	Taxon	Class	Animal group
307	Spizaetus tyrannus	Accipitridae	Birds	Raptor birds
308	Accipitridae, Falconidae ni	Accipitridae, Falconidae	Birds	Raptor birds
309	Cathartes aura	Cathartidae (others)	Birds	Raptor birds
310	Caracara plancus	Falconidae	Birds	Raptor birds
311	Daptrius ater	Falconidae	Birds	Raptor birds
312	Ibycter americanus	Falconidae	Birds	Raptor birds
313	Micrastur mirandollei	Falconidae	Birds	Raptor birds
314	Micrastur semitorquatus	Falconidae	Birds	Raptor birds
315	Milvago chimachima	Falconidae	Birds	Raptor birds
316	Phalcoboenus sp.	Falconidae	Birds	Raptor birds
317	Harpia harpyja	Harpia harpyja	Birds	Raptor birds
318	Sarcoramphus papa	Sarcoramphus papa	Birds	Raptor birds
319	Glaucidium brasilianum	Strigidae	Birds	Raptor birds
320	Megascops choliba	Strigidae	Birds	Raptor birds
321	Pseudoscops clamator	Strigidae	Birds	Raptor birds
322	Pulsatrix perspicillata	Strigidae	Birds	Raptor birds
323	Strigidae	Strigidae	Birds	Raptor birds
324	Tyto alba	Strigidae	Birds	Raptor birds
325	Bucconidae	Bucconidae	Birds	Small birds
326	Monasa nigrifrons	Bucconidae	Birds	Small birds
327	Cotinga cayana	Cotingidae	Birds	Small birds
328	Cotinga cotinga	Cotingidae	Birds	Small birds
329	Lipaugus vociferans	Cotingidae	Birds	Small birds
330	Perissocephalus tricolor	Cotingidae	Birds	Small birds
331	Phoenicircus carnifex	Cotingidae	Birds	Small birds
332	Phoenicircus nigricollis	Cotingidae	Birds	Small birds
333	Querula purpurata	Cotingidae	Birds	Small birds
334	Xipholena punicea	Cotingidae	Birds	Small birds
335	Coccyzus americanus	Cuculidae	Birds	Small birds
336	Crotophaga ani	Cuculidae	Birds	Small birds
337	Crotophaga major	Cuculidae	Birds	Small birds
338	Cuculidae ni	Cuculidae	Birds	Small birds
339	Neomorphus pucheranii	Cuculidae	Birds	Small birds

#	Species	Taxon	Class	Animal group
340	Piaya cayana	Cuculidae	Birds	Small birds
341	Piaya sp.	Cuculidae	Birds	Small birds
342	Electron platyrhynchum	Momotidae	Birds	Small birds
343	Momotidae ni	Momotidae	Birds	Small birds
344	Momotus momota	Momotidae	Birds	Small birds
345	Ammodramus humeralis	Passeriformes (others)	Birds	Small birds
346	Cacicus cela	Passeriformes (others)	Birds	Small birds
347	Cacicus oseryi	Passeriformes (others)	Birds	Small birds
348	Cacicus sp.	Passeriformes (others)	Birds	Small birds
349	Campylorhynchus turdinus	Passeriformes (others)	Birds	Small birds
350	Chlorophanes spiza	Passeriformes (others)	Birds	Small birds
351	Cissopis leveriana	Passeriformes (others)	Birds	Small birds
352	Cyanocorax cayanus	Passeriformes (others)	Birds	Small birds
353	Cyanocorax violaceus	Passeriformes (others)	Birds	Small birds
354	Cyanolyca viridicyana	Passeriformes (others)	Birds	Small birds
355	Dacnis lineata	Passeriformes (others)	Birds	Small birds
356	Formicarius sp.	Passeriformes (others)	Birds	Small birds
357	Glyphorynchus spirurus	Passeriformes (others)	Birds	Small birds
358	Hirundinidae ni	Passeriformes (others)	Birds	Small birds
359	Icterus cayanensis	Passeriformes (others)	Birds	Small birds
360	Icterus chrysocephalus	Passeriformes (others)	Birds	Small birds
361	Molothrus oryzivorus	Passeriformes (others)	Birds	Small birds
362	Myiarchus tuberculifer	Passeriformes (others)	Birds	Small birds
363	Passeriformes ni	Passeriformes (others)	Birds	Small birds
364	Pipridae ni	Passeriformes (others)	Birds	Small birds
365	Psarocolius oseryi	Passeriformes (others)	Birds	Small birds
366	Ramphocelus carbo	Passeriformes (others)	Birds	Small birds
367	Ramphocelus nigrogularis	Passeriformes (others)	Birds	Small birds
368	Sporophila angolensis	Passeriformes (others)	Birds	Small birds
369	Tangara chilensis	Passeriformes (others)	Birds	Small birds
370	Tangara episcopus	Passeriformes (others)	Birds	Small birds
371	Tangara schrankii	Passeriformes (others)	Birds	Small birds
372	Thamnophilus sp.	Passeriformes (others)	Birds	Small birds
373	Turdus sp.	Passeriformes (others)	Birds	Small birds
374	Tyrannulus elatus	Passeriformes (others)	Birds	Small birds

#	Species	Taxon	Class	Animal group
375	Tyrannus melancholicus	Passeriformes (others)	Birds	Small birds
376	Xiphorhynchus sp.	Passeriformes (others)	Birds	Small birds
377	Campephilus gayaquilensis	Picidae	Birds	Small birds
378	Campephilus melanoleucos	Picidae	Birds	Small birds
379	Campephilus rubricollis	Picidae	Birds	Small birds
380	Celeus elegans	Picidae	Birds	Small birds
381	Celeus flavus	Picidae	Birds	Small birds
382	Dryocopus lineatus	Picidae	Birds	Small birds
383	Melanerpes cruentatus	Picidae	Birds	Small birds
384	Picidae ni	Picidae	Birds	Small birds
385	Piculus elegans	Picidae	Birds	Small birds
386	Pionus fuscus	Pionus	Birds	Small birds
387	Pionus menstruus	Pionus	Birds	Small birds
388	Psarocolius angustifrons	Psarocolius	Birds	Small birds
389	Psarocolius atrovirens	Psarocolius	Birds	Small birds
390	Psarocolius bifasciatus	Psarocolius	Birds	Small birds
391	Psarocolius decumanus	Psarocolius	Birds	Small birds
392	Psarocolius viridis	Psarocolius	Birds	Small birds
393	Aratinga weddellii	Psittacidae (others)	Birds	Small birds
394	Brotogeris chrysoptera	Psittacidae (others)	Birds	Small birds
395	Brotogeris cyanoptera	Psittacidae (others)	Birds	Small birds
396	Brotogeris sanctithomae	Psittacidae (others)	Birds	Small birds
397	Deroptyus accipitrinus	Psittacidae (others)	Birds	Small birds
398	Graydidascalus brachyurus	Psittacidae (others)	Birds	Small birds
399	Gypopsitta caica	Psittacidae (others)	Birds	Small birds
400	Orthopsittaca manilata	Psittacidae (others)	Birds	Small birds
401	Pionites melanocephalus	Psittacidae (others)	Birds	Small birds
402	Psittacara leucophthalmus	Psittacidae (others)	Birds	Small birds
403	Pyrilia barrabandi	Psittacidae (others)	Birds	Small birds
404	Pyrrhura melanura	Psittacidae (others)	Birds	Small birds
405	Pyrrhura picta	Psittacidae (others)	Birds	Small birds
406	Touit purpuratus	Psittacidae (others)	Birds	Small birds
407	Florisuga mellivora	Trochilidae	Birds	Small birds

#	Species	Taxon	Class	Animal group
408	Trochilidae (non-identified sp. 1)	Trochilidae	Birds	Small birds
409	Trochilidae (non-identified sp. 2)	Trochilidae	Birds	Small birds
410	Trochilidae (non-identified sp. 3)	Trochilidae	Birds	Small birds
411	Trogon melanurus	Trogonidae	Birds	Small birds
412	Trogon sp.	Trogonidae	Birds	Small birds
413	Trogon viridis	Trogonidae	Birds	Small birds
414	Cariama cristata	Cariama cristata	Birds	Terrestrial birds
415	Colinus cristatus	Colinus cristatus	Birds	Terrestrial birds
416	Crypturellus atrocapillus	Crypturellus	Birds	Terrestrial birds
417	Crypturellus brevirostris	Crypturellus	Birds	Terrestrial birds
418	Crypturellus cinereus	Crypturellus	Birds	Terrestrial birds
419	Crypturellus duidae	Crypturellus	Birds	Terrestrial birds
420	Crypturellus erythropus	Crypturellus	Birds	Terrestrial birds
421	Crypturellus parvirostris	Crypturellus	Birds	Terrestrial birds
422	Crypturellus soui	Crypturellus	Birds	Terrestrial birds
423	Crypturellus strigulosus	Crypturellus	Birds	Terrestrial birds
424	Crypturellus undulatus	Crypturellus	Birds	Terrestrial birds
425	Crypturellus variegatus	Crypturellus	Birds	Terrestrial birds
426	Odontophorus erythrops	Odontophorus	Birds	Terrestrial birds
427	Odontophorus gujanensis	Odontophorus	Birds	Terrestrial birds
428	Odontophorus stellatus	Odontophorus	Birds	Terrestrial birds
429	Psophia crepitans	Psophia	Birds	Terrestrial birds
430	Psophia dextralis	Psophia	Birds	Terrestrial birds
431	Psophia leucoptera	Psophia	Birds	Terrestrial birds
432	Psophia napensis	Psophia	Birds	Terrestrial birds
433	Psophia obscura	Psophia	Birds	Terrestrial birds
434	Psophia ochroptera	Psophia	Birds	Terrestrial birds
435	Psophia viridis	Psophia	Birds	Terrestrial birds
436	Rhea americana	Rhea americana	Birds	Terrestrial birds
437	Tinamus guttatus	Tinamus	Birds	Terrestrial birds
438	Tinamus major	Tinamus	Birds	Terrestrial birds
439	Tinamus tao	Tinamus	Birds	Terrestrial birds
440	Caiman crocodilus	Caiman crocodilus	Reptiles	Caimans
441	Caiman yacare	Caiman yacare	Reptiles	Caimans
442	Melanosuchus niger	Melanosuchus niger	Reptiles	Caimans

#	Species	Taxon	Class	Animal group
443	Paleosuchus palpebrosus	Paleosuchus palpebrosus	Reptiles	Caimans
444	Paleosuchus trigonatus	Paleosuchus trigonatus	Reptiles	Caimans
445	Ameiva ameiva	Ameiva ameiva	Reptiles	Lizards
446	Dracaena guianensis	Dracaena guianensis	Reptiles	Lizards
447	Iguana iguana	Iguana iguana	Reptiles	Lizards
448	Lacertilia (non-identified sp. 1)	Lacertilia (others)	Reptiles	Lizards
449	Lacertilia (non-identified sp. 2)	Lacertilia (others)	Reptiles	Lizards
450	Lacertilia (non-identified sp. 3)	Lacertilia (others)	Reptiles	Lizards
451	Lacertilia (non-identified sp. 4)	Lacertilia (others)	Reptiles	Lizards
452	Tupinambis teguixin	Tupinambis teguixin	Reptiles	Lizards
453	Chelus fimbriata	Chelus fimbriata	Reptiles	River turtles
454	Kinosternon scorpioides	Kinosternon scorpioides	Reptiles	River turtles
455	Mesoclemmys gibba	Mesoclemmys	Reptiles	River turtles
456	Mesoclemmys nasuta	Mesoclemmys	Reptiles	River turtles
457	Mesoclemmys raniceps	Mesoclemmys	Reptiles	River turtles
458	Phrynops geoffroanus	Phrynops	Reptiles	River turtles
459	Peltocephalus dumerilianus	Peltocephalus dumerilianus	Reptiles	River turtles
460	Platemys platycephala	Platemys platycephala	Reptiles	River turtles
461	Podocnemis erythrocephala	Podocnemis erythrocephala	Reptiles	River turtles
462	Podocnemis expansa	Podocnemis expansa	Reptiles	River turtles
463	Podocnemis sextuberculata	Podocnemis sextuberculata	Reptiles	River turtles
464	Podocnemis unifilis	Podocnemis unifilis	Reptiles	River turtles
465	Podocnemis vogli	Podocnemis vogli	Reptiles	River turtles
466	Rhinemys rufipes	Rhinemys rufipes	Reptiles	River turtles
467	Rhinoclemmys punctularia	Rhinoclemmys punctularia	Reptiles	River turtles
468	Boa constrictor	Boa constrictor	Reptiles	Snakes
469	Eunectes murinus	Eunectes murinus	Reptiles	Snakes
470	Bothrops atrox	Serpentes (others)	Reptiles	Snakes
471	Bothrops sp.	Serpentes (others)	Reptiles	Snakes
472	Colubridae ni	Serpentes (others)	Reptiles	Snakes
473	Lachesis muta	Serpentes (others)	Reptiles	Snakes
474	Serpentes (non-identified sp. 1)	Serpentes (others)	Reptiles	Snakes
475	Serpentes (non-identified sp. 2)	Serpentes (others)	Reptiles	Snakes

#	Species	Taxon	Class	Animal group
476	Serpentes (non-identified sp. 3)	Serpentes (others)	Reptiles	Snakes
477	Serpentes (non-identified sp. 4)	Serpentes (others)	Reptiles	Snakes
478	Chelonoidis carbonaria	Chelonoidis	Reptiles	Tortoises
479	Chelonoidis denticulata	Chelonoidis	Reptiles	Tortoises
480	Anura ni	Anura	Anurans	Frogs
481	Boana wavrini	Anura	Anurans	Frogs
482	Leptodactylus knudseni	Anura	Anurans	Frogs
483	Leptodactylus pentadactylus	Anura	Anurans	Frogs
484	Leptodactylus rhodomystax	Anura	Anurans	Frogs
485	Leptodactylus riveroi	Anura	Anurans	Frogs
486	Leptodactylus sp.	Anura	Anurans	Frogs
487	Osteocephalus cabrerai	Anura	Anurans	Frogs
488	Osteocephalus taurinus	Anura	Anurans	Frogs
489	Osteocephalus yasuni	Anura	Anurans	Frogs
490	Rhinella marina	Anura	Anurans	Frogs

Supplementary Table 2. Estimated number of individual animals hunted, animal biomass extracted (kg), body mass (kg), density (ind/km²), geographic distribution area in Amazonia (km²) and number of animals hunted/km² per taxon per year in Amazonia.

Taxon	Class	Group	Individual animals hunted	Animal biomass extracted (kg)	Body mass (kg)	Density (ind/km²)	Geographic distribution area (km²)
Cuniculus paca	Mammals	Large rodents	5,938,596 ± 874,536 (5,123,625 – 6,818,663)	43,554,384 ± 7,366,151 (36,662,532 – 51,013,860)	7.32 ± 0.17 (7.10 – 7.54)	42.3 ± 6.16 (34.3 – 50.5)	7,983,800
Tayassu pecari	Mammals	Ungulates	4,591,132 ± 897,156 (3,725,897 – 5,493,987)	140,253,664 ± 35,355,398 (106,276,589 – 176,532,142)	30.3 ± 1.79 (28.1 – 32.6)	4.50 ± 1.05 (3.10 – 5.81)	8,052,400
Dasyprocta	Mammals	Large rodents	3,415,417 ± 671,689 (2,770,711 – 4,089,073)	12,754,437 ± 2,985,116 (9,887,083 – 15,785,200)	3.71 ± 0.14 (3.53 – 3.90)	28.4 ± 8.01 (18.2 – 38.9)	8,037,900
Dicotyles tajacu	Mammals	Ungulates	3,167,409 ± 529,075 (2,663,354 – 3,699,319)	63,064,464 ± 12,713,877 (50,924,263 – 76,006,042)	19.8 ± 0.71 (18.9 – 20.7)	5.15 ± 0.60 (4.41 – 5.9)	7,907,600
undetermined	—	—	1,981,245 ± 376,243 (1,631,757 – 2,367,046)	3,885,101 ± 1,914,609 (2,042,983 – 5,884,599)	1.87 ± 0.58 (1.11 – 2.61)	—	8,045,900
Dasypus novemcinctus	Mammals	Cingulates	1,833,856 ± 303,121 (1,551,833 – 2,141,756)	8,094,542 ± 1,689,691 (6,510,593 – 9,827,586)	4.39 ± 0.21 (4.13 – 4.64)	15.8 ± 4.86 (9.67 – 22.0)	8,002,500
Chelonoidis	Reptiles	Tortoises	1,795,628 ± 572,142 (1,230,348 – 2,354,694)	10,832,235 ± 4,648,309 (6,326,642 – 15,575,566)	5.85 ± 0.70 (4.93 – 6.79)	61.6 ± 30.6 (22.7 – 103.0)	7,438,300
Penelope	Birds	Cracids	1,696,518 ± 390,067 (1,322,211 – 2,092,206)	2,592,928 ± 686,813 (1,936,323 – 3,298,299)	1.52 ± 0.05 (1.45 – 1.59)	10.3 ± 2.59 (6.82 – 13.5)	8,091,700
Mazama americana	Mammals	Ungulates	1,389,358 ± 423,066 (962,164 – 1,787,458)	39,788,301 ± 13,470,724 (26,258,677 – 52,786,009)	28.4 ± 1.10 (26.9 – 29.8)	1.71 ± 0.93 (0.41 – 2.95)	7,973,500
Mitu	Birds	Cracids	1,098,793 ± 171,075 (939,705 – 1,272,803)	3,647,936 ± 687,741 (3,004,938 – 4,352,772)	3.31 ± 0.11 (3.16 – 3.46)	5.15 ± 0.96 (3.93 – 6.38)	6,445,600
Podocnemis unifilis	Reptiles	River turtles	1,020,806 ± 224,157 (815,022 – 1,251,670)	6,341,413 ± 2,075,966 (4,423,345 – 8,521,226)	6.09 ± 0.67 (5.25 – 6.97)	66.5 ± 30.2 (28.6 – 104)	8,454,000
Alouatta	Mammals	Primates	982,743 ± 271,913 (722,876 – 1,259,838)	6,063,544 ± 1,795,897 (4,351,306 – 7,905,077)	6.14 ± 0.13 (5.99 – 6.3)	53.1 ± 7.76 (43.1 – 63.8)	8,039,500
Tinamus	Birds	Terrestrial birds	907,832 ± 160,366 (757,216 – 1,071,518)	1,176,052 ± 277,865 (913,448 – 1,463,593)	1.28 ± 0.08 (1.18 – 1.39)	9.36 ± 2.14 (6.62 – 12.2)	7,989,300
Mazama nemorivaga	Mammals	Ungulates	894,290 ± 184,001 (713,417 – 1,078,813)	13,861,583 ± 3,553,512 (10,399,762 – 17,492,808)	15.4 ± 0.80 (14.4 – 16.4)	0.51 ± 0.11 (0.36 – 0.65)	7,301,400
Sapajus	Mammals	Primates	854,476 ± 157,623 (709,050 – 1,016,197)	2,859,958 ± 710,893 (2,196,965 – 3,597,991)	3.32 ± 0.22 (3.04 – 3.6)	17.4 ± 2.85 (13.8 – 20.9)	7,116,300
Hydrochoerus hydrochaeris	Mammals	Large rodents	802,577 ± 244,330 (560,572 – 1,039,317)	30,599,214 ± 10,977,268 (19,815,554 – 41,539,971)	37.6 ± 2.3 (34.6 – 40.5)	2.25 ± 1.24 (0.58 – 3.89)	8,030,200
Tapirus terrestris	Mammals	Ungulates	611,000 ± 139,065 (473,884 – 750,406)	86,414,565 ± 23,906,332 (62,997,511 – 110,790,335)	140.0 ± 7.21 (131.0 – 149.0)	0.39 ± 0.07 (0.30 – 0.48)	8,012,000
Ateles	Mammals	Primates	608,524 ± 105,371 (511,322 – 716,880)	4,970,902 ± 1,055,483 (3,995,946 – 6,067,253)	8.12 ± 0.33 (7.71 – 8.56)	19.8 ± 2.94 (16.2 – 23.7)	5,534,100
Cairina moschata	Birds	Aquatic birds	593,557 ± 310,117 (281,849 – 853,726)	1,797,388 ± 1,077,143 (721,903 – 2,804,947)	2.87 ± 0.39 (2.37 – 3.37)	4.31 ± 3.13 (0.05 – 8.77)	7,754,500
Ramphastos	Birds	Ramphastids	553,329 ± 109,795 (449,848 – 664,633)	380,980 ± 98,459 (287,828 – 482,458)	0.68 ± 0.04 (0.63 – 0.73)	—	8,051,400
Nasua nasua	Mammals	Procyonids	531,135 ± 110,953 (430,055 – 645,990)	2,058,869 ± 516,882 (1,585,369 – 2,598,262)	3.85 ± 0.17 (3.64 – 4.06)	8.2 ± 2.01 (5.77 – 10.7)	7,788,200
Lagothrix	Mammals	Primates	449,370 ± 102,742 (351,327 – 553,055)	3,317,458 ± 899,247 (2,457,448 – 4,236,222)	7.32 ± 0.32 (6.9 – 7.73)	13.2 ± 2.5 (9.95 – 16.4)	2,954,600
Nannopterum brasilianus	Birds	Aquatic birds	412,301 ± 175,585 (242,781 – 574,023)	484,099 ± 237,936 (255,670 – 715,207)	1.14 ± 0.09 (1.02 – 1.27)	814.0 ± 597.0 (0.65 – 1,636)	8,045,900
Caiman crocodilus	Reptiles	Caimans	384,284 ± 105,964 (285,466 – 488,957)	6,052,857 ± 2,693,029 (3,535,733 – 8,853,701)	15.1 ± 2.83 (11.6 – 18.7)	415,546 ± 302,223 (0.67 – 816,969)	7,047,900
Crypturellus	Birds	Terrestrial birds	384,135 ± 83,691 (307,065 – 471,147)	226,453 ± 65,633 (165,605 – 295,360)	0.58 ± 0.04 (0.53 – 0.64)	22.2 ± 5.95 (14.5 – 29.6)	8,067,300
Psophia	Birds	Terrestrial birds	359,693 ± 63,527 (301,457 – 425,170)	473,657 ± 101,023 (380,557 – 578,710)	1.31 ± 0.05 (1.25 – 1.37)	13.8 ± 1.86 (11.4 – 16.2)	7,224,100
Patagioenas	Birds	Columbids	348,807 ± 85,225 (273,476 – 438,384)	82,838 ± 27,036 (58,664 – 111,454)	0.23 ± 0.02 (0.21 – 0.26)	—	8,147,400
Ardeidae (others)	Birds	Aquatic birds	327,805 ± 126,394 (211,696 – 461,454)	181,474 ± 84,831 (104,070 – 271,877)	0.54 ± 0.0478 (0.48 – 0.60)	—	8,045,900

Taxon	Class	Group	Individual animals hunted	Animal biomass extracted (kg)	Body mass (kg)	Density (ind/km^2)	Geographic distribution area (km^2)
Amazona	Birds	Large psittacids	313,159 ± 68,165 (250,715 – 383,881)	237,408 ± 65,078 (177,214 – 305,485)	0.75 ± 0.04 (0.69 – 0.80)	—	7,992,500
Podocnemis expansa	Reptiles	River turtles	310,662 ± 94,889 (222,213 – 407,136)	9,589,431 ± 4,901,554 (5,028,547 – 14,748,891)	29.4 ± 6.67 (20.8 – 37.5)	4.95 ± 2.93 (1.07 – 8.81)	7,360,200
Crax	Birds	Cracids	301,946 ± 49,574 (255,260 – 352,084)	894,008 ± 170,610 (732,945 – 1,067,964)	2.95 ± 0.08 (2.84 – 3.06)	10.4 ± 4.18 (4.81 – 15.8)	3,985,900
Podocnemis sextuberculata	Reptiles	River turtles	290,495 ± 73,091 (224,698 – 366,261)	628,120 ± 296,670 (356,174 – 943,086)	2.07 ± 0.48 (1.45 – 2.68)	174.0 ± 0 (174.0 – 174.0)	2,687,600
Anura	Anurans	Frogs	274,521 ± 86,498 (196,384 – 365,168)	209,118 ± 176,968 (50,136 – 398,614)	0.67 ± 0.39 (0.154 – 1.18)	—	8,045,900
Iguana iguana	Reptiles	Lizards	274,125 ± 73,904 (209,165 – 351,661)	545,143 ± 305,886 (272,713 – 872,218)	1.87 ± 0.59 (1.14 – 2.62)	1,073 ± 704.0 (28.6 – 2,031)	7,438,300
Ortalis	Birds	Cracids	262,046 ± 50,986 (213,724 – 314,450)	223,592 ± 71,141 (155,708 – 297,924)	0.83 ± 0.10 (0.70 – 0.97)	3.13 ± 0.52 (2.46 – 3.80)	6,462,900
Sciurus (Hadrosciurus)	Mammals	Small rodents	260,233 ± 53,595 (210,509 – 315,507)	162,302 ± 42,710 (122,688 – 206,895)	0.62 ± 0.04 (0.57 – 0.67)	9.93 ± 3.58 (5.5 – 14.5)	4,818,100
Saimiri	Mammals	Primates	259,430 ± 63,586 (202,952 – 325,313)	275,471 ± 79,866 (204,020 – 358,835)	1.05 ± 0.05 (0.99 – 1.11)	45.6 ± 8.67 (34.6 – 56.7)	6,539,700
Pipile	Birds	Cracids	245,604 ± 46,580 (202,586 – 293,699)	403,473 ± 98,198 (312,169 – 505,858)	1.63 ± 0.09 (1.51 – 1.74)	4.29 ± 1.71 (2.17 – 6.52)	6,359,000
Peltocephalus dumerilianus	Reptiles	River turtles	237,961 ± 63,041 (179,869 – 302,724)	2,674,899 ± 1,152,478 (1,614,246 – 3,892,201)	10.8 ± 1.88 (8.5 – 13.3)	383.0 ± 272.0 (9.86 – 748.0)	3,738,700
Ara	Birds	Large psittacids	212,245 ± 39,849 (175,325 – 253,094)	235,658 ± 52,132 (186,998 – 289,425)	1.10 ± 0.04 (1.05 – 1.15)	—	8,024,500
Melanosuchus niger	Reptiles	Caimans	206,608 ± 59,729 (151,676 – 267,788)	32,991,680 ± 18,835,148 (15,580,089 – 52,967,639)	149.0 ± 45.9 (89.6 – 207.0)	31.2 ± 20.5 (1.51 – 58.9)	6,037,600
Ardea alba	Birds	Aquatic birds	196,167 ± 95,539 (108,220 – 296,964)	259,452 ± 156,077 (118,962 – 426,039)	1.26 ± 0.16 (1.05 – 1.47)	47.2 ± 18.8 (24.4 – 71.4)	8,045,900
Euphractus sexcinctus	Mammals	Cingulates	194,992 ± 52,951 (143,513 – 248,701)	901,960 ± 335,721 (580,338 – 1,250,449)	4.52 ± 0.48 (3.92 – 5.16)	2.35 ± 0.79 (1.34 – 3.38)	2,103,500
Myoprocta	Mammals	Large rodents	181,713 ± 39,784 (143,635 – 219,745)	194,464 ± 52,508 (144,143 – 246,277)	1.06 ± 0.06 (0.98 – 1.14)	47.6 ± 33.0 (0.56 – 94.0)	3,772,700
Odontophorus	Birds	Terrestrial birds	162,036 ± 31,488 (132,468 – 194,186)	74,358 ± 23,958 (51,576 – 99,303)	0.45 ± 0.06 (0.37 – 0.52)	41.9 ± 8.86 (30.8 – 53.2)	7,841,700
Podocnemis erythrocephala	Reptiles	River turtles	161,338 ± 31,340 (132,847 – 193,695)	383,520 ± 257,952 (141,638 – 655,573)	2.21 ± 1.08 (0.78 – 3.63)	1.23 ± 0 (1.23 – 1.23)	1,237,600
Aotus	Mammals	Primates	159,318 ± 34,733 (126,926 – 194,709)	182,162 ± 49,022 (136,144 – 232,517)	1.13 ± 0.06 (1.05 – 1.21)	23.9 ± 2.65 (20.5 – 27.1)	6,741,900
Cebus	Mammals	Primates	157,616 ± 29,915 (130,345 – 188,579)	511,626 ± 112,484 (408,218 – 628,568)	3.23 ± 0.10 (3.10 – 3.36)	11.7 ± 1.52 (9.79 – 13.7)	3,974,600
Columbidae (others)	Birds	Columbids	151,703 ± 49,982 (104,992 – 203,683)	19,923 ± 7,925 (12,568 – 28,248)	0.13 ± 0.01 (0.12 – 0.14)	—	8,045,900
Potos flavus	Mammals	Procyonids	143,128 ± 31,084 (114,125 – 174,818)	333,022 ± 98,168 (240,354 – 434,289)	2.30 ± 0.19 (2.05 – 2.53)	51.7 ± 22.6 (22.2 – 79.9)	7,910,300
Anhinga anhinga	Birds	Aquatic birds	139,770 ± 46,418 (96,829 – 187,973)	184,283 ± 69,042 (120,697 – 256,577)	1.30 ± 0.06 (1.23 – 1.38)	7.79 ± 3.37 (3.29 – 12.1)	7,885,900
Pithecia	Mammals	Primates	137,781 ± 27,745 (112,421 – 166,531)	356,364 ± 88,894 (274,784 – 449,330)	2.57 ± 0.12 (2.40 – 2.73)	7.52 ± 0.76 (6.53 – 8.52)	3,852,300
Sylvilagus	Mammals	Lagomorphs	124,777 ± 25,088 (101,243 – 150,831)	140,352 ± 49,158 (93,493 – 191,989)	1.10 ± 0.17 (0.88 – 1.31)	631.0 ± 128.0 (464.0 – 795.0)	1,901,200
Dendrocygna autumnalis	Birds	Aquatic birds	121,305 ± 40,706 (82,702 – 162,853)	100,148 ± 37,851 (64,352 – 139,192)	0.82 ± 0.04 (0.77 – 0.86)	0.93 ± 0.46 (0.36 – 1.51)	3,999,000
Dasypus septemcinctus	Mammals	Cingulates	118,105 ± 46,518 (72,054 – 162,338)	393,017 ± 206,929 (192,375 – 602,706)	3.17 ± 0.52 (2.50 – 3.83)	0.62 ± 0.35 (0.11 – 1.08)	1,490,900
Priodontes maximus	Mammals	Cingulates	116,928 ± 52,053 (65,186 – 167,049)	3,436,496 ± 1,864,594 (1,619,842 – 5,322,134)	28.1 ± 3.46 (23.7 – 32.6)	0.33 ± 0.21 (0.04 – 0.60)	7,898,800
Anatidae (others)	Birds	Aquatic birds	116,367 ± 46,245 (72,869 – 164,938)	71,701 ± 33,607 (40,430 – 107,337)	0.60 ± 0.05 (0.54 – 0.66)	—	8,045,900
Passeriformes (others)	Birds	Small birds	115,361 ± 38,687 (81,262 – 155,702)	7,266 ± 3,096 (4,518 – 10,529)	0.061 ± 0.0058 (0.054 – 0.069)	—	8,045,900
Paleosuchus trigonatus	Reptiles	Caimans	110,560 ± 48,612 (62,740 – 153,998)	909,335 ± 487,104 (435,248 – 1,384,272)	7.87 ± 1.02 (6.58 – 9.25)	1.46 ± 0.97 (0.03 – 2.77)	6,619,400

Taxon	Class	Group	Individual animals hunted	Animal biomass extracted (kg)	Body mass (kg)	Density (ind/km^2)	Geographic distribution area (km^2)
Leopardus pardalis	Mammals	Felids	100,942 ± 22,764 (79,515 – 124,442)	990,524 ± 273,739 (733,212 – 1,276,036)	9.71 ± 0.50 (9.11 – 10.4)	0.33 ± 0.06 (0.26 – 0.40)	8,057,200
Psittacidae (others)	Birds	Small birds	94,675 ± 24,122 (72,636 – 119,734)	14,509 ± 4,939 (9,972 – 19,695)	0.15 ± 0.01 (0.13 – 0.16)	—	8,045,900
Coendou	Mammals	Large rodents	93,424 ± 23,161 (71,660 – 117,428)	369,638 ± 116,362 (260,524 – 491,738)	3.90 ± 0.27 (3.57 – 4.25)	31.5 ± 8.33 (20.8 – 42.2)	8,093,000
Echimyidae	Mammals	Small rodents	91,219 ± 35,857 (58,304 – 128,635)	39,661 ± 19,205 (22,280 – 59,989)	0.42 ± 0.04 (0.37 – 0.47)	—	8,045,900
Dasypus sabanicola	Mammals	Cingulates	89,235 ± 15,820 (74,915 – 105,476)	139,081 ± 68,991 (74,485 – 211,649)	1.49 ± 0.49 (0.87 – 2.14)	28.0 ± 28.0 (28.0 – 28.0)	87
Didelphis	Mammals	Marsupials	87,978 ± 19,143 (70,465 – 107,743)	119,622 ± 42,580 (80,117 – 164,349)	1.33 ± 0.19 (1.09 – 1.57)	46.5 ± 15.8 (26.6 – 66.3)	7,797,800
Ardea cocoi	Birds	Aquatic birds	85,860 ± 38,215 (51,898 – 126,420)	202,422 ± 106,830 (108,195 – 316,633)	2.29 ± 0.12 (2.03 – 2.55)	3.39 ± 1.13 (1.99 – 4.8)	8,033,700
Dasypus kappleri	Mammals	Cingulates	85,779 ± 29,652 (57,961 – 115,554)	708,794 ± 296,097 (433,165 – 1,013,916)	8.10 ± 0.63 (7.30 – 8.92)	2.23 ± 1.57 (0.11 – 4.37)	6,720,300
Tamandua tetradactyla	Mammals	Myrmecophagids	84,093 ± 21,802 (64,259 – 106,901)	416,406 ± 131,083 (296,734 – 554,636)	4.89 ± 0.28 (4.53 – 5.25)	4.33 ± 2.60 (0.83 – 7.78)	7,899,900
Plecturocebus	Mammals	Primates	79,897 ± 17,937 (63,477 – 98,586)	98,491 ± 30,057 (70,912 – 130,189)	1.22 ± 0.09 (1.09 – 1.35)	54.0 ± 15.4 (34.5 – 73.8)	3,841,500
Puma concolor	Mammals	Felids	62,249 ± 12,050 (50,740 – 74,581)	2,484,213 ± 772,816 (1,744,325 – 3,289,878)	39.2 ± 4.77 (33.0 – 45.1)	0.05 ± 0.01 (0.04 – 0.06)	8,135,900
Paleosuchus palpebrosus	Reptiles	Caimans	61,395 ± 17,118 (45,716 – 79,255)	167,806 ± 77,559 (97,094 – 250,348)	2.62 ± 0.49 (1.99 – 3.26)	7.26 ± 3.73 (2.16 – 12.2)	7,568,900
Chiropotes	Mammals	Primates	55,776 ± 15,341 (41,926 – 71,844)	159,613 ± 54,288 (110,632 – 217,050)	2.82 ± 0.19 (2.58 – 3.06)	7.85 ± 1.89 (5.29 – 10.4)	3,376,200
Cabassous unicinctus	Mammals	Cingulates	54,581 ± 17,138 (38,696 – 72,473)	261,517 ± 125,753 (144,586 – 394,511)	4.60 ± 0.82 (3.47 – 5.59)	0.74 ± 0.23 (0.45 – 1.03)	7,574,700
Panthera onca	Mammals	Felids	54,575 ± 12,285 (42,683 – 67,139)	3,887,301 ± 1,120,780 (2,805,364 – 5,047,594)	70.5 ± 4.59 (64.4 – 76.1)	0.02 ± 0.005 (0.018 – 0.03)	7,378,600
Opisthocomus hoazin	Birds	Aquatic birds	53,664 ± 21,903 (33,136 – 75,858)	41,636 ± 18,537 (24,337 – 60,636)	0.77 ± 0.03 (0.72 – 0.81)	2.73 ± 1.97 (0.24 – 5.35)	7,744,800
Myrmecophaga tridactyla	Mammals	Myrmecophagids	51,519 ± 11,324 (41,084 – 63,267)	1,546,487 ± 413,005 (1,162,511 – 1,977,768)	29.8 ± 1.46 (27.8 – 31.5)	0.72 ± 0.19 (0.48 – 0.96)	7,681,900
Cavia	Mammals	Small rodents	51,170 ± 22,722 (29,821 – 73,471)	31,334 ± 15,808 (16,587 – 47,319)	0.59 ± 0.04 (0.54 – 0.65)	67.4 ± 47.0 (0.38 – 128)	1,581,200
Eira barbara	Mammals	Mustelids	46,680 ± 12,555 (35,044 – 59,746)	214,180 ± 78,671 (140,975 – 297,024)	4.49 ± 0.45 (3.90 – 5.06)	0.38 ± 0.06 (0.30 – 0.46)	7,750,900
Odocoileus virginianus	Mammals	Ungulates	46,096 ± 10,354 (36,610 – 56,941)	1,790,394 ± 524,388 (1,305,539 – 2,343,028)	38.4 ± 2.69 (34.9 – 41.7)	3.79 ± 0.65 (2.92 – 4.65)	1,560,400
Cheracebus	Mammals	Primates	44,929 ± 10,640 (35,250 – 56,014)	73,686 ± 24,375 (51,312 – 99,373)	1.61 ± 0.156 (1.42 – 1.81)	2.88 ± 0.66 (2.05 – 3.76)	2,071,900
Cacajao	Mammals	Primates	42,382 ± 10,524 (32,900 – 53,355)	138,601 ± 43,711 (98,861 – 184,621)	3.23 ± 0.22 (2.95 – 3.51)	17.3 ± 4.61 (11.5 – 23.4)	891,6
Pteroglossus	Birds	Ramphastids	39,395 ± 11,443 (28,775 – 51,349)	8,263 ± 3,271 (5,247 – 11,723)	0.20 ± 0.02 (0.18 – 0.23)	7.4 ± 2.19 (4.57 – 10.3)	7,966,800
Tupinambis teguixin	Reptiles	Lizards	38,460 ± 13,782 (26,081 – 52,967)	63,026 ± 43,568 (24,379 – 109,880)	1.49 ± 0.56 (0.80 – 2.18)	6.30 ± 0 (6.30 – 6.30)	7,813,100
Leontocebus	Mammals	Primates	37,264 ± 10,309 (27,823 – 48,085)	18,784 ± 7,295 (12,107 – 26,526)	0.49 ± 0.06 (0.42 – 0.56)	23.6 ± 2.03 (21 – 26.1)	2,131,100
Pionus	Birds	Small birds	36,980 ± 7,165 (30,290 – 44,331)	8,652 ± 2,392 (6,394 – 11,140)	0.23 ± 0.02 (0.21 – 0.26)	1.73 ± 0.21 (1.45 – 2.01)	2,582,400
Bradypus	Mammals	Folivores	36,194 ± 9,519 (27,655 – 46,272)	175,102 ± 60,259 (120,272 – 239,237)	4.76 ± 0.40 (4.24 – 5.25)	262.0 ± 113.0 (113.0 – 408.0)	7,469,200
Podocnemis vogli	Reptiles	River turtles	30,742 ± 5,801 (25,566 – 36,775)	61,484 ± 11,601 (51,132 – 73,550)	2.0 ± 0 (2.0 – 2.0)	85.0 ± 0 (85.0 – 85.0)	1,493,900
Psarocolius	Birds	Small birds	29,985 ± 6,316 (24,141 – 36,504)	8,942 ± 2,431 (6,683 – 11,475)	0.29 ± 0.02 (0.27 – 0.32)	—	8,028,400
Choloepus	Mammals	Folivores	29,184 ± 10,809 (19,376 – 40,532)	185,168 ± 81,333 (111,501 – 271,236)	6.21 ± 0.45 (5.62 – 6.78)	30.8 ± 13.5 (13.6 – 47.9)	5,671,100
Galea	Mammals	Large rodents	27,695 ± 9,885 (18,136 – 37,771)	7,634 ± 3,648 (4,190 – 11,449)	0.26 ± 0.03 (0.22 – 0.31)	322.0 ± 0 (322.0 – 322.0)	471,400

Taxon	Class	Group	Individual animals hunted	Animal biomass extracted (kg)	Body mass (kg)	Density (ind/km^2)	Geographic distribution area (km^2)
Mesembrinibis cayennensis	Birds	Aquatic birds	27,319 ± 11,147 (16,815 – 38,812)	23,483 ± 11,750 (12,561 – 35,811)	0.83 ± 0.08 (0.72 – 0.94)	0.22 ± 0.10 (0.08 – 0.34)	7,660,200
Dinomys branickii	Mammals	Large rodents	26,638 ± 7,070 (20,127 – 34,091)	248,738 ± 90,463 (165,248 – 344,855)	9.14 ± 0.92 (7.96 – 10.3)	20.0 ± 7.63 (10.3 – 29.9)	1,241,700
Scolopacidae	Birds	Aquatic birds	26,483 ± 12,121 (15,669 – 39,397)	8,957 ± 4,839 (4,664 – 14,136)	0.33 ± 0.03 (0.29 – 0.36)	—	8,045,900
Nothocrax urumutum	Birds	Cracids	23,586 ± 5,266 (18,768 – 29,062)	39,575 ± 13,418 (27,114 – 53,700)	1.65 ± 0.193 (1.39 – 1.89)	5.01 ± 0.80 (4.03 – 5.99)	2,341,300
Ameiva ameiva	Reptiles	Lizards	22,044 ± 8,541 (14,567 – 31,055)	846 ± 782 (153 – 1,681)	0.033 ± 0.019 (0.004 – 0.058)	144.0 ± 74.5 (46.5 – 240.0)	7,438,300
Cotingidae	Birds	Small birds	21,547 ± 7,647 (14,709 – 29,535)	2,696 ± 1,226 (1,603 – 3,994)	0.122 ± 0.012 (0.106 – 0.138)	—	8,045,900
Selenidera	Birds	Ramphastids	21,415 ± 11,091 (11,566 – 33,029)	5,382 ± 3,847 (2,035 – 9,478)	0.23 ± 0.05 (0.16 – 0.29)	—	6,514,400
Mesoclemmys	Reptiles	River turtles	17,707 ± 5,276 (12,898 – 23,216)	41,340 ± 14,672 (27,989 – 56,797)	2.30 ± 0.13 (2.13 – 2.48)	156.0 ± 0 (156.0 – 156.0)	7,438,300
Leopardus wiedii	Mammals	Felids	16,176 ± 4,951 (11,596 – 21,352)	115,277 ± 55,972 (63,905 – 174,903)	6.80 ± 1.30 (5.20 – 8.46)	0.13 ± 0.04 (0.08 – 0.18)	7,781,000
Lontra longicaudis	Mammals	Mustelids	15,345 ± 5,287 (10,399 – 20,786)	127,556 ± 68,576 (64,282 – 200,151)	7.86 ± 1.65 (5.74 – 9.97)	0.31 ± 0.15 (0.10 – 0.50)	7,973,700
Saguinus	Mammals	Primates	15,083 ± 3,776 (11,667 – 19,025)	7,381 ± 2,157 (5,424 – 9,641)	0.486 ± 0.0219 (0.457 – 0.513)	18.2 ± 2.95 (14.5 – 22)	3,424,800
Mazama gouazoubira	Mammals	Ungulates	14,599 ± 2,935 (11,806 – 17,587)	247,204 ± 75,042 (175,232 – 325,043)	16.6 ± 1.79 (14.4 – 18.9)	4.40 ± 1.00 (3.10 – 5.66)	395,500
Accipitridae	Birds	Raptor birds	13,755 ± 4,144 (9,974 – 18,086)	8,775 ± 3,317 (5,742 – 12,271)	0.62 ± 0.050 (0.56 – 0.69)	—	8,045,900
Aramidae	Birds	Aquatic birds	13,532 ± 5,865 (8,153 – 19,709)	13,093 ± 6,611 (7,064 – 20,107)	0.94 ± 0.0 (0.84 – 1.03)	—	8,045,900
Chamaepetes goudotii	Birds	Cracids	12,984 ± 6,168 (6,978 – 16,806)	9,283 ± 5,078 (4,054 – 13,473)	0.68 ± 0.12 (0.52 – 0.84)	12.9 ± 1.31 (11.3 – 14.6)	127,100
Trichechus inunguis	Mammals	Sirenians	12,827 ± 3,501 (9,570 – 16,456)	4,166,916 ± 2,001,413 (2,318,098 – 6,291,464)	310.0 ± 67.5 (224.0 – 398.0)	0.16 ± 0.05 (0.10 – 0.23)	746,700
Herpailurus yagouaroundi	Mammals	Felids	11,878 ± 3,425 (8,654 – 15,376)	64,946 ± 28,638 (38,327 – 95,006)	5.27 ± 0.85 (4.18 – 6.39)	0.22 ± 0.11 (0.07 – 0.37)	7,801,700
Tolypeutes tricinctus	Mammals	Cingulates	11,138 ± 3,227 (8,059 – 14,467)	15,810 ± 6,635 (9,519 – 22,782)	1.38 ± 0.19 (1.13 – 1.61)	1.20 ± 0 (1.20 – 1.20)	1,235,400
Neochen jubata	Birds	Aquatic birds	10,895 ± 2,648 (8,401 – 13,596)	17,620 ± 6,521 (11,464 – 24,405)	1.58 ± 0.21 (1.31 – 1.84)	3.9 ± 0.66 (3.05 – 4.72)	7,453,300
Falconidae	Birds	Raptor birds	10,876 ± 3,028 (8,020 – 13,958)	8,569 ± 2,943 (5,811 – 11,615)	0.78 ± 0.05 (0.71 – 0.84)	—	8,045,900
Chelus fimbriata	Reptiles	River turtles	10,097 ± 3,501 (6,961 – 13,744)	96,541 ± 62,016 (41,109 – 162,518)	8.80 ± 2.85 (5.11 – 12.4)	41.6 ± 0 (41.6 – 41.6)	8,743,100
Picidae	Birds	Small birds	10,057 ± 3,207 (7,078 – 13,360)	2,083 ± 807 (1,337 – 2,925)	0.20 ± 0.01 (0.18 – 0.22)	—	8,045,900
Spatula discors	Birds	Aquatic birds	9,753 ± 859 (8,970 – 10,617)	3,877 ± 410 (3,505 – 4,290)	0.40 ± 0.01 (0.38 – 0.41)	4.50 ± 1.39 (2.67 – 6.28)	649,300
Platemys platycephala	Reptiles	River turtles	9,661 ± 2,959 (6,967 – 12,755)	19,448 ± 10,707 (9,685 – 30,857)	1.90 ± 0.50 (1.26 – 2.52)	189.0 ± 0 (189.0 – 189.0)	7,311,900
Eunectes murinus	Reptiles	Snakes	9,024 ± 3,020 (6,269 – 12,196)	666,721 ± 273,741 (418,314 – 956,928)	72.3 ± 5.69 (65.2 – 79.8)	3.6 0± 0 (3.60 – 3.60)	7,438,300
Rhinemys rufipes	Reptiles	River turtles	9,004 ± 2,026 (7,192 – 11,127)	5,403 ± 1,215 (4,315 – 6,676)	0.6 ± 0 (0.6 – 0.6)	37.8 ± 2.79 (34.4 – 41.5)	1,016,500
Eudocimus ruber	Birds	Aquatic birds	8,825 ± 2,876 (6,302 – 11,908)	6,760 ± 3,225 (3,877 – 10,218)	0.74 ± 0.12 (0.58 – 0.88)	4.41 ± 0 (4.41 – 4.41)	302,200
Trogonidae	Birds	Small birds	8,330 ± 3,086 (5,452 – 11,565)	789 ± 348 (467 – 1,158)	0.093 ± 0.007 (0.084 – 0.102)	—	8,045,900
Cerdocyon thous	Mammals	Canids	8,227 ± 2,476 (5,971 – 10,838)	50,115 ± 20,672 (31,340 – 72,149)	5.92 ± 0.68 (5.08 – 6.81)	0.61 ± 0.11 (0.47 – 0.76)	1,898,900
Colinus cristatus	Birds	Terrestrial birds	8,136 ± 2,615 (5,836 – 10,895)	1,144 ± 408 (784 – 1,576)	0.139 ± 0.005 (0.133 – 0.146)	81.6 ± 25.4 (49.6 – 113)	974,200
Harpia harpyja	Birds	Raptor birds	7,696 ± 2,479 (5,439 – 10,296)	47,338 ± 22,974 (26,376 – 71,714)	5.89 ± 1.01 (4.55 – 7.18)	0.50 ± 0.08 (0.39 – 0.61)	7,588,700

Taxon	Class	Group	Individual animals hunted	Animal biomass extracted (kg)	Body mass (kg)	Density (ind/km^2)	Geographic distribution area (km^2)
Serpentes (others)	Reptiles	Snakes	6,795 ± 2,696 (4,365 – 9,621)	31,811 ± 33,381 (3,165 – 67,725)	3.80 ± 2.76 (0 – 7.75)	—	8,045,900
Cuculidae	Birds	Small birds	6,504 ± 2,068 (4,576 – 8,633)	1,120 ± 479 (675 – 1,621)	0.167 ± 0.019 (0.142 – 0.192)	—	8,045,900
Pteronura brasiliensis	Mammals	Mustelids	6,269 ± 2,122 (4,343 – 8,495)	159,612 ± 72,347 (94,202 – 236,398)	24.7 ± 2.88 (20.9 – 28.5)	0.27 ± 0.05 (0.20 – 0.34)	6,792,900
Heliornis fulica	Birds	Aquatic birds	6,117 ± 2,310 (4,059 – 8,548)	853 ± 384 (513 – 1,261)	0.14 ± 0.01 (0.123 – 0.15)	4.66 ± 2.21 (1.77 – 7.55)	7,725,800
Boa constrictor	Reptiles	Snakes	5,823 ± 2,270 (3,795 – 8,221)	81,862 ± 50,461 (37,344 – 135,932)	13.1 ± 3.24 (8.97 – 17.2)	49.4 ± 26.5 (12.9 – 84.1)	7,438,300
Burhinus bistriatus	Birds	Aquatic birds	5,728 ± 1,123 (4,727 – 6,899)	4,121 ± 901 (3,312 – 5,063)	0.72 ± 0.02 (0.69 – 0.74)	4.50 ± 0 (4.50 – 4.50)	154,400
Cricetidae	Mammals	Small rodents	5,388 ± 2,675 (3,032 – 8,210)	1,178 ± 777 (501 – 2,007)	0.20 ± 0.04 (0.15 – 0.25)	—	8,045,900
Sciurus (Notosciurus)	Mammals	Small rodents	5,133 ± 1,653 (3,603 – 6,857)	1,091 ± 408 (715 – 1,520)	0.21 ± 0.01 (0.19 – 0.22)	10.4 ± 3.58 (5.72 – 14.9)	8,045,900
Speothos venaticus	Mammals	Canids	5,005 ± 1,938 (3,174 – 6,989)	38,195 ± 18,173 (21,221 – 57,164)	7.41 ± 0.71 (6.49 – 8.32)	0.29 ± 0.22 (0.04 – 0.59)	7,856,200
Momotidae	Birds	Small birds	4,616 ± 1,693 (3,037 – 6,364)	536 ± 248 (308 – 796)	0.11 ± 0.01 (0.09 – 0.13)	—	8,045,900
Blastocerus dichotomus	Mammals	Ungulates	4,064 ± 827 (3,318 – 4,922)	332,037 ± 133,530 (208,001 – 472,833)	79.4 ± 16.3 (58.3 – 99.8)	0.49 ± 0.08 (0.39 – 0.60)	88,100
Ciconia maguari	Birds	Aquatic birds	3,784 ± 1,021 (2,801 – 4,813)	14,846 ± 5,086 (9,970 – 20,079)	3.86 ± 0.30 (3.48 – 4.24)	0.02 ± 0 (0.02 – 0.02)	1,500,300
Anodorhynchus hyacinthinus	Birds	Large psittacids	3,772 ± 1,439 (2,479 – 5,299)	5,854 ± 2,434 (3,668 – 8,445)	1.53 ± 0.06 (1.46 – 1.6)	43.8 ± 18.6 (20 – 67.8)	1,492,800
Mycteria americana	Birds	Aquatic birds	3,639 ± 1,162 (2,608 – 4,857)	9,950 ± 3,885 (6,484 – 14,055)	2.69 ± 0.19 (2.43 – 2.94)	7.75 ± 3.52 (3.05 – 12.2)	7,811,900
Rhinoclemmys punctularia	Reptiles	River turtles	3,605 ± 1,222 (2,494 – 4,893)	8,007 ± 3,312 (5,002 – 11,525)	2.17 ± 0.17 (1.97 – 2.39)	165.0 ± 0 (165.0 – 165.0)	1,607,800
Ozotoceros bezoarticus	Mammals	Ungulates	3,596 ± 3,292 (0 – 6,155)	132,605 ± 121,840 (0 – 230,702)	35.1 ± 2.76 (31.4 – 38.5)	2.72 ± 0.89 (1.59 – 3.87)	192,600
Jacana jacana	Birds	Aquatic birds	3,527 ± 1,686 (2,050 – 5,314)	509 ± 320 (232 – 851)	0.14 ± 0.02 (0.11 – 0.16)	0.97 ± 0.45 (0.38 – 1.58)	7,901,000
Cyclopes didactylus	Mammals	Myrmecophagids	3,314 ± 1,164 (2,254 – 4,536)	1,558 ± 888 (757 – 2,507)	0.44 ± 0.11 (0.31 – 0.57)	31 ± 9.56 (18.9 – 43.4)	7,436,300
Sarcoramphus papa	Birds	Raptor birds	3,304 ± 1,504 (1,930 – 4,872)	11,664 ± 6,185 (6,088 – 18,185)	3.42 ± 0.28 (3.08 – 3.79)	0.01 ± 0.004 (0.01 – 0.02)	7,932,900
Strigidae	Birds	Raptor birds	3,286 ± 1,321 (2,088 – 4,683)	1,447 ± 744 (778 – 2,241)	0.42 ± 0.051 (0.36 – 0.49)	—	8,045,900
Jabiru mycteria	Birds	Aquatic birds	2,976 ± 1,221 (1,883 – 4,266)	21,179 ± 10,286 (12,004 – 32,121)	6.94 ± 0.54 (6.23 – 7.64)	0.09 ± 0 (0.09 – 0.09)	5,043,200
Theristicus caudatus	Birds	Aquatic birds	2,912 ± 1,114 (1,923 – 4,099)	4,359 ± 2,420 (2,225 – 6,961)	1.42 ± 0.26 (1.09 – 1.75)	5.8 ± 4.28 (0.19 – 11.7)	809,200
Bucconidae	Birds	Small birds	2,893 ± 1,058 (1,941 – 4,014)	253 ± 137 (131 – 400)	0.083 ± 0.0153 (0.063 – 0.103)	—	8,045,900
Kinosternon scorpioides	Reptiles	River turtles	2,878 ± 809 (2,174 – 3,709)	1,611 ± 782 (905 – 2,425)	0.54 ± 0.12 (0.38 – 0.68)	13,106 ± 5,078 (6,822 – 19,642)	7,004,300
Phrynops	Reptiles	River turtles	2,432 ± 1,148 (1,409 – 3,649)	4,633 ± 2,976 (2,017 – 7,825)	1.78 ± 0.35 (1.34 – 2.21)	11,024 ± 8,781 (1 – 22,935)	8,045,900
Inia geoffrensis	Mammals	Cetaceans	2,392 ± 757 (1,701 – 3,184)	247,322 ± 116,240 (141,700 – 371,027)	99.3 ± 16.1 (79.0 – 120.0)	3.14 ± 0 (3.14 – 3.14)	676,400
Chiroptera	Mammals	Bats	2,230 ± 921 (1,418 – 3,197)	79 ± 49 (36 – 131)	0.033 ± 0.008 (0.024 – 0.042)	—	8,045,900
Cathartidae (others)	Birds	Raptor birds	2,175 ± 1,202 (1,115 – 3,458)	3,778 ± 2,310 (1,759 – 6,257)	1.69 ± 0.11 (1.54 – 1.83)	—	8,045,900
Lacertilia (others)	Reptiles	Lizards	2,111 ± 849 (1,376 – 3,012)	1,055 ± 425 (688 – 1,506)	0.5 ± 0 (0.5 – 0.5)	—	7,438,300
Trochilidae	Birds	Small birds	2,038 ± 820 (1,295 – 2,892)	15 ± 7 (9 – 23)	0.007 ± 0.0005 (0.007 – 0.008)	—	8,045,900
Alcedinidae	Birds	Aquatic birds	1,961 ± 948 (1,113 – 2,958)	283 ± 212 (98 – 510)	0.13 ± 0.04 (0.08 – 0.18)	—	8,045,900

Taxon	Class	Group	Individual animals hunted	Animal biomass extracted (kg)	Body mass (kg)	Density (ind/km^2)	Geographic distribution area (km^2)
Caiman yacare	Reptiles	Caimans	1,708 ± 493 (1,258 – 2,223)	46,811 ± 32,598 (17,120 – 81,521)	25.0 ± 11.1 (10.8 – 39.1)	45.6 ± 27.5 (6.99 – 82.6)	758,700
Cariama cristata	Birds	Terrestrial birds	1,589 ± 392 (1,236 – 2,001)	2,866 ± 992 (1,964 – 3,921)	1.77 ± 0.18 (1.54 – 2.0)	191.0 ± 0 (191.0 – 191.0)	287,100
Dracaena guianensis	Reptiles	Lizards	1,472 ± 695 (869 – 2,217)	4,738 ± 3,508 (1,726 – 8,531)	2.9 ± 0.89 (1.76 – 4.02)	4.34 ± 0 (4.34 – 4.34)	2,776,500
Callimico goeldii	Mammals	Primates	1,463 ± 497 (1,039 – 1,989)	716 ± 342 (421 – 1,083)	0.47 ± 0.07 (0.39 – 0.56)	43.8 ± 22.0 (14.1 – 71.9)	704,600
Procyon cancrivorus	Mammals	Procyonids	1,329 ± 398 (964 – 1,745)	8,816 ± 4,284 (4,901 – 13,366)	6.35 ± 1.26 (4.69 – 7.97)	2.86 ± 1.37 (1.04 – 4.66)	7,463,200
Nasuella olivacea	Mammals	Procyonids	1,320 ± 73 (1,253 – 1,392)	1,680 ± 234 (1,456 – 1,903)	1.27 ± 0.15 (1.09 – 1.47)	18.1 ± 0 (18.1 – 18.1)	19,900
Bassaricyon	Mammals	Procyonids	1,067 ± 376 (722 – 1,459)	1,258 ± 549 (758 – 1,837)	1.15 ± 0.10 (1.02 – 1.28)	20.4 ± 20.4 (20.4 – 20.4)	4,946,300
Sotalia fluviatilis	Mammals	Cetaceans	1,021 ± 296 (752 – 1,329)	50,636 ± 20,997 (31,636 – 72,942)	48.1 ± 6.33 (40.3 – 56.3)	3.93 ± 1.05 (2.61 – 5.19)	900,200
Didelphidae (others)	Mammals	Marsupials	999 ± 379 (646 – 1,390)	421 ± 231 (209 – 665)	0.40 ± 0.08 (0.30 – 0.49)	—	8,045,900
Eurypyga helias	Birds	Aquatic birds	976 ± 417 (584 – 1,392)	218 ± 107 (119 – 327)	0.22 ± 0.02 (0.20 – 0.24)	0.27 ± 0.22 (0.01 – 0.57)	7,980,000
Mico	Mammals	Primates	731 ± 185 (560 – 922)	243 ± 107 (143 – 356)	0.32 ± 0.06 (0.24 – 0.40)	14.0 ± 3.83 (9.30 – 19.0)	1,188,000
Microsciurus flaviventer	Mammals	Small rodents	676 ± 230 (470 – 916)	125 ± 89 (46 – 219)	0.17 ± 0.07 (0.08 – 0.25)	8.56 ± 6.22 (0.30 – 17.1)	2,990,200
Cebuella	Mammals	Primates	628 ± 209 (435 – 847)	263 ± 171 (108 – 446)	0.39 ± 0.14 (0.21 – 0.56)	57.8 ± 26.0 (24.3 – 92.6)	1,963,400
Anhima cornuta	Birds	Aquatic birds	400 ± 171 (250 – 581)	1,229 ± 558 (742 – 1,820)	3.05 ± 0.08 (2.94 – 3.15)	2.64 ± 1.86 (0.03 – 5.09)	7,803,000
Rhea americana	Birds	Terrestrial birds	313 ± 83 (236 – 399)	8,272 ± 3,971 (4,580 – 12,458)	25.3 ± 5.67 (17.9 – 32.4)	0.37 ± 0.08 (0.27 – 0.47)	531,400
Threskiornithidae (others)	Birds	Aquatic birds	296 ± 147 (165 – 452)	444 ± 221 (248 – 678)	1.5 ± 0 (1.5 – 1.5)	—	8,045,900
Tremarctos ornatus	Mammals	Ursid	187 ± 38 (152 – 226)	22,041 ± 10,449 (12,189 – 33,133)	113.0 ± 32.7 (71.6 – 154.0)	0.07 ± 0.01 (0.05 – 0.08)	124,800
Chauna torquata	Birds	Aquatic birds	173 ± 41 (134 – 215)	619 ± 270 (364 – 903)	3.44 ± 0.70 (2.54 – 4.36)	0.07 ± 0 (0.07 – 0.07)	725,600
Atelocynus microtis	Mammals	Canids	92 ± 55 (44 – 150)	746 ± 494 (320 – 1,272)	7.87 ± 0.56 (7.15 – 8.58)	5.94 ± 0 (5.93 – 5.94)	3,347,600
Phoenicoparrus	Birds	Aquatic birds	41 ± 23 (22 – 66)	132 ± 101 (47 – 240)	2.85 ± 0.73 (1.96 – 3.81)	1.57 ± 0 (1.57 – 1.57)	8,045,900
Galictis vittata	Mammals	Mustelids	40 ± 23 (20 – 64)	102 ± 72 (41 – 180)	2.38 ± 0.39 (1.89 – 2.88)	1.68 ± 0.56 (0.97 – 2.45)	7,658,700

Supplementary Data 3. Estimated body mass for the 174 hunted taxa recorded in this study.

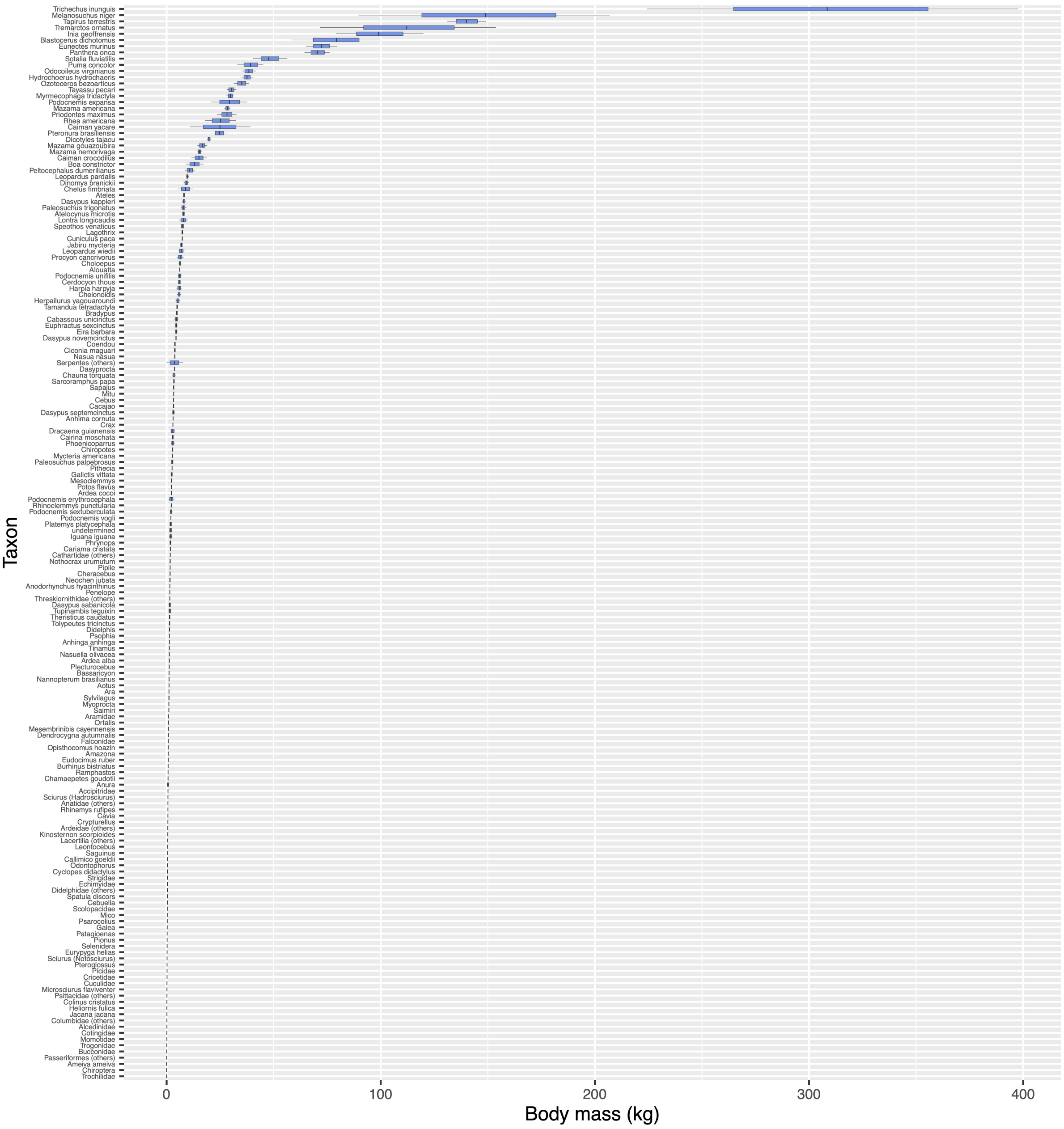

Supplementary Table 3. List of the 63 species pertaining to the 20 key dominant hunted taxa in Amazonia classified into one of nine IUCN Red List of Threatened Species Categories. Extinct (EX), Extinct in the Wild (EW), Critically Endangered (CR), Endangered (EN), Vulnerable (VU), Near Threatened (NT), Least Concern (LC), Data Deficient (DD) and Not Evaluated.

#	Species	Family	Order	Class	IUCN extinction category
1	Nasua nasua	Procyonidae	Carnivora	Mammals	LC
2	Mazama americana	Cervidae	Cetartiodactyla	Mammals	DD
3	Mazama nemorivaga	Cervidae	Cetartiodactyla	Mammals	LC
4	Dicotyles tajacu	Tayassuidae	Cetartiodactyla	Mammals	LC
5	Tayassu pecari	Tayassuidae	Cetartiodactyla	Mammals	VU
6	Dasypus novemcinctus	Dasypodidae	Cingulata	Mammals	LC
7	Tapirus terrestris	Tapiridae	Perissodactyla	Mammals	VU
8	Alouatta arctoidea	Atelidae	Primates	Mammals	LC
9	Alouatta belzebul	Atelidae	Primates	Mammals	VU
10	Alouatta caraya	Atelidae	Primates	Mammals	NT
11	Alouatta discolor	Atelidae	Primates	Mammals	VU
12	Alouatta juara	Atelidae	Primates	Mammals	LC
13	Alouatta macconnelli	Atelidae	Primates	Mammals	LC
14	Alouatta nigerrima	Atelidae	Primates	Mammals	LC
15	Alouatta puruensis	Atelidae	Primates	Mammals	VU
16	Alouatta sara	Atelidae	Primates	Mammals	NT
17	Alouatta seniculus	Atelidae	Primates	Mammals	LC
18	Alouatta ululata	Atelidae	Primates	Mammals	EN
19	Ateles belzebuth	Atelidae	Primates	Mammals	EN
20	Ateles chamek	Atelidae	Primates	Mammals	EN
21	Ateles marginatus	Atelidae	Primates	Mammals	EN
22	Ateles paniscus	Atelidae	Primates	Mammals	VU
23	Sapajus apella apella	Cebidae	Primates	Mammals	LC
24	Sapajus cay	Cebidae	Primates	Mammals	LC
25	Sapajus libidinosus	Cebidae	Primates	Mammals	NT
26	Cuniculus paca	Cuniculidae	Rodentia	Mammals	LC
27	Dasyprocta azarae	Dasyproctidae	Rodentia	Mammals	DD
28	Dasyprocta croconota	Dasyproctidae	Rodentia	Mammals	DD
29	Dasyprocta fuliginosa	Dasyproctidae	Rodentia	Mammals	LC
30	Dasyprocta guamara	Dasyproctidae	Rodentia	Mammals	NT
31	Dasyprocta kalinowskii	Dasyproctidae	Rodentia	Mammals	DD
32	Dasyprocta leporina	Dasyproctidae	Rodentia	Mammals	LC
33	Dasyprocta prymnolopha	Dasyproctidae	Rodentia	Mammals	LC
34	Dasyprocta punctata	Dasyproctidae	Rodentia	Mammals	LC
35	Dasyprocta variegata	Dasyproctidae	Rodentia	Mammals	DD
36	Hydrochoerus hydrochaeris	Caviidae	Rodentia	Mammals	LC
37	Cairina moschata	Anatidae	Anseriformes	Birds	LC

#	Species	Family	Order	Class	IUCN extinction category
38	Mitu salvini	Cracidae	Galliformes	Birds	LC
39	Mitu tomentosum	Cracidae	Galliformes	Birds	LC
40	Mitu tuberosum	Cracidae	Galliformes	Birds	NT
41	Penelope barbata	Cracidae	Galliformes	Birds	NT
42	Penelope jacquacu	Cracidae	Galliformes	Birds	LC
43	Penelope marail	Cracidae	Galliformes	Birds	LC
44	Penelope montagnii	Cracidae	Galliformes	Birds	LC
45	Penelope obscura	Cracidae	Galliformes	Birds	LC
46	Penelope ochrogaster	Cracidae	Galliformes	Birds	VU
47	Penelope pileata	Cracidae	Galliformes	Birds	VU
48	Penelope purpurascens	Cracidae	Galliformes	Birds	NT
49	Penelope superciliaris	Cracidae	Galliformes	Birds	NT
50	Ramphastos ambiguus	Ramphastidae	Piciformes	Birds	LC
51	Ramphastos ariel	Ramphastidae	Piciformes	Birds	NT
52	Ramphastos culminatus	Ramphastidae	Piciformes	Birds	LC
53	Ramphastos cuvieri	Ramphastidae	Piciformes	Birds	LC
54	Ramphastos toco	Ramphastidae	Piciformes	Birds	LC
55	Ramphastos tucanus	Ramphastidae	Piciformes	Birds	LC
56	Ramphastos vitellinus	Ramphastidae	Piciformes	Birds	LC
57	Tinamus guttatus	Tinamidae	Struthioniformes	Birds	NT
58	Tinamus major	Tinamidae	Struthioniformes	Birds	LC
59	Tinamus osgoodi	Tinamidae	Struthioniformes	Birds	VU
60	Tinamus tao	Tinamidae	Struthioniformes	Birds	VU
61	Podocnemis unifilis	Podocnemididae	Testudines	Reptiles	VU
62	Chelonoidis denticulata	Testudinidae	Testudines	Reptiles	VU
63	Chelonoidis carbonaria	Testudinidae	Testudines	Reptiles	NE

Supplementary Table 4. Estimated number of animals hunted (Individual Animals Offtake) for the 30 most hunted taxa and its proportion in relation to the total offtake in each Amazonian region.

Guiana Shield (GS)		
Taxon	Annual Individual Animals Offtake	Individual Animals Offtake proportion
Cuniculus paca	903,532	0.131
Tayassu pecari	705,068	0.103
Dicotyles tajacu	497,182	0.072
Dasyprocta	490,863	0.071
Chelonoidis	326,370	0.047
Penelope	249,223	0.036
Dasypus novemcinctus	232,404	0.034
Mazama americana	197,335	0.029
Crax	178,650	0.026
Podocnemis unifilis	170,158	0.025
Mazama nemorivaga	155,472	0.023
Alouatta	155,336	0.023
Tinamus	123,210	0.018
Hydrochoerus hydrochaeris	120,934	0.018
Tapirus terrestris	120,103	0.017
Ramphastos	115,082	0.017
Psophia	109,409	0.016
Cairina moschata	101,767	0.015
Iguana iguana	97,919	0.014
Sapajus	87,335	0.013
Dasypus sabanicola	82,128	0.012
Caiman crocodilus	81,649	0.012
Ateles	79,546	0.012
Nasua nasua	77,526	0.011
Mitu	76,095	0.011
Ardeidae (others)	69,908	0.010
Nannopterum brasilianus	59,158	0.009
Ardea alba	51,613	0.008
Crypturellus	49,365	0.007
Anura	48,338	0.007

North-western Amazonia (WAN)		
Taxon	Annual Individual Animals Offtake	Individual Animals Offtake proportion
Cuniculus paca	1,388,948	0.127
Tayassu pecari	1,208,027	0.110
Dicotyles tajacu	875,477	0.080
Dasyprocta	845,204	0.077
Dasypus novemcinctus	573,924	0.052
Penelope	462,329	0.042
Mazama americana	378,593	0.035
Tinamus	305,208	0.028
Chelonoidis	280,716	0.026
Mitu	266,804	0.024
Alouatta	254,500	0.023
Sapajus	226,213	0.021
Lagothrix	216,646	0.020
Mazama nemorivaga	213,890	0.020
Ramphastos	200,417	0.018
Ateles	163,532	0.015
Tapirus terrestris	161,981	0.015
Hydrochoerus hydrochaeris	134,350	0.012
Podocnemis unifilis	132,572	0.012
Myoprocta	127,201	0.012
Nasua nasua	121,446	0.011
Crypturellus	115,238	0.011
Sciurus (Hadrosciurus)	111,157	0.010
Ortalis	108,977	0.010
Anura	99,216	0.009
Psophia	98,173	0.009
Patagioenas	90,135	0.008
Ardeidae (others)	87,340	0.008
Amazona	86,546	0.008
Pipile	77,364	0.007

Central Amazonia (CA)		
Taxon	Annual Individual Animals Offtake	Individual Animals Offtake proportion
Cuniculus paca	1,009,460	0.160
Tayassu pecari	638,744	0.101
Dasyprocta	572,716	0.091
Dicotyles tajacu	387,073	0.061
Cairina moschata	258,833	0.041
Chelonoidis	244,807	0.039
Podocnemis sextuberculata	236,193	0.037
Podocnemis unifilis	226,787	0.036
Mitu	214,700	0.034
Dasypus novemcinctus	168,466	0.027
Mazama americana	163,460	0.026
Alouatta	141,829	0.022
Podocnemis erythrocephala	140,090	0.022
Nannopterum brasilianus	132,725	0.021
Mazama nemorivaga	132,507	0.021
Hydrochoerus hydrochaeris	111,934	0.018
Penelope	108,448	0.017
Podocnemis expansa	101,258	0.016
Peltocephalus dumerilianus	97,464	0.015
Caiman crocodilus	88,348	0.014
Tapirus terrestris	85,847	0.014
Sapajus	84,063	0.013
Lagothrix	76,994	0.012
Melanosuchus niger	68,654	0.011
Anura	64,092	0.010
Ateles	47,170	0.007
Tinamus	46,732	0.007
Nasua nasua	39,282	0.006
Psophia	38,470	0.006
Dendrocygna autumnalis	38,098	0.006

South-western Amazonia (WAS)		
Taxon	Annual Individual Animals Offtake	Individual Animals Offtake proportion
Cuniculus paca	1,090,272	0.121
Tayassu pecari	830,108	0.092
Dasyprocta	615,590	0.068
Dicotyles tajacu	550,100	0.061
Penelope	443,655	0.049
Dasypus novemcinctus	335,044	0.037
Mazama americana	315,315	0.035
Mitu	298,792	0.033
Chelonoidis	250,750	0.028
Alouatta	249,613	0.028
Ateles	241,238	0.027
Tinamus	237,331	0.026
Sapajus	174,921	0.019
Patagioenas	174,507	0.019
Hydrochoerus hydrochaeris	147,280	0.016
Ramphastos	141,568	0.016
Saimiri	137,535	0.015
Podocnemis unifilis	132,630	0.015
Crypturellus	128,460	0.014
Lagothrix	127,169	0.014
Nasua nasua	126,933	0.014
Sciurus (Hadrosciurus)	118,741	0.013
Ardeidae (others)	118,269	0.013
Mazama nemorivaga	111,005	0.012
Ortalis	106,663	0.012
Amazona	105,626	0.012
Tapirus terrestris	100,694	0.011
Odontophorus	93,543	0.010
Nannopterum brasilianus	87,318	0.010
Aotus	81,419	0.009

Southern Amazonia (SA)		
Taxon	Annual Individual Animals Offtake	Individual Animals Offtake proportion
Cuniculus paca	762,026	0.144
Tayassu pecari	684,568	0.130
Dicotyles tajacu	479,632	0.091
Dasyprocta	313,177	0.059
Penelope	284,375	0.054
Dasypus novemcinctus	272,162	0.052
Chelonoidis	257,820	0.049
Mazama americana	194,246	0.037
Sapajus	181,482	0.034
Mazama nemorivaga	180,823	0.034
Podocnemis unifilis	163,827	0.031
Mitu	144,047	0.027
Tinamus	139,936	0.026
Hydrochoerus hydrochaeris	123,640	0.023
Tapirus terrestris	88,814	0.017
Dasypus septemcinctus	79,538	0.015
Ateles	57,506	0.011
Alouatta	51,996	0.010
Crax	44,116	0.008
Nasua nasua	43,382	0.008
Crypturellus	35,669	0.007
Columbidae (others)	33,332	0.006
Pipile	32,741	0.006
Caiman crocodilus	32,194	0.006
Cairina moschata	29,163	0.006
Euphractus sexcinctus	28,959	0.005
Psophia	27,776	0.005
Amazona	27,152	0.005
Ramphastos	26,929	0.005
Ara	22,568	0.004

Eastern Amazonia (EA)		
Taxon	Annual Individual Animals Offtake	Individual Animals Offtake proportion
Cuniculus paca	693,384	0.131
Dasyprocta	546,179	0.103
Tayassu pecari	473,831	0.090
Chelonoidis	442,120	0.084
Dicotyles tajacu	339,936	0.064
Dasypus novemcinctus	216,696	0.041
Mazama americana	175,183	0.033
Hydrochoerus hydrochaeris	168,597	0.032
Podocnemis unifilis	159,266	0.030
Cairina moschata	133,074	0.025
Penelope	122,152	0.023
Iguana iguana	121,603	0.023
Alouatta	110,059	0.021
Podocnemis expansa	109,238	0.021
Nasua nasua	103,583	0.020
Euphractus sexcinctus	98,437	0.019
Mazama nemorivaga	95,304	0.018
Nannopterum brasilianus	92,536	0.018
Caiman crocodilus	92,202	0.017
Peltocephalus dumerilianus	90,898	0.017
Sapajus	77,316	0.015
Mitu	76,546	0.014
Tapirus terrestris	52,100	0.010
Melanosuchus niger	51,575	0.010
Tinamus	37,500	0.007
Crax	35,367	0.007
Dendrocygna autumnalis	34,173	0.006
Ramphastos	33,652	0.006
Dasypus septemcinctus	29,060	0.005
Priodontes maximus	28,058	0.005

Supplementary Table 5. Estimated proportion of the number of animals hunted (Individual Animals Offtake) per animal group in relation to the total offtake in each Amazonian region.

Guiana Shield (GS)		North-western Amazonia (WAN)		Central Amazonia (CA)		South-western Amazonia (WAS)		Southern Amazonia (SA)		Eastern Amazonia (EA)	
Animal group	Ind. Offtake proportion	Animal group	Ind. Offtake proportion	Animal group	Ind. Offtake proportion	Animal group	Ind. Offtake proportion	Animal group	Ind. Offtake proportion	Animal group	Ind. Offtake proportion
Ungulates	0.24680	Ungulates	0.25989	Large rodents	0.27163	Ungulates	0.21415	Ungulates	0.30924	Large rodents	0.27013
Large rodents	0.22612	Large rodents	0.23124	Ungulates	0.22263	Large rodents	0.21312	Large rodents	0.22815	Ungulates	0.21626
Cracids	0.08325	Primates	0.11167	River turtles	0.12915	Primates	0.12245	Cracids	0.09655	Tortoises	0.08366
Primates	0.06154	Cracids	0.08708	Aquatic birds	0.07892	Cracids	0.10485	Cingulates	0.07952	Cingulates	0.07625
Cingulates	0.05919	Cingulates	0.05563	Primates	0.06911	Terrestrial birds	0.05745	Primates	0.06899	River turtles	0.07152
Aquatic birds	0.05907	Terrestrial birds	0.05201	Cracids	0.05562	Aquatic birds	0.05578	Tortoises	0.04879	Aquatic birds	0.06356
Tortoises	0.04746	Aquatic birds	0.03481	Tortoises	0.03871	Cingulates	0.04408	Terrestrial birds	0.03919	Cracids	0.04593
Terrestrial birds	0.04323	Tortoises	0.02561	Cingulates	0.03528	Tortoises	0.02783	River turtles	0.03566	Primates	0.04407
River turtles	0.04144	Ramphastids	0.02074	Caimans	0.03044	Columbids	0.02425	Aquatic birds	0.01767	Caimans	0.02880
Caimans	0.02380	River turtles	0.01966	Terrestrial birds	0.01705	Large psittacids	0.02001	Caimans	0.01029	Lizards	0.02435
Lizards	0.01991	Procyonids	0.01469	Frogs	0.01013	Procyonids	0.01924	Procyonids	0.01020	Procyonids	0.02199
Ramphastids	0.01778	Small rodents	0.01383	Procyonids	0.00720	Small rodents	0.01845	Columbids	0.01000	Terrestrial birds	0.01469
Procyonids	0.01534	Large psittacids	0.01167	Large psittacids	0.00608	Ramphastids	0.01744	Large psittacids	0.00978	Large psittacids	0.00713
Large psittacids	0.01110	Columbids	0.01143	Ramphastids	0.00452	River turtles	0.01599	Felids	0.00928	Ramphastids	0.00679
Frogs	0.00703	Caimans	0.01102	Felids	0.00429	Small birds	0.01284	Ramphastids	0.00570	Felids	0.00479
Columbids	0.00643	Small birds	0.00919	Lizards	0.00300	Caimans	0.00684	Small birds	0.00365	Small birds	0.00470
Small rodents	0.00637	Frogs	0.00905	Myrmecophagids	0.00299	Lagomorphs	0.00633	Small rodents	0.00282	Columbids	0.00447
Small birds	0.00551	Felids	0.00503	Small birds	0.00286	Felids	0.00510	Lizards	0.00262	Myrmecophagids	0.00215
Felids	0.00542	Lagomorphs	0.00464	Small rodents	0.00235	Marsupials	0.00353	Myrmecophagids	0.00259	Folivores	0.00181
Myrmecophagids	0.00525	Myrmecophagids	0.00320	Mustelids	0.00188	Frogs	0.00307	Frogs	0.00219	Mustelids	0.00146
Folivores	0.00260	Marsupials	0.00265	Columbids	0.00179	Myrmecophagids	0.00195	Mustelids	0.00218	Frogs	0.00132
Mustelids	0.00187	Folivores	0.00175	Sirenians	0.00111	Raptor birds	0.00165	Lagomorphs	0.00173	Small rodents	0.00112
Marsupials	0.00116	Mustelids	0.00112	Folivores	0.00096	Mustelids	0.00101	Marsupials	0.00120	Marsupials	0.00104
Raptor birds	0.00071	Lizards	0.00077	Marsupials	0.00085	Lizards	0.00093	Raptor birds	0.00076	Raptor birds	0.00061
Snakes	0.00059	Raptor birds	0.00077	Snakes	0.00057	Folivores	0.00074	Canids	0.00047	Snakes	0.00061

Canids	0.00045	Snakes	0.00033	Raptor birds	0.00052	Canids	0.00044	Snakes	0.00045	Canids	0.00036
Lagomorphs	0.00042	Sirenians	0.00031	Cetaceans	0.00030	Snakes	0.00036	Folivores	0.00029	Sirenians	0.00017
Sirenians	0.00008	Bats	0.00010	Bats	0.00005	Bats	0.00004	Sirenians	0.00002	Lagomorphs	0.00014
Cetaceans	0.00005	Canids	0.00009	Canids	0.00002	Sirenians	0.00004	Cetaceans	0.00001	Cetaceans	0.00008
Bats	0.00002	Cetaceans	0.00003	Lagomorphs	0.00000	Cetaceans	0.00002	Bats	0.00000	Bats	0.00003
Ursid	0.00000	Ursid	0.00001	Ursid	0.00000	Ursid	0.00001	Ursid	0.00000	Ursid	0.00000

Supplementary Table 6. Estimated proportion of the number of animals hunted (Individual Animals Offtake) for the 30 most hunted taxa in relation to the total offtake in upland *terra firme* forests (regions > 50 % of upland *terra firme* forests) and flooded forests (regions > 50 % of flooded forests).

Upland terra firme forests (regions > 50 % of upland terra firme forests)			Flooded forests (regions > 50 % of flooded forests)	
Taxon	Individuals Offtake proportion		Taxon	Individuals Offtake proportion
Cuniculus paca	0.134		Cuniculus paca	0.133
Tayassu pecari	0.101		Tayassu pecari	0.109
Dicotyles tajacu	0.075		Dasyprocta	0.084
Dasyprocta	0.073		Dicotyles tajacu	0.065
Dasypus novemcinctus	0.047		Chelonoidis	0.051
Penelope	0.046		Podocnemis unifilis	0.041
Chelonoidis	0.036		Dasypus novemcinctus	0.031
Mazama americana	0.034		Mazama americana	0.030
Tinamus	0.027		Cairina moschata	0.029
Mitu	0.025		Alouatta	0.028
Mazama nemorivaga	0.022		Penelope	0.024
Sapajus	0.019		Mitu	0.024
Alouatta	0.019		Hydrochoerus hydrochaeris	0.022
Hydrochoerus hydrochaeris	0.016		Sapajus	0.019
Ateles	0.016		Mazama nemorivaga	0.018
Ramphastos	0.016		Podocnemis sextuberculata	0.016
Tapirus terrestris	0.014		Nasua nasua	0.015
Podocnemis unifilis	0.012		Nannopterum brasilianus	0.014
Patagioenas	0.011		Tapirus terrestris	0.014
Lagothrix	0.010		Caiman crocodilus	0.013
Crypturellus	0.010		Podocnemis expansa	0.012

Ardeidae (others)	0.010		Peltocephalus dumerilianus	0.011
Nasua nasua	0.010		Iguana iguana	0.010
Psophia	0.009		Ateles	0.009
Crax	0.008		Lagothrix	0.009
Ortalis	0.008		Tinamus	0.009
Amazona	0.008		Melanosuchus niger	0.009
Saimiri	0.007		Podocnemis erythrocephala	0.008
Sciurus (Hadrosciurus)	0.007		Ramphastos	0.007
Cairina moschata	0.007		Anura	0.006

Supplementary Table 7. Estimated proportion of the number of animals hunted (Individual Animals Offtake) per animal group in relation to the total offtake in upland *terra firme* forests (regions > 50 % of upland *terra firme* forests) and flooded forests (regions > 50 % of flooded forests).

Regions > 50 % of upland terra firme forests			Regions > 50 % of flooded forests	
Animal group	Individual Offtake proportion		Animal group	Individual Offtake proportion
Ungulates	0.24788		Large rodents	0.24751
Large rodents	0.23085		Ungulates	0.23634
Cracids	0.09403		River turtles	0.08909
Primates	0.08787		Primates	0.08417
Cingulates	0.06192		Cracids	0.06006
Terrestrial birds	0.05171		Aquatic birds	0.05916
Aquatic birds	0.04595		Tortoises	0.05130
Tortoises	0.03551		Cingulates	0.04621
River turtles	0.02220		Caimans	0.02556
Ramphastids	0.01730		Terrestrial birds	0.02105
Columbids	0.01544		Procyonids	0.01798
Procyonids	0.01330		Lizards	0.01179
Large psittacids	0.01298		Large psittacids	0.00947
Caimans	0.01222		Ramphastids	0.00745
Small rodents	0.01100		Frogs	0.00640
Small birds	0.00876		Small rodents	0.00569
Felids	0.00636		Small birds	0.00454
Frogs	0.00561		Felids	0.00392
Lizards	0.00461		Myrmecophagids	0.00323
Lagomorphs	0.00405		Columbids	0.00281
Myrmecophagids	0.00292		Mustelids	0.00143

Marsupials	0.00246		Folivores	0.00113
Folivores	0.00154		Marsupials	0.00110
Mustelids	0.00153		Sirenians	0.00067
Raptor birds	0.00104		Raptor birds	0.00061
Snakes	0.00043		Snakes	0.00050
Canids	0.00037		Lagomorphs	0.00048
Sirenians	0.00006		Cetaceans	0.00018
Bats	0.00005		Canids	0.00014
Cetaceans	0.00002		Bats	0.00005
Ursid	0.00001		Ursid	0.00000

Supplementary Data 4. Variable importance output of the 174 Taxon-Specific Offtake Proportion (TSOP) Random Forest models. For the purpose to show the variable importance for TSOP, we ran just one model with all the data.

Accipitridae

Urban-rural catchment areas (URCA)	0.00009
Annual Net Primary Productivity (NPP)	0.00008
Historical distribution of Indigenous family languages	0.00008
Elevation	0.00007
Enhanced Vegetation Index (EVI)	0.00007
Height above the nearest drainage (HAND)	0.00007
Annual Gross Primary Productivity (GPP)	0.00006
Soil fertility	0.00005
Proportion of habitat loss	0.00003
Current distribution of family languages	0.00003
Proportion of flooded areas	0.00002
Current distribution of Indigenous and non-indigenous peoples	0.00001
r2	-0.103

Alcedinidae

Historical distribution of Indigenous family languages	0.00002
Elevation	0.00002
Soil fertility	0.00002
Enhanced Vegetation Index (EVI)	0.00002
Annual Net Primary Productivity (NPP)	0.00002
Proportion of flooded areas	0.00001
Annual Gross Primary Productivity (GPP)	0.00001
Height above the nearest drainage (HAND)	0.00001
Current distribution of family languages	0.00000
Proportion of habitat loss	0.00000
Urban-rural catchment areas (URCA)	0.00000
Current distribution of Indigenous and non-indigenous peoples	0.00000
r2	-0.037

Alouatta

Soil fertility	0.22807
Enhanced Vegetation Index (EVI)	0.13177
Historical distribution of Indigenous family languages	0.12147
Annual Gross Primary Productivity (GPP)	0.12091
Proportion of flooded areas	0.10458
Current distribution of family languages	0.09533
Elevation	0.09370
Annual Net Primary Productivity (NPP)	0.08119
Height above the nearest drainage (HAND)	0.07919
Proportion of habitat loss	0.05450
Urban-rural catchment areas (URCA)	0.04958
Current distribution of Indigenous and non-indigenous peoples	0.00970
r2	0.274

Amazona

Soil fertility	0.01605
Enhanced Vegetation Index (EVI)	0.01357
Historical distribution of Indigenous family languages	0.00961
Annual Net Primary Productivity (NPP)	0.00926
Elevation	0.00919
Annual Gross Primary Productivity (GPP)	0.00865
Proportion of flooded areas	0.00842
Height above the nearest drainage (HAND)	0.00564
Current distribution of family languages	0.00531
Proportion of habitat loss	0.00476
Urban-rural catchment areas (URCA)	0.00436
Current distribution of Indigenous and non-indigenous peoples	0.00123
r2	-0.022

Ameiva ameiva

Current distribution of family languages	0.00293
Enhanced Vegetation Index (EVI)	0.00268
Annual Gross Primary Productivity (GPP)	0.00213
Annual Net Primary Productivity (NPP)	0.00094
Proportion of habitat loss	0.00062
Elevation	0.00051
Historical distribution of Indigenous family languages	0.00045
Soil fertility	0.00040
Height above the nearest drainage (HAND)	0.00034
Proportion of flooded areas	0.00016
Current distribution of Indigenous and non-indigenous peoples	0.00014
Urban-rural catchment areas (URCA)	0.00006
r2	-0.108

Anatidae (others)

Annual Net Primary Productivity (NPP)	0.00982
Annual Gross Primary Productivity (GPP)	0.00651
Historical distribution of Indigenous family languages	0.00604
Enhanced Vegetation Index (EVI)	0.00458
Elevation	0.00229
Height above the nearest drainage (HAND)	0.00227
Proportion of flooded areas	0.00078
Soil fertility	0.00077
Urban-rural catchment areas (URCA)	0.00076
Current distribution of family languages	0.00022
Proportion of habitat loss	0.00005
Current distribution of Indigenous and non-indigenous peoples	0.00000
r2	-0.378

Anhima cornuta

Current distribution of family languages	0
Annual Net Primary Productivity (NPP)	0
Height above the nearest drainage (HAND)	0
Proportion of flooded areas	0
Annual Gross Primary Productivity (GPP)	0
Elevation	0
Enhanced Vegetation Index (EVI)	0
Soil fertility	0
Proportion of habitat loss	0
Historical distribution of Indigenous family languages	0
Urban-rural catchment areas (URCA)	0
Current distribution of Indigenous and non-indigenous peoples	0
r2	-0.09

Anhinga anhinga

Annual Net Primary Productivity (NPP)	0.04608
Annual Gross Primary Productivity (GPP)	0.03680
Historical distribution of Indigenous family languages	0.01955
Soil fertility	0.01804
Height above the nearest drainage (HAND)	0.01508
Elevation	0.01441
Enhanced Vegetation Index (EVI)	0.01121
Proportion of flooded areas	0.00546
Current distribution of family languages	0.00285
Proportion of habitat loss	0.00195
Urban-rural catchment areas (URCA)	0.00154
Current distribution of Indigenous and non-indigenous peoples	0.00124
r2	-0.278

Anodorhynchus hyacinthinus

Urban-rural catchment areas (URCA)	0.00044
Enhanced Vegetation Index (EVI)	0.00018
Annual Net Primary Productivity (NPP)	0.00010
Proportion of habitat loss	0.00008
Proportion of flooded areas	0.00008
Annual Gross Primary Productivity (GPP)	0.00005
Height above the nearest drainage (HAND)	0.00003
Soil fertility	0.00003
Historical distribution of Indigenous family languages	0.00001
Current distribution of family languages	0.00001
Elevation	0.00001
Current distribution of Indigenous and non-indigenous peoples	0.00000
r2	-0.35

Anura

Annual Gross Primary Productivity (GPP)	0.26534
Annual Net Primary Productivity (NPP)	0.20595
Enhanced Vegetation Index (EVI)	0.17740
Soil fertility	0.17260
Height above the nearest drainage (HAND)	0.16449
Historical distribution of Indigenous family languages	0.14333
Current distribution of family languages	0.06288
Elevation	0.06158
Proportion of flooded areas	0.05055
Urban-rural catchment areas (URCA)	0.00304
Proportion of habitat loss	0.00142
Current distribution of Indigenous and non-indigenous peoples	0.00042
r2	0.207

Aotus

Annual Net Primary Productivity (NPP)	0.00721
Annual Gross Primary Productivity (GPP)	0.00582
Soil fertility	0.00503
Historical distribution of Indigenous family languages	0.00475
Elevation	0.00326
Current distribution of family languages	0.00258
Height above the nearest drainage (HAND)	0.00255
Enhanced Vegetation Index (EVI)	0.00237
Proportion of habitat loss	0.00193
Proportion of flooded areas	0.00182
Urban-rural catchment areas (URCA)	0.00103
Current distribution of Indigenous and non-indigenous peoples	0.00031
r2	0.03

Ara

Current distribution of family languages	0.01417
Historical distribution of Indigenous family languages	0.01267
Soil fertility	0.00691
Annual Gross Primary Productivity (GPP)	0.00605
Enhanced Vegetation Index (EVI)	0.00585
Elevation	0.00560
Annual Net Primary Productivity (NPP)	0.00559
Height above the nearest drainage (HAND)	0.00471
Proportion of habitat loss	0.00459
Proportion of flooded areas	0.00264
Current distribution of Indigenous and non-indigenous peoples	0.00113
Urban-rural catchment areas (URCA)	0.00099
r2	-0.182

Aramidae

Soil fertility	0.00075
Current distribution of family languages	0.00043
Historical distribution of Indigenous family languages	0.00025
Annual Gross Primary Productivity (GPP)	0.00025
Annual Net Primary Productivity (NPP)	0.00022
Height above the nearest drainage (HAND)	0.00012
Proportion of habitat loss	0.00010
Elevation	0.00009
Proportion of flooded areas	0.00005
Current distribution of Indigenous and non-indigenous peoples	0.00004
Enhanced Vegetation Index (EVI)	0.00004
Urban-rural catchment areas (URCA)	0.00000
r2	-0.097

Ardea alba

Annual Net Primary Productivity (NPP)	0.08079
Current distribution of family languages	0.05928
Annual Gross Primary Productivity (GPP)	0.04075
Proportion of flooded areas	0.03613
Historical distribution of Indigenous family languages	0.02153
Elevation	0.01875
Height above the nearest drainage (HAND)	0.01372
Soil fertility	0.01337
Enhanced Vegetation Index (EVI)	0.01013
Proportion of habitat loss	0.00201
Current distribution of Indigenous and non-indigenous peoples	0.00182
Urban-rural catchment areas (URCA)	0.00032
r2	-0.272

Ardea cocoi

Soil fertility	0.17827
Annual Gross Primary Productivity (GPP)	0.15280
Proportion of habitat loss	0.09301
Enhanced Vegetation Index (EVI)	0.06701
Annual Net Primary Productivity (NPP)	0.04337
Elevation	0.04114
Height above the nearest drainage (HAND)	0.03223
Current distribution of Indigenous and non-indigenous peoples	0.03094
Current distribution of family languages	0.02260
Proportion of flooded areas	0.01591
Urban-rural catchment areas (URCA)	0.00437
Historical distribution of Indigenous family languages	0.00211
r2	-0.316

Ardeidae (others)

Current distribution of family languages	0.38747
Annual Net Primary Productivity (NPP)	0.11748
Annual Gross Primary Productivity (GPP)	0.05731
Proportion of flooded areas	0.04797
Height above the nearest drainage (HAND)	0.03819
Enhanced Vegetation Index (EVI)	0.02301
Soil fertility	0.01940
Elevation	0.01861
Historical distribution of Indigenous family languages	0.01469
Current distribution of Indigenous and non-indigenous peoples	0.00544
Urban-rural catchment areas (URCA)	0.00079
Proportion of habitat loss	0.00039
r2	-0.366

Ateles

Soil fertility	0.37514
Elevation	0.28808
Height above the nearest drainage (HAND)	0.19986
Historical distribution of Indigenous family languages	0.18352
Annual Gross Primary Productivity (GPP)	0.18028
Annual Net Primary Productivity (NPP)	0.17600
Current distribution of family languages	0.16530
Proportion of flooded areas	0.15145
Enhanced Vegetation Index (EVI)	0.12260
Proportion of habitat loss	0.03950
Current distribution of Indigenous and non-indigenous peoples	0.02665
Urban-rural catchment areas (URCA)	0.00963
r2	0.266

Atelocynus microtis

Annual Net Primary Productivity (NPP)	0
Annual Gross Primary Productivity (GPP)	0
Elevation	0
Current distribution of family languages	0
Enhanced Vegetation Index (EVI)	0
Soil fertility	0
Height above the nearest drainage (HAND)	0
Proportion of flooded areas	0
Historical distribution of Indigenous family languages	0
Urban-rural catchment areas (URCA)	0
Proportion of habitat loss	0
Current distribution of Indigenous and non-indigenous peoples	0
r2	-0.089

Bassaricyon

Historical distribution of Indigenous family languages	0.00001
Elevation	0.00000
Annual Net Primary Productivity (NPP)	0.00000
Soil fertility	0.00000
Annual Gross Primary Productivity (GPP)	0.00000
Urban-rural catchment areas (URCA)	0.00000
Proportion of habitat loss	0.00000
Height above the nearest drainage (HAND)	0.00000
Proportion of flooded areas	0.00000
Enhanced Vegetation Index (EVI)	0.00000
Current distribution of family languages	0.00000
Current distribution of Indigenous and non-indigenous peoples	0.00000
r2	-0.127

Blastocerus dichotomus

Elevation	0.00109
Annual Gross Primary Productivity (GPP)	0.00060
Annual Net Primary Productivity (NPP)	0.00045
Soil fertility	0.00021
Historical distribution of Indigenous family languages	0.00017
Enhanced Vegetation Index (EVI)	0.00012
Proportion of flooded areas	0.00010
Height above the nearest drainage (HAND)	0.00008
Proportion of habitat loss	0.00008
Current distribution of Indigenous and non-indigenous peoples	0.00002
Urban-rural catchment areas (URCA)	0.00001
Current distribution of family languages	0.00000
r2	0.083

Boa constrictor

Annual Net Primary Productivity (NPP)	0.00010
Annual Gross Primary Productivity (GPP)	0.00009
Enhanced Vegetation Index (EVI)	0.00009
Soil fertility	0.00009
Current distribution of family languages	0.00008
Height above the nearest drainage (HAND)	0.00008
Historical distribution of Indigenous family languages	0.00008
Elevation	0.00007
Proportion of flooded areas	0.00004
Urban-rural catchment areas (URCA)	0.00002
Proportion of habitat loss	0.00001
Current distribution of Indigenous and non-indigenous peoples	0.00000
r2	-0.196

Bradypus

Annual Net Primary Productivity (NPP)	0.00108
Annual Gross Primary Productivity (GPP)	0.00075
Soil fertility	0.00034
Elevation	0.00032
Height above the nearest drainage (HAND)	0.00028
Historical distribution of Indigenous family languages	0.00025
Proportion of flooded areas	0.00024
Enhanced Vegetation Index (EVI)	0.00017
Proportion of habitat loss	0.00010
Urban-rural catchment areas (URCA)	0.00010
Current distribution of family languages	0.00008
Current distribution of Indigenous and non-indigenous peoples	0.00005
r2	-0.14

Bucconidae

Historical distribution of Indigenous family languages	0.00003
Current distribution of family languages	0.00001
Soil fertility	0.00000
Annual Gross Primary Productivity (GPP)	0.00000
Annual Net Primary Productivity (NPP)	0.00000
Enhanced Vegetation Index (EVI)	0.00000
Height above the nearest drainage (HAND)	0.00000
Elevation	0.00000
Urban-rural catchment areas (URCA)	0.00000
Proportion of habitat loss	0.00000
Proportion of flooded areas	0.00000
Current distribution of Indigenous and non-indigenous peoples	0.00000
r2	-0.057

Burhinus bistriatus

Historical distribution of Indigenous family languages	0.00001
Enhanced Vegetation Index (EVI)	0.00001
Current distribution of family languages	0.00001
Proportion of flooded areas	0.00001
Height above the nearest drainage (HAND)	0.00001
Annual Gross Primary Productivity (GPP)	0.00001
Annual Net Primary Productivity (NPP)	0.00001
Current distribution of Indigenous and non-indigenous peoples	0.00000
Urban-rural catchment areas (URCA)	0.00000
Soil fertility	0.00000
Elevation	0.00000
Proportion of habitat loss	0.00000
r2	-0.33

Cabassous unicinctus

Elevation	0.00294
Annual Gross Primary Productivity (GPP)	0.00179
Enhanced Vegetation Index (EVI)	0.00172
Soil fertility	0.00134
Annual Net Primary Productivity (NPP)	0.00126
Proportion of flooded areas	0.00123
Height above the nearest drainage (HAND)	0.00121
Urban-rural catchment areas (URCA)	0.00116
Historical distribution of Indigenous family languages	0.00098
Proportion of habitat loss	0.00090
Current distribution of family languages	0.00015
Current distribution of Indigenous and non-indigenous peoples	0.00003
r2	-0.066

Cacajao

Enhanced Vegetation Index (EVI)	0.00637
Annual Net Primary Productivity (NPP)	0.00521
Historical distribution of Indigenous family languages	0.00433
Elevation	0.00421
Annual Gross Primary Productivity (GPP)	0.00400
Soil fertility	0.00336
Height above the nearest drainage (HAND)	0.00332
Current distribution of family languages	0.00247
Proportion of flooded areas	0.00243
Current distribution of Indigenous and non-indigenous peoples	0.00032
Proportion of habitat loss	0.00032
Urban-rural catchment areas (URCA)	0.00003
r2	0.082

Caiman crocodilus

Elevation	0.04993
Height above the nearest drainage (HAND)	0.04467
Enhanced Vegetation Index (EVI)	0.03786
Annual Gross Primary Productivity (GPP)	0.02784
Soil fertility	0.02753
Current distribution of family languages	0.02353
Historical distribution of Indigenous family languages	0.01986
Annual Net Primary Productivity (NPP)	0.01946
Proportion of flooded areas	0.01735
Proportion of habitat loss	0.00543
Urban-rural catchment areas (URCA)	0.00360
Current distribution of Indigenous and non-indigenous peoples	0.00143
r2	-0.224

Caiman yacare

Annual Net Primary Productivity (NPP)	0
Proportion of flooded areas	0
Annual Gross Primary Productivity (GPP)	0
Elevation	0
Enhanced Vegetation Index (EVI)	0
Historical distribution of Indigenous family languages	0
Height above the nearest drainage (HAND)	0
Soil fertility	0
Current distribution of family languages	0
Proportion of habitat loss	0
Urban-rural catchment areas (URCA)	0
Current distribution of Indigenous and non-indigenous peoples	0
r2	-0.219

Cairina moschata

Proportion of habitat loss	0.20916
Annual Net Primary Productivity (NPP)	0.19575
Historical distribution of Indigenous family languages	0.19344
Elevation	0.15076
Annual Gross Primary Productivity (GPP)	0.14199
Proportion of flooded areas	0.13227
Soil fertility	0.12221
Enhanced Vegetation Index (EVI)	0.10262
Height above the nearest drainage (HAND)	0.06762
Current distribution of Indigenous and non-indigenous peoples	0.01223
Urban-rural catchment areas (URCA)	0.00909
Current distribution of family languages	0.00812
r2	0.031

Callimico goeldii

Height above the nearest drainage (HAND)	0.00003
Elevation	0.00001
Proportion of flooded areas	0.00001
Soil fertility	0.00001
Annual Net Primary Productivity (NPP)	0.00001
Current distribution of family languages	0.00001
Enhanced Vegetation Index (EVI)	0.00000
Proportion of habitat loss	0.00000
Annual Gross Primary Productivity (GPP)	0.00000
Current distribution of Indigenous and non-indigenous peoples	0.00000
Historical distribution of Indigenous family languages	0.00000
Urban-rural catchment areas (URCA)	0.00000
r2	-0.145

Cariama cristata

Proportion of habitat loss	0.00001
Height above the nearest drainage (HAND)	0.00001
Urban-rural catchment areas (URCA)	0.00001
Soil fertility	0.00001
Annual Net Primary Productivity (NPP)	0.00001
Enhanced Vegetation Index (EVI)	0.00000
Historical distribution of Indigenous family languages	0.00000
Elevation	0.00000
Annual Gross Primary Productivity (GPP)	0.00000
Proportion of flooded areas	0.00000
Current distribution of family languages	0.00000
Current distribution of Indigenous and non-indigenous peoples	0.00000
r2	-0.436

Cathartidae (others)

Annual Gross Primary Productivity (GPP)	0.00004
Annual Net Primary Productivity (NPP)	0.00003
Enhanced Vegetation Index (EVI)	0.00002
Elevation	0.00001
Urban-rural catchment areas (URCA)	0.00001
Height above the nearest drainage (HAND)	0.00001
Proportion of flooded areas	0.00001
Soil fertility	0.00001
Proportion of habitat loss	0.00001
Historical distribution of Indigenous family languages	0.00000
Current distribution of Indigenous and non-indigenous peoples	0.00000
Current distribution of family languages	0.00000
r2	-0.066

Cavia

Proportion of habitat loss	0.00137
Urban-rural catchment areas (URCA)	0.00105
Elevation	0.00101
Enhanced Vegetation Index (EVI)	0.00020
Height above the nearest drainage (HAND)	0.00017
Historical distribution of Indigenous family languages	0.00010
Proportion of flooded areas	0.00005
Annual Net Primary Productivity (NPP)	0.00004
Soil fertility	0.00003
Annual Gross Primary Productivity (GPP)	0.00001
Current distribution of family languages	0.00000
Current distribution of Indigenous and non-indigenous peoples	0.00000
r2	-0.15

Cebuella

Urban-rural catchment areas (URCA)	0.00001
Proportion of flooded areas	0.00000
Historical distribution of Indigenous family languages	0.00000
Annual Net Primary Productivity (NPP)	0.00000
Current distribution of family languages	0.00000
Annual Gross Primary Productivity (GPP)	0.00000
Proportion of habitat loss	0.00000
Height above the nearest drainage (HAND)	0.00000
Elevation	0.00000
Soil fertility	0.00000
Enhanced Vegetation Index (EVI)	0.00000
Current distribution of Indigenous and non-indigenous peoples	0.00000
r2	-0.075

Cebus

Annual Gross Primary Productivity (GPP)	0.01575
Current distribution of family languages	0.01096
Proportion of flooded areas	0.00897
Historical distribution of Indigenous family languages	0.00721
Enhanced Vegetation Index (EVI)	0.00714
Soil fertility	0.00685
Annual Net Primary Productivity (NPP)	0.00680
Elevation	0.00629
Height above the nearest drainage (HAND)	0.00622
Proportion of habitat loss	0.00475
Current distribution of Indigenous and non-indigenous peoples	0.00077
Urban-rural catchment areas (URCA)	0.00048
r2	0.369

Cerdocyon thous

Proportion of habitat loss	0.00010
Urban-rural catchment areas (URCA)	0.00004
Height above the nearest drainage (HAND)	0.00004
Historical distribution of Indigenous family languages	0.00001
Soil fertility	0.00001
Enhanced Vegetation Index (EVI)	0.00000
Proportion of flooded areas	0.00000
Annual Net Primary Productivity (NPP)	0.00000
Current distribution of Indigenous and non-indigenous peoples	0.00000
Annual Gross Primary Productivity (GPP)	0.00000
Current distribution of family languages	0.00000
Elevation	0.00000
r2	-0.126

Chamaepetes goudotii

Annual Net Primary Productivity (NPP)	0
Current distribution of family languages	0
Height above the nearest drainage (HAND)	0
Annual Gross Primary Productivity (GPP)	0
Proportion of flooded areas	0
Proportion of habitat loss	0
Historical distribution of Indigenous family languages	0
Elevation	0
Soil fertility	0
Enhanced Vegetation Index (EVI)	0
Urban-rural catchment areas (URCA)	0
Current distribution of Indigenous and non-indigenous peoples	0
r2	-0.939

Chauna torquata

Proportion of flooded areas	0
Annual Net Primary Productivity (NPP)	0
Elevation	0
Current distribution of family languages	0
Annual Gross Primary Productivity (GPP)	0
Historical distribution of Indigenous family languages	0
Enhanced Vegetation Index (EVI)	0
Soil fertility	0
Current distribution of Indigenous and non-indigenous peoples	0
Height above the nearest drainage (HAND)	0
Urban-rural catchment areas (URCA)	0
Proportion of habitat loss	0
r2	-0.251

Chelonoidis

Annual Gross Primary Productivity (GPP)	0.49911
Enhanced Vegetation Index (EVI)	0.46583
Elevation	0.42863
Soil fertility	0.31632
Current distribution of family languages	0.30406
Annual Net Primary Productivity (NPP)	0.28240
Historical distribution of Indigenous family languages	0.24337
Height above the nearest drainage (HAND)	0.20538
Proportion of flooded areas	0.19142
Proportion of habitat loss	0.12671
Urban-rural catchment areas (URCA)	0.06383
Current distribution of Indigenous and non-indigenous peoples	0.02601
r2	0.068

Chelus fimbriata

Annual Net Primary Productivity (NPP)	0.00031
Annual Gross Primary Productivity (GPP)	0.00018
Soil fertility	0.00005
Elevation	0.00005
Historical distribution of Indigenous family languages	0.00005
Urban-rural catchment areas (URCA)	0.00004
Proportion of flooded areas	0.00003
Enhanced Vegetation Index (EVI)	0.00003
Height above the nearest drainage (HAND)	0.00003
Current distribution of family languages	0.00002
Proportion of habitat loss	0.00001
Current distribution of Indigenous and non-indigenous peoples	0.00000
r2	-0.111

Cheracebus

Height above the nearest drainage (HAND)	0.00412
Annual Net Primary Productivity (NPP)	0.00228
Enhanced Vegetation Index (EVI)	0.00213
Annual Gross Primary Productivity (GPP)	0.00191
Proportion of flooded areas	0.00178
Elevation	0.00160
Historical distribution of Indigenous family languages	0.00153
Current distribution of family languages	0.00112
Soil fertility	0.00105
Current distribution of Indigenous and non-indigenous peoples	0.00011
Proportion of habitat loss	0.00006
Urban-rural catchment areas (URCA)	0.00001
r2	0.052

Chiropotes

Current distribution of family languages	0.00365
Enhanced Vegetation Index (EVI)	0.00303
Annual Net Primary Productivity (NPP)	0.00236
Historical distribution of Indigenous family languages	0.00104
Height above the nearest drainage (HAND)	0.00102
Elevation	0.00088
Soil fertility	0.00077
Proportion of flooded areas	0.00062
Annual Gross Primary Productivity (GPP)	0.00053
Proportion of habitat loss	0.00048
Current distribution of Indigenous and non-indigenous peoples	0.00028
Urban-rural catchment areas (URCA)	0.00020
r2	-0.052

Chiroptera

Historical distribution of Indigenous family languages	0.00026
Annual Net Primary Productivity (NPP)	0.00003
Annual Gross Primary Productivity (GPP)	0.00003
Soil fertility	0.00002
Elevation	0.00002
Enhanced Vegetation Index (EVI)	0.00001
Proportion of flooded areas	0.00001
Height above the nearest drainage (HAND)	0.00001
Urban-rural catchment areas (URCA)	0.00000
Current distribution of Indigenous and non-indigenous peoples	0.00000
Proportion of habitat loss	0.00000
Current distribution of family languages	0.00000
r2	-0.01

Choloepus

Annual Net Primary Productivity (NPP)	0.00717
Annual Gross Primary Productivity (GPP)	0.00470
Historical distribution of Indigenous family languages	0.00453
Proportion of flooded areas	0.00391
Soil fertility	0.00362
Elevation	0.00347
Height above the nearest drainage (HAND)	0.00200
Enhanced Vegetation Index (EVI)	0.00161
Current distribution of family languages	0.00138
Proportion of habitat loss	0.00034
Urban-rural catchment areas (URCA)	0.00014
Current distribution of Indigenous and non-indigenous peoples	0.00003
r2	-0.047

Ciconia maguari

Soil fertility	0.00009
Current distribution of family languages	0.00008
Annual Net Primary Productivity (NPP)	0.00002
Enhanced Vegetation Index (EVI)	0.00002
Proportion of flooded areas	0.00002
Historical distribution of Indigenous family languages	0.00001
Annual Gross Primary Productivity (GPP)	0.00001
Elevation	0.00001
Proportion of habitat loss	0.00000
Height above the nearest drainage (HAND)	0.00000
Urban-rural catchment areas (URCA)	0.00000
Current distribution of Indigenous and non-indigenous peoples	0.00000
r2	-0.172

Coendou

Soil fertility	0.00069
Current distribution of family languages	0.00057
Annual Net Primary Productivity (NPP)	0.00053
Height above the nearest drainage (HAND)	0.00052
Historical distribution of Indigenous family languages	0.00051
Elevation	0.00051
Annual Gross Primary Productivity (GPP)	0.00045
Enhanced Vegetation Index (EVI)	0.00038
Proportion of flooded areas	0.00038
Urban-rural catchment areas (URCA)	0.00033
Proportion of habitat loss	0.00015
Current distribution of Indigenous and non-indigenous peoples	0.00005
r2	-0.165

Colinus cristatus

Current distribution of family languages	0.00017
Enhanced Vegetation Index (EVI)	0.00010
Soil fertility	0.00009
Historical distribution of Indigenous family languages	0.00003
Annual Net Primary Productivity (NPP)	0.00002
Annual Gross Primary Productivity (GPP)	0.00002
Elevation	0.00001
Proportion of flooded areas	0.00001
Height above the nearest drainage (HAND)	0.00000
Proportion of habitat loss	0.00000
Urban-rural catchment areas (URCA)	0.00000
Current distribution of Indigenous and non-indigenous peoples	0.00000
r2	-0.077

Columbidae (others)

Annual Gross Primary Productivity (GPP)	0.00881
Urban-rural catchment areas (URCA)	0.00829
Proportion of habitat loss	0.00672
Proportion of flooded areas	0.00541
Enhanced Vegetation Index (EVI)	0.00474
Height above the nearest drainage (HAND)	0.00451
Elevation	0.00447
Annual Net Primary Productivity (NPP)	0.00415
Historical distribution of Indigenous family languages	0.00403
Soil fertility	0.00224
Current distribution of family languages	0.00005
Current distribution of Indigenous and non-indigenous peoples	0.00001
r2	-0.102

Cotingidae

Soil fertility	0.00264
Annual Gross Primary Productivity (GPP)	0.00217
Enhanced Vegetation Index (EVI)	0.00099
Height above the nearest drainage (HAND)	0.00097
Annual Net Primary Productivity (NPP)	0.00095
Elevation	0.00085
Proportion of flooded areas	0.00044
Current distribution of family languages	0.00029
Historical distribution of Indigenous family languages	0.00024
Urban-rural catchment areas (URCA)	0.00009
Proportion of habitat loss	0.00006
Current distribution of Indigenous and non-indigenous peoples	0.00001
r2	-0.08

Crax

Annual Net Primary Productivity (NPP)	0.02570
Historical distribution of Indigenous family languages	0.02405
Current distribution of family languages	0.02386
Elevation	0.01940
Annual Gross Primary Productivity (GPP)	0.01722
Height above the nearest drainage (HAND)	0.01574
Proportion of flooded areas	0.01153
Soil fertility	0.01141
Enhanced Vegetation Index (EVI)	0.01030
Urban-rural catchment areas (URCA)	0.00809
Proportion of habitat loss	0.00550
Current distribution of Indigenous and non-indigenous peoples	0.00127
r2	0.173

Cricetidae

Historical distribution of Indigenous family languages	0.00043
Proportion of flooded areas	0.00020
Annual Net Primary Productivity (NPP)	0.00018
Elevation	0.00016
Height above the nearest drainage (HAND)	0.00015
Annual Gross Primary Productivity (GPP)	0.00010
Urban-rural catchment areas (URCA)	0.00007
Enhanced Vegetation Index (EVI)	0.00005
Proportion of habitat loss	0.00005
Soil fertility	0.00003
Current distribution of Indigenous and non-indigenous peoples	0.00001
Current distribution of family languages	0.00000
r2	-0.322

Crypturellus

Historical distribution of Indigenous family languages	0.03115
Proportion of flooded areas	0.02517
Soil fertility	0.01897
Annual Gross Primary Productivity (GPP)	0.01852
Enhanced Vegetation Index (EVI)	0.01826
Annual Net Primary Productivity (NPP)	0.01695
Height above the nearest drainage (HAND)	0.01573
Elevation	0.01283
Proportion of habitat loss	0.00976
Current distribution of family languages	0.00727
Urban-rural catchment areas (URCA)	0.00613
Current distribution of Indigenous and non-indigenous peoples	0.00127
r2	0.054

Cuculidae

Historical distribution of Indigenous family languages	0.00008
Current distribution of family languages	0.00004
Elevation	0.00004
Annual Gross Primary Productivity (GPP)	0.00004
Soil fertility	0.00003
Annual Net Primary Productivity (NPP)	0.00003
Urban-rural catchment areas (URCA)	0.00002
Height above the nearest drainage (HAND)	0.00002
Proportion of habitat loss	0.00001
Proportion of flooded areas	0.00001
Enhanced Vegetation Index (EVI)	0.00001
Current distribution of Indigenous and non-indigenous peoples	0.00000
r2	-0.212

Cuniculus paca

Historical distribution of Indigenous family languages	1.37794
Soil fertility	1.35758
Elevation	1.22205
Current distribution of family languages	1.21857
Proportion of habitat loss	0.96445
Annual Gross Primary Productivity (GPP)	0.84584
Annual Net Primary Productivity (NPP)	0.81585
Height above the nearest drainage (HAND)	0.76454
Enhanced Vegetation Index (EVI)	0.72494
Proportion of flooded areas	0.70241
Urban-rural catchment areas (URCA)	0.44614
Current distribution of Indigenous and non-indigenous peoples	0.14763
r2	0.276

Cyclopes didactylus

Soil fertility	0.00001
Enhanced Vegetation Index (EVI)	0.00001
Historical distribution of Indigenous family languages	0.00001
Height above the nearest drainage (HAND)	0.00001
Elevation	0.00001
Annual Net Primary Productivity (NPP)	0.00001
Proportion of flooded areas	0.00001
Current distribution of family languages	0.00001
Annual Gross Primary Productivity (GPP)	0.00000
Urban-rural catchment areas (URCA)	0.00000
Proportion of habitat loss	0.00000
Current distribution of Indigenous and non-indigenous peoples	0.00000
r2	-0.014

Dasyprocta

Historical distribution of Indigenous family languages	0.58463
Elevation	0.52181
Soil fertility	0.46953
Height above the nearest drainage (HAND)	0.39623
Annual Gross Primary Productivity (GPP)	0.34158
Annual Net Primary Productivity (NPP)	0.33674
Enhanced Vegetation Index (EVI)	0.32127
Proportion of flooded areas	0.31547
Urban-rural catchment areas (URCA)	0.29262
Proportion of habitat loss	0.28112
Current distribution of family languages	0.25963
Current distribution of Indigenous and non-indigenous peoples	0.04032
r2	0.188

Dasypus kappleri

Urban-rural catchment areas (URCA)	0.00586
Enhanced Vegetation Index (EVI)	0.00441
Current distribution of family languages	0.00387
Annual Gross Primary Productivity (GPP)	0.00311
Historical distribution of Indigenous family languages	0.00291
Height above the nearest drainage (HAND)	0.00243
Soil fertility	0.00232
Proportion of habitat loss	0.00230
Proportion of flooded areas	0.00173
Elevation	0.00172
Annual Net Primary Productivity (NPP)	0.00170
Current distribution of Indigenous and non-indigenous peoples	0.00007
r2	0.092

Dasypus novemcinctus

Urban-rural catchment areas (URCA)	0.29000
Annual Gross Primary Productivity (GPP)	0.23813
Proportion of habitat loss	0.23720
Height above the nearest drainage (HAND)	0.18937
Annual Net Primary Productivity (NPP)	0.17083
Historical distribution of Indigenous family languages	0.14496
Soil fertility	0.13610
Elevation	0.11614
Enhanced Vegetation Index (EVI)	0.11507
Proportion of flooded areas	0.08805
Current distribution of family languages	0.04513
Current distribution of Indigenous and non-indigenous peoples	0.00633
r2	0.181

Dasypus sabanicola

Proportion of habitat loss	0.01366
Historical distribution of Indigenous family languages	0.01164
Soil fertility	0.01015
Annual Gross Primary Productivity (GPP)	0.00896
Annual Net Primary Productivity (NPP)	0.00888
Proportion of flooded areas	0.00806
Elevation	0.00642
Enhanced Vegetation Index (EVI)	0.00627
Urban-rural catchment areas (URCA)	0.00440
Height above the nearest drainage (HAND)	0.00246
Current distribution of Indigenous and non-indigenous peoples	0.00000
Current distribution of family languages	0.00000
r2	-0.376

Dasypus septemcinctus

Elevation	0.05058
Soil fertility	0.04317
Height above the nearest drainage (HAND)	0.04135
Enhanced Vegetation Index (EVI)	0.01745
Proportion of habitat loss	0.01383
Proportion of flooded areas	0.01083
Annual Net Primary Productivity (NPP)	0.00945
Current distribution of family languages	0.00874
Annual Gross Primary Productivity (GPP)	0.00844
Urban-rural catchment areas (URCA)	0.00384
Historical distribution of Indigenous family languages	0.00284
Current distribution of Indigenous and non-indigenous peoples	0.00111
r2	0.025

Dendrocygna autumnalis

Enhanced Vegetation Index (EVI)	0.01932
Soil fertility	0.01824
Annual Gross Primary Productivity (GPP)	0.01576
Elevation	0.01461
Proportion of flooded areas	0.01244
Historical distribution of Indigenous family languages	0.01162
Annual Net Primary Productivity (NPP)	0.01088
Height above the nearest drainage (HAND)	0.00793
Proportion of habitat loss	0.00227
Urban-rural catchment areas (URCA)	0.00158
Current distribution of family languages	0.00056
Current distribution of Indigenous and non-indigenous peoples	0.00015
r2	-0.063

Dicotyles tajacu

Historical distribution of Indigenous family languages	0.65964
Current distribution of family languages	0.46179
Height above the nearest drainage (HAND)	0.45765
Enhanced Vegetation Index (EVI)	0.43086
Proportion of flooded areas	0.39912
Elevation	0.38840
Soil fertility	0.38719
Annual Net Primary Productivity (NPP)	0.37933
Annual Gross Primary Productivity (GPP)	0.32508
Proportion of habitat loss	0.22661
Urban-rural catchment areas (URCA)	0.16588
Current distribution of Indigenous and non-indigenous peoples	0.04504
r2	0.13

Didelphidae (others)

Historical distribution of Indigenous family languages	0
Elevation	0
Annual Net Primary Productivity (NPP)	0
Urban-rural catchment areas (URCA)	0
Proportion of flooded areas	0
Soil fertility	0
Annual Gross Primary Productivity (GPP)	0
Height above the nearest drainage (HAND)	0
Enhanced Vegetation Index (EVI)	0
Proportion of habitat loss	0
Current distribution of family languages	0
Current distribution of Indigenous and non-indigenous peoples	0
r2	-0.079

Didelphis

Enhanced Vegetation Index (EVI)	0.00129
Annual Gross Primary Productivity (GPP)	0.00115
Historical distribution of Indigenous family languages	0.00104
Soil fertility	0.00093
Annual Net Primary Productivity (NPP)	0.00076
Proportion of habitat loss	0.00061
Elevation	0.00053
Proportion of flooded areas	0.00052
Height above the nearest drainage (HAND)	0.00048
Urban-rural catchment areas (URCA)	0.00032
Current distribution of family languages	0.00031
Current distribution of Indigenous and non-indigenous peoples	0.00014
r2	-0.059

Dinomys branickii

Height above the nearest drainage (HAND)	0.00041
Elevation	0.00030
Soil fertility	0.00024
Annual Gross Primary Productivity (GPP)	0.00020
Annual Net Primary Productivity (NPP)	0.00015
Proportion of flooded areas	0.00011
Enhanced Vegetation Index (EVI)	0.00011
Current distribution of family languages	0.00011
Historical distribution of Indigenous family languages	0.00007
Proportion of habitat loss	0.00007
Urban-rural catchment areas (URCA)	0.00003
Current distribution of Indigenous and non-indigenous peoples	0.00001
r2	-0.147

Dracaena guianensis

Current distribution of family languages	0.00004
Height above the nearest drainage (HAND)	0.00001
Proportion of habitat loss	0.00001
Annual Net Primary Productivity (NPP)	0.00001
Elevation	0.00001
Enhanced Vegetation Index (EVI)	0.00000
Proportion of flooded areas	0.00000
Urban-rural catchment areas (URCA)	0.00000
Historical distribution of Indigenous family languages	0.00000
Annual Gross Primary Productivity (GPP)	0.00000
Soil fertility	0.00000
Current distribution of Indigenous and non-indigenous peoples	0.00000
r2	-0.08

Echimyidae

Historical distribution of Indigenous family languages	0.08212
Proportion of flooded areas	0.06086
Annual Net Primary Productivity (NPP)	0.04838
Annual Gross Primary Productivity (GPP)	0.04212
Elevation	0.02993
Height above the nearest drainage (HAND)	0.02770
Proportion of habitat loss	0.02398
Soil fertility	0.01311
Urban-rural catchment areas (URCA)	0.01015
Enhanced Vegetation Index (EVI)	0.00897
Current distribution of Indigenous and non-indigenous peoples	0.00382
Current distribution of family languages	0.00041
r2	-0.312

Eira barbara

Soil fertility	0.00134
Height above the nearest drainage (HAND)	0.00119
Proportion of flooded areas	0.00089
Historical distribution of Indigenous family languages	0.00083
Elevation	0.00079
Annual Net Primary Productivity (NPP)	0.00077
Enhanced Vegetation Index (EVI)	0.00072
Annual Gross Primary Productivity (GPP)	0.00069
Proportion of habitat loss	0.00069
Urban-rural catchment areas (URCA)	0.00037
Current distribution of family languages	0.00037
Current distribution of Indigenous and non-indigenous peoples	0.00003
r2	-0.06

Eudocimus ruber

Historical distribution of Indigenous family languages	0.00029
Proportion of flooded areas	0.00024
Elevation	0.00021
Current distribution of family languages	0.00012
Annual Net Primary Productivity (NPP)	0.00006
Current distribution of Indigenous and non-indigenous peoples	0.00004
Annual Gross Primary Productivity (GPP)	0.00002
Soil fertility	0.00001
Enhanced Vegetation Index (EVI)	0.00001
Proportion of habitat loss	0.00000
Urban-rural catchment areas (URCA)	0.00000
Height above the nearest drainage (HAND)	0.00000
r2	-0.108

Eunectes murinus

Historical distribution of Indigenous family languages	0.00008
Current distribution of family languages	0.00007
Annual Gross Primary Productivity (GPP)	0.00007
Annual Net Primary Productivity (NPP)	0.00006
Proportion of flooded areas	0.00005
Height above the nearest drainage (HAND)	0.00005
Elevation	0.00005
Soil fertility	0.00004
Proportion of habitat loss	0.00003
Enhanced Vegetation Index (EVI)	0.00003
Urban-rural catchment areas (URCA)	0.00001
Current distribution of Indigenous and non-indigenous peoples	0.00000
r2	-0.024

Euphractus sexcinctus

Annual Gross Primary Productivity (GPP)	0.17929
Elevation	0.14168
Proportion of flooded areas	0.07315
Annual Net Primary Productivity (NPP)	0.06640
Height above the nearest drainage (HAND)	0.05677
Urban-rural catchment areas (URCA)	0.04770
Enhanced Vegetation Index (EVI)	0.03798
Soil fertility	0.03408
Proportion of habitat loss	0.01513
Current distribution of Indigenous and non-indigenous peoples	0.00260
Historical distribution of Indigenous family languages	0.00179
Current distribution of family languages	0.00009
r2	-0.267

Eurypyga helias

Elevation	0
Annual Net Primary Productivity (NPP)	0
Proportion of flooded areas	0
Historical distribution of Indigenous family languages	0
Enhanced Vegetation Index (EVI)	0
Height above the nearest drainage (HAND)	0
Soil fertility	0
Annual Gross Primary Productivity (GPP)	0
Current distribution of family languages	0
Current distribution of Indigenous and non-indigenous peoples	0
Proportion of habitat loss	0
Urban-rural catchment areas (URCA)	0
r2	-0.06

Falconidae

Historical distribution of Indigenous family languages	0.00010
Elevation	0.00006
Annual Net Primary Productivity (NPP)	0.00005
Current distribution of family languages	0.00005
Annual Gross Primary Productivity (GPP)	0.00005
Urban-rural catchment areas (URCA)	0.00004
Proportion of flooded areas	0.00004
Soil fertility	0.00003
Proportion of habitat loss	0.00002
Enhanced Vegetation Index (EVI)	0.00002
Height above the nearest drainage (HAND)	0.00001
Current distribution of Indigenous and non-indigenous peoples	0.00001
r2	0.126

Galea

Soil fertility	0.00111
Proportion of flooded areas	0.00049
Annual Gross Primary Productivity (GPP)	0.00038
Elevation	0.00036
Height above the nearest drainage (HAND)	0.00034
Enhanced Vegetation Index (EVI)	0.00022
Proportion of habitat loss	0.00018
Annual Net Primary Productivity (NPP)	0.00016
Current distribution of family languages	0.00009
Historical distribution of Indigenous family languages	0.00004
Urban-rural catchment areas (URCA)	0.00003
Current distribution of Indigenous and non-indigenous peoples	0.00002
r2	0.359

Galictis vittata

Soil fertility	0
Annual Gross Primary Productivity (GPP)	0
Annual Net Primary Productivity (NPP)	0
Proportion of habitat loss	0
Historical distribution of Indigenous family languages	0
Proportion of flooded areas	0
Enhanced Vegetation Index (EVI)	0
Urban-rural catchment areas (URCA)	0
Elevation	0
Height above the nearest drainage (HAND)	0
Current distribution of Indigenous and non-indigenous peoples	0
Current distribution of family languages	0
r2	-0.208

Harpia harpyja

Elevation	0.00011
Proportion of habitat loss	0.00008
Proportion of flooded areas	0.00006
Annual Net Primary Productivity (NPP)	0.00005
Soil fertility	0.00004
Annual Gross Primary Productivity (GPP)	0.00003
Current distribution of family languages	0.00003
Historical distribution of Indigenous family languages	0.00002
Enhanced Vegetation Index (EVI)	0.00002
Height above the nearest drainage (HAND)	0.00002
Urban-rural catchment areas (URCA)	0.00001
Current distribution of Indigenous and non-indigenous peoples	0.00000
r2	-0.266

Heliornis fulica

Soil fertility	0.00191
Height above the nearest drainage (HAND)	0.00039
Annual Gross Primary Productivity (GPP)	0.00035
Annual Net Primary Productivity (NPP)	0.00035
Proportion of flooded areas	0.00033
Enhanced Vegetation Index (EVI)	0.00033
Elevation	0.00029
Current distribution of family languages	0.00021
Historical distribution of Indigenous family languages	0.00010
Current distribution of Indigenous and non-indigenous peoples	0.00001
Urban-rural catchment areas (URCA)	0.00000
Proportion of habitat loss	0.00000
r2	0.569

Herpailurus yagouaroundi

Urban-rural catchment areas (URCA)	0.00012
Proportion of habitat loss	0.00012
Annual Gross Primary Productivity (GPP)	0.00006
Height above the nearest drainage (HAND)	0.00005
Enhanced Vegetation Index (EVI)	0.00005
Elevation	0.00003
Proportion of flooded areas	0.00003
Annual Net Primary Productivity (NPP)	0.00003
Soil fertility	0.00002
Historical distribution of Indigenous family languages	0.00002
Current distribution of Indigenous and non-indigenous peoples	0.00000
Current distribution of family languages	0.00000
r2	0.023

Hydrochoerus hydrochaeris

Elevation	0.18687
Urban-rural catchment areas (URCA)	0.18132
Height above the nearest drainage (HAND)	0.17925
Annual Gross Primary Productivity (GPP)	0.13388
Enhanced Vegetation Index (EVI)	0.13297
Annual Net Primary Productivity (NPP)	0.10754
Proportion of habitat loss	0.09853
Historical distribution of Indigenous family languages	0.08103
Proportion of flooded areas	0.07764
Soil fertility	0.06034
Current distribution of family languages	0.00485
Current distribution of Indigenous and non-indigenous peoples	0.00247
r2	-0.036

Iguana iguana

Elevation	0.03026
Height above the nearest drainage (HAND)	0.02129
Urban-rural catchment areas (URCA)	0.01362
Enhanced Vegetation Index (EVI)	0.01310
Annual Net Primary Productivity (NPP)	0.01227
Annual Gross Primary Productivity (GPP)	0.01142
Proportion of flooded areas	0.01003
Soil fertility	0.00691
Historical distribution of Indigenous family languages	0.00617
Proportion of habitat loss	0.00586
Current distribution of family languages	0.00465
Current distribution of Indigenous and non-indigenous peoples	0.00038
r2	-0.014

Inia geoffrensis

Annual Net Primary Productivity (NPP)	0.00003
Enhanced Vegetation Index (EVI)	0.00003
Annual Gross Primary Productivity (GPP)	0.00003
Elevation	0.00003
Soil fertility	0.00003
Proportion of flooded areas	0.00002
Height above the nearest drainage (HAND)	0.00002
Historical distribution of Indigenous family languages	0.00001
Proportion of habitat loss	0.00000
Current distribution of family languages	0.00000
Urban-rural catchment areas (URCA)	0.00000
Current distribution of Indigenous and non-indigenous peoples	0.00000
r2	-0.038

Jabiru mycteria

Historical distribution of Indigenous family languages	0.00009
Elevation	0.00004
Soil fertility	0.00003
Enhanced Vegetation Index (EVI)	0.00003
Annual Net Primary Productivity (NPP)	0.00002
Proportion of flooded areas	0.00002
Annual Gross Primary Productivity (GPP)	0.00002
Height above the nearest drainage (HAND)	0.00001
Proportion of habitat loss	0.00000
Current distribution of family languages	0.00000
Urban-rural catchment areas (URCA)	0.00000
Current distribution of Indigenous and non-indigenous peoples	0.00000
r2	-0.057

Jacana jacana

Urban-rural catchment areas (URCA)	0.00007
Height above the nearest drainage (HAND)	0.00006
Enhanced Vegetation Index (EVI)	0.00005
Annual Net Primary Productivity (NPP)	0.00003
Elevation	0.00003
Proportion of habitat loss	0.00003
Proportion of flooded areas	0.00002
Soil fertility	0.00002
Annual Gross Primary Productivity (GPP)	0.00002
Historical distribution of Indigenous family languages	0.00002
Current distribution of family languages	0.00000
Current distribution of Indigenous and non-indigenous peoples	0.00000
r2	-0.259

Kinosternon scorpioides

Annual Net Primary Productivity (NPP)	0.00013
Annual Gross Primary Productivity (GPP)	0.00013
Soil fertility	0.00002
Urban-rural catchment areas (URCA)	0.00001
Height above the nearest drainage (HAND)	0.00001
Current distribution of family languages	0.00001
Elevation	0.00001
Proportion of flooded areas	0.00000
Enhanced Vegetation Index (EVI)	0.00000
Proportion of habitat loss	0.00000
Current distribution of Indigenous and non-indigenous peoples	0.00000
Historical distribution of Indigenous family languages	0.00000
r2	-0.054

Lacertilia (others)

Annual Net Primary Productivity (NPP)	0.00003
Annual Gross Primary Productivity (GPP)	0.00002
Soil fertility	0.00002
Current distribution of family languages	0.00001
Enhanced Vegetation Index (EVI)	0.00001
Height above the nearest drainage (HAND)	0.00001
Elevation	0.00000
Historical distribution of Indigenous family languages	0.00000
Proportion of flooded areas	0.00000
Proportion of habitat loss	0.00000
Urban-rural catchment areas (URCA)	0.00000
Current distribution of Indigenous and non-indigenous peoples	0.00000
r2	-0.188

Lagothrix

Historical distribution of Indigenous family languages	0.12597
Annual Gross Primary Productivity (GPP)	0.08770
Current distribution of family languages	0.08504
Annual Net Primary Productivity (NPP)	0.05708
Height above the nearest drainage (HAND)	0.04092
Elevation	0.04057
Enhanced Vegetation Index (EVI)	0.03918
Soil fertility	0.03694
Proportion of flooded areas	0.02676
Proportion of habitat loss	0.01833
Urban-rural catchment areas (URCA)	0.01805
Current distribution of Indigenous and non-indigenous peoples	0.00363
r2	0.344

Leontocebus

Enhanced Vegetation Index (EVI)	0.00178
Current distribution of family languages	0.00112
Annual Net Primary Productivity (NPP)	0.00091
Height above the nearest drainage (HAND)	0.00085
Elevation	0.00079
Proportion of habitat loss	0.00078
Annual Gross Primary Productivity (GPP)	0.00074
Historical distribution of Indigenous family languages	0.00057
Soil fertility	0.00052
Proportion of flooded areas	0.00021
Current distribution of Indigenous and non-indigenous peoples	0.00009
Urban-rural catchment areas (URCA)	0.00007
r2	-0.186

Leopardus pardalis

Annual Gross Primary Productivity (GPP)	0.00154
Proportion of habitat loss	0.00153
Height above the nearest drainage (HAND)	0.00151
Historical distribution of Indigenous family languages	0.00116
Annual Net Primary Productivity (NPP)	0.00115
Elevation	0.00113
Soil fertility	0.00096
Urban-rural catchment areas (URCA)	0.00086
Enhanced Vegetation Index (EVI)	0.00079
Proportion of flooded areas	0.00062
Current distribution of family languages	0.00060
Current distribution of Indigenous and non-indigenous peoples	0.00011
r2	-0.108

Leopardus wiedii

Proportion of flooded areas	0.00017
Proportion of habitat loss	0.00015
Height above the nearest drainage (HAND)	0.00014
Elevation	0.00012
Annual Gross Primary Productivity (GPP)	0.00011
Urban-rural catchment areas (URCA)	0.00011
Enhanced Vegetation Index (EVI)	0.00010
Historical distribution of Indigenous family languages	0.00007
Soil fertility	0.00007
Annual Net Primary Productivity (NPP)	0.00006
Current distribution of family languages	0.00000
Current distribution of Indigenous and non-indigenous peoples	0.00000
r2	-0.047

Lontra longicaudis

Proportion of habitat loss	0.00017
Elevation	0.00011
Historical distribution of Indigenous family languages	0.00011
Annual Gross Primary Productivity (GPP)	0.00009
Annual Net Primary Productivity (NPP)	0.00005
Urban-rural catchment areas (URCA)	0.00005
Soil fertility	0.00005
Enhanced Vegetation Index (EVI)	0.00004
Height above the nearest drainage (HAND)	0.00002
Proportion of flooded areas	0.00002
Current distribution of family languages	0.00000
Current distribution of Indigenous and non-indigenous peoples	0.00000
r2	-0.009

Mazama americana

Historical distribution of Indigenous family languages	0.19630
Soil fertility	0.16513
Height above the nearest drainage (HAND)	0.13734
Elevation	0.12827
Enhanced Vegetation Index (EVI)	0.12046
Annual Gross Primary Productivity (GPP)	0.09241
Annual Net Primary Productivity (NPP)	0.08599
Proportion of flooded areas	0.06901
Current distribution of family languages	0.06528
Proportion of habitat loss	0.05530
Urban-rural catchment areas (URCA)	0.04969
Current distribution of Indigenous and non-indigenous peoples	0.00825
r2	0.187

Mazama gouazoubira

Elevation	0.00005
Soil fertility	0.00003
Height above the nearest drainage (HAND)	0.00003
Enhanced Vegetation Index (EVI)	0.00002
Proportion of habitat loss	0.00002
Proportion of flooded areas	0.00002
Historical distribution of Indigenous family languages	0.00002
Urban-rural catchment areas (URCA)	0.00002
Annual Net Primary Productivity (NPP)	0.00001
Current distribution of family languages	0.00001
Annual Gross Primary Productivity (GPP)	0.00000
Current distribution of Indigenous and non-indigenous peoples	0.00000
r2	-0.212

Mazama nemorivaga

Historical distribution of Indigenous family languages	0.19912
Soil fertility	0.16597
Height above the nearest drainage (HAND)	0.13324
Elevation	0.12999
Enhanced Vegetation Index (EVI)	0.12534
Annual Gross Primary Productivity (GPP)	0.08981
Annual Net Primary Productivity (NPP)	0.08676
Proportion of flooded areas	0.07280
Current distribution of family languages	0.05797
Proportion of habitat loss	0.05463
Urban-rural catchment areas (URCA)	0.04876
Current distribution of Indigenous and non-indigenous peoples	0.00769
r2	0.186

Melanosuchus niger

Enhanced Vegetation Index (EVI)	0.03935
Elevation	0.03469
Soil fertility	0.03192
Proportion of flooded areas	0.01793
Historical distribution of Indigenous family languages	0.01776
Annual Net Primary Productivity (NPP)	0.01594
Height above the nearest drainage (HAND)	0.01264
Annual Gross Primary Productivity (GPP)	0.00982
Proportion of habitat loss	0.00333
Urban-rural catchment areas (URCA)	0.00257
Current distribution of family languages	0.00210
Current distribution of Indigenous and non-indigenous peoples	0.00020
r2	0.006

Mesembrinibis cayennensis

Annual Net Primary Productivity (NPP)	0.00361
Annual Gross Primary Productivity (GPP)	0.00243
Historical distribution of Indigenous family languages	0.00188
Elevation	0.00117
Soil fertility	0.00101
Enhanced Vegetation Index (EVI)	0.00093
Height above the nearest drainage (HAND)	0.00065
Proportion of flooded areas	0.00052
Proportion of habitat loss	0.00019
Current distribution of Indigenous and non-indigenous peoples	0.00012
Current distribution of family languages	0.00008
Urban-rural catchment areas (URCA)	0.00008
r2	-0.331

Mesoclemmys

Annual Gross Primary Productivity (GPP)	0.00020
Elevation	0.00020
Soil fertility	0.00017
Enhanced Vegetation Index (EVI)	0.00016
Annual Net Primary Productivity (NPP)	0.00015
Height above the nearest drainage (HAND)	0.00015
Proportion of flooded areas	0.00011
Historical distribution of Indigenous family languages	0.00010
Current distribution of family languages	0.00007
Proportion of habitat loss	0.00004
Urban-rural catchment areas (URCA)	0.00002
Current distribution of Indigenous and non-indigenous peoples	0.00001
r2	-0.045

Mico

Soil fertility	0
Annual Net Primary Productivity (NPP)	0
Annual Gross Primary Productivity (GPP)	0
Proportion of flooded areas	0
Elevation	0
Enhanced Vegetation Index (EVI)	0
Urban-rural catchment areas (URCA)	0
Current distribution of family languages	0
Height above the nearest drainage (HAND)	0
Proportion of habitat loss	0
Historical distribution of Indigenous family languages	0
Current distribution of Indigenous and non-indigenous peoples	0
r2	-0.025

Microsciurus flaviventer

Historical distribution of Indigenous family languages	0.00004
Elevation	0.00001
Annual Net Primary Productivity (NPP)	0.00001
Soil fertility	0.00000
Annual Gross Primary Productivity (GPP)	0.00000
Enhanced Vegetation Index (EVI)	0.00000
Proportion of flooded areas	0.00000
Height above the nearest drainage (HAND)	0.00000
Proportion of habitat loss	0.00000
Current distribution of family languages	0.00000
Current distribution of Indigenous and non-indigenous peoples	0.00000
Urban-rural catchment areas (URCA)	0.00000
r2	-0.016

Mitu

Historical distribution of Indigenous family languages	0.15793
Soil fertility	0.10840
Elevation	0.08981
Proportion of habitat loss	0.08797
Annual Gross Primary Productivity (GPP)	0.07928
Annual Net Primary Productivity (NPP)	0.07754
Enhanced Vegetation Index (EVI)	0.07466
Height above the nearest drainage (HAND)	0.07376
Current distribution of family languages	0.06890
Proportion of flooded areas	0.06494
Urban-rural catchment areas (URCA)	0.04244
Current distribution of Indigenous and non-indigenous peoples	0.00495
r2	-0.034

Momotidae

Current distribution of family languages	0.00004
Historical distribution of Indigenous family languages	0.00002
Height above the nearest drainage (HAND)	0.00001
Soil fertility	0.00000
Annual Net Primary Productivity (NPP)	0.00000
Annual Gross Primary Productivity (GPP)	0.00000
Elevation	0.00000
Enhanced Vegetation Index (EVI)	0.00000
Proportion of flooded areas	0.00000
Urban-rural catchment areas (URCA)	0.00000
Current distribution of Indigenous and non-indigenous peoples	0.00000
Proportion of habitat loss	0.00000
r2	-0.047

Mycteria americana

Elevation	0.00004
Enhanced Vegetation Index (EVI)	0.00003
Annual Net Primary Productivity (NPP)	0.00002
Annual Gross Primary Productivity (GPP)	0.00001
Soil fertility	0.00001
Height above the nearest drainage (HAND)	0.00001
Proportion of flooded areas	0.00000
Historical distribution of Indigenous family languages	0.00000
Current distribution of family languages	0.00000
Proportion of habitat loss	0.00000
Urban-rural catchment areas (URCA)	0.00000
Current distribution of Indigenous and non-indigenous peoples	0.00000
r2	-0.051

Myoprocta

Historical distribution of Indigenous family languages	0.03739
Current distribution of family languages	0.02742
Elevation	0.02394
Soil fertility	0.02318
Enhanced Vegetation Index (EVI)	0.01880
Height above the nearest drainage (HAND)	0.01876
Annual Net Primary Productivity (NPP)	0.01484
Annual Gross Primary Productivity (GPP)	0.01143
Proportion of flooded areas	0.01008
Proportion of habitat loss	0.00884
Urban-rural catchment areas (URCA)	0.00723
Current distribution of Indigenous and non-indigenous peoples	0.00226
r2	0.26

Myrmecophaga tridactyla

Soil fertility	0.00149
Current distribution of family languages	0.00120
Historical distribution of Indigenous family languages	0.00099
Height above the nearest drainage (HAND)	0.00092
Annual Net Primary Productivity (NPP)	0.00088
Annual Gross Primary Productivity (GPP)	0.00085
Enhanced Vegetation Index (EVI)	0.00083
Elevation	0.00082
Proportion of flooded areas	0.00050
Proportion of habitat loss	0.00032
Urban-rural catchment areas (URCA)	0.00018
Current distribution of Indigenous and non-indigenous peoples	0.00006
r2	-0.155

Nannopterum brasilianus

Historical distribution of Indigenous family languages	0.34538
Enhanced Vegetation Index (EVI)	0.10022
Elevation	0.08224
Soil fertility	0.04113
Annual Net Primary Productivity (NPP)	0.03324
Height above the nearest drainage (HAND)	0.02406
Urban-rural catchment areas (URCA)	0.02216
Proportion of habitat loss	0.01371
Proportion of flooded areas	0.00903
Annual Gross Primary Productivity (GPP)	0.00706
Current distribution of family languages	0.00480
Current distribution of Indigenous and non-indigenous peoples	0.00041
r2	0.001

Nasua nasua

Historical distribution of Indigenous family languages	0.05054
Current distribution of family languages	0.04546
Elevation	0.04035
Annual Gross Primary Productivity (GPP)	0.03879
Soil fertility	0.03592
Annual Net Primary Productivity (NPP)	0.03097
Height above the nearest drainage (HAND)	0.02978
Enhanced Vegetation Index (EVI)	0.02601
Proportion of flooded areas	0.01635
Urban-rural catchment areas (URCA)	0.01327
Proportion of habitat loss	0.01057
Current distribution of Indigenous and non-indigenous peoples	0.00329
r2	0.216

Nasuella olivacea

Annual Net Primary Productivity (NPP)	0
Annual Gross Primary Productivity (GPP)	0
Enhanced Vegetation Index (EVI)	0
Soil fertility	0
Elevation	0
Height above the nearest drainage (HAND)	0
Proportion of flooded areas	0
Proportion of habitat loss	0
Urban-rural catchment areas (URCA)	0
Historical distribution of Indigenous family languages	0
Current distribution of Indigenous and non-indigenous peoples	0
Current distribution of family languages	0
r2	NaN

Neochen jubata

Elevation	0.00011
Height above the nearest drainage (HAND)	0.00009
Enhanced Vegetation Index (EVI)	0.00007
Historical distribution of Indigenous family languages	0.00005
Current distribution of family languages	0.00005
Annual Net Primary Productivity (NPP)	0.00004
Annual Gross Primary Productivity (GPP)	0.00004
Soil fertility	0.00004
Proportion of flooded areas	0.00003
Proportion of habitat loss	0.00001
Current distribution of Indigenous and non-indigenous peoples	0.00001
Urban-rural catchment areas (URCA)	0.00000
r2	-0.151

Nothocrax urumutum

Historical distribution of Indigenous family languages	0.00127
Current distribution of family languages	0.00112
Elevation	0.00101
Annual Net Primary Productivity (NPP)	0.00083
Proportion of habitat loss	0.00036
Soil fertility	0.00035
Enhanced Vegetation Index (EVI)	0.00031
Proportion of flooded areas	0.00030
Annual Gross Primary Productivity (GPP)	0.00028
Height above the nearest drainage (HAND)	0.00022
Urban-rural catchment areas (URCA)	0.00006
Current distribution of Indigenous and non-indigenous peoples	0.00003
r2	-0.095

Odocoileus virginianus

Current distribution of family languages	0.00137
Height above the nearest drainage (HAND)	0.00063
Annual Net Primary Productivity (NPP)	0.00063
Annual Gross Primary Productivity (GPP)	0.00045
Elevation	0.00037
Enhanced Vegetation Index (EVI)	0.00024
Proportion of flooded areas	0.00014
Historical distribution of Indigenous family languages	0.00014
Soil fertility	0.00010
Proportion of habitat loss	0.00006
Urban-rural catchment areas (URCA)	0.00003
Current distribution of Indigenous and non-indigenous peoples	0.00002
r2	-0.097

Odontophorus

Elevation	0.00228
Annual Net Primary Productivity (NPP)	0.00171
Soil fertility	0.00166
Historical distribution of Indigenous family languages	0.00143
Current distribution of family languages	0.00104
Annual Gross Primary Productivity (GPP)	0.00101
Height above the nearest drainage (HAND)	0.00072
Enhanced Vegetation Index (EVI)	0.00049
Proportion of flooded areas	0.00045
Proportion of habitat loss	0.00017
Urban-rural catchment areas (URCA)	0.00012
Current distribution of Indigenous and non-indigenous peoples	0.00006
r2	-0.047

Opisthocomus hoazin

Proportion of flooded areas	0.00712
Annual Gross Primary Productivity (GPP)	0.00551
Elevation	0.00541
Height above the nearest drainage (HAND)	0.00486
Annual Net Primary Productivity (NPP)	0.00360
Proportion of habitat loss	0.00335
Enhanced Vegetation Index (EVI)	0.00228
Soil fertility	0.00219
Historical distribution of Indigenous family languages	0.00156
Current distribution of Indigenous and non-indigenous peoples	0.00078
Urban-rural catchment areas (URCA)	0.00056
Current distribution of family languages	0.00001
r2	-0.033

Ortalis

Current distribution of family languages	0.01910
Height above the nearest drainage (HAND)	0.01848
Elevation	0.01667
Historical distribution of Indigenous family languages	0.01568
Annual Gross Primary Productivity (GPP)	0.01075
Enhanced Vegetation Index (EVI)	0.00936
Proportion of flooded areas	0.00872
Soil fertility	0.00604
Annual Net Primary Productivity (NPP)	0.00422
Proportion of habitat loss	0.00219
Urban-rural catchment areas (URCA)	0.00198
Current distribution of Indigenous and non-indigenous peoples	0.00056
r2	-0.045

Ozotoceros bezoarticus

Current distribution of family languages	0
Current distribution of Indigenous and non-indigenous peoples	0
Annual Gross Primary Productivity (GPP)	0
Soil fertility	0
Proportion of flooded areas	0
Proportion of habitat loss	0
Elevation	0
Height above the nearest drainage (HAND)	0
Annual Net Primary Productivity (NPP)	0
Enhanced Vegetation Index (EVI)	0
Urban-rural catchment areas (URCA)	0
Historical distribution of Indigenous family languages	0
r2	-3

Paleosuchus palpebrosus

Current distribution of family languages	0.00545
Historical distribution of Indigenous family languages	0.00392
Annual Net Primary Productivity (NPP)	0.00382
Height above the nearest drainage (HAND)	0.00365
Enhanced Vegetation Index (EVI)	0.00338
Elevation	0.00324
Soil fertility	0.00289
Annual Gross Primary Productivity (GPP)	0.00279
Proportion of flooded areas	0.00184
Urban-rural catchment areas (URCA)	0.00036
Current distribution of Indigenous and non-indigenous peoples	0.00022
Proportion of habitat loss	0.00015
r2	-0.082

Paleosuchus trigonatus

Soil fertility	0.04157
Current distribution of family languages	0.02122
Height above the nearest drainage (HAND)	0.01463
Elevation	0.01152
Annual Net Primary Productivity (NPP)	0.01059
Enhanced Vegetation Index (EVI)	0.00935
Annual Gross Primary Productivity (GPP)	0.00922
Proportion of flooded areas	0.00809
Historical distribution of Indigenous family languages	0.00783
Proportion of habitat loss	0.00563
Urban-rural catchment areas (URCA)	0.00367
Current distribution of Indigenous and non-indigenous peoples	0.00069
r2	0.16

Panthera onca

Annual Net Primary Productivity (NPP)	0.00430
Height above the nearest drainage (HAND)	0.00300
Urban-rural catchment areas (URCA)	0.00241
Proportion of habitat loss	0.00232
Annual Gross Primary Productivity (GPP)	0.00232
Elevation	0.00230
Proportion of flooded areas	0.00218
Enhanced Vegetation Index (EVI)	0.00215
Soil fertility	0.00211
Historical distribution of Indigenous family languages	0.00168
Current distribution of family languages	0.00022
Current distribution of Indigenous and non-indigenous peoples	0.00003
r2	-0.074

Passeriformes (others)

Current distribution of family languages	0.01931
Historical distribution of Indigenous family languages	0.00625
Soil fertility	0.00483
Elevation	0.00474
Annual Net Primary Productivity (NPP)	0.00398
Urban-rural catchment areas (URCA)	0.00339
Height above the nearest drainage (HAND)	0.00253
Annual Gross Primary Productivity (GPP)	0.00245
Proportion of flooded areas	0.00217
Proportion of habitat loss	0.00110
Enhanced Vegetation Index (EVI)	0.00072
Current distribution of Indigenous and non-indigenous peoples	0.00010
r2	-0.101

Patagioenas

Proportion of habitat loss	0.01391
Height above the nearest drainage (HAND)	0.01188
Annual Net Primary Productivity (NPP)	0.00808
Urban-rural catchment areas (URCA)	0.00761
Annual Gross Primary Productivity (GPP)	0.00652
Proportion of flooded areas	0.00528
Elevation	0.00503
Enhanced Vegetation Index (EVI)	0.00442
Historical distribution of Indigenous family languages	0.00272
Soil fertility	0.00226
Current distribution of family languages	0.00080
Current distribution of Indigenous and non-indigenous peoples	0.00008
r2	-0.018

Peltocephalus dumerilianus

Annual Net Primary Productivity (NPP)	0.18489
Annual Gross Primary Productivity (GPP)	0.17846
Elevation	0.15025
Proportion of flooded areas	0.10436
Soil fertility	0.09401
Enhanced Vegetation Index (EVI)	0.08137
Historical distribution of Indigenous family languages	0.07136
Height above the nearest drainage (HAND)	0.07020
Proportion of habitat loss	0.01637
Urban-rural catchment areas (URCA)	0.01128
Current distribution of Indigenous and non-indigenous peoples	0.00771
Current distribution of family languages	0.00593
r2	0.571

Penelope

Proportion of habitat loss	0.25468
Historical distribution of Indigenous family languages	0.23099
Height above the nearest drainage (HAND)	0.21191
Elevation	0.19455
Enhanced Vegetation Index (EVI)	0.17033
Annual Net Primary Productivity (NPP)	0.15783
Soil fertility	0.14396
Urban-rural catchment areas (URCA)	0.13798
Annual Gross Primary Productivity (GPP)	0.13725
Proportion of flooded areas	0.11811
Current distribution of family languages	0.08648
Current distribution of Indigenous and non-indigenous peoples	0.01241
r2	0.044

Phoenicoparrus

Soil fertility	0
Annual Net Primary Productivity (NPP)	0
Elevation	0
Proportion of flooded areas	0
Enhanced Vegetation Index (EVI)	0
Height above the nearest drainage (HAND)	0
Annual Gross Primary Productivity (GPP)	0
Historical distribution of Indigenous family languages	0
Current distribution of family languages	0
Proportion of habitat loss	0
Current distribution of Indigenous and non-indigenous peoples	0
Urban-rural catchment areas (URCA)	0
r2	-0.095

Phrynops

Annual Net Primary Productivity (NPP)	0.00005
Annual Gross Primary Productivity (GPP)	0.00004
Soil fertility	0.00003
Elevation	0.00002
Height above the nearest drainage (HAND)	0.00001
Enhanced Vegetation Index (EVI)	0.00001
Current distribution of family languages	0.00001
Proportion of flooded areas	0.00001
Historical distribution of Indigenous family languages	0.00001
Urban-rural catchment areas (URCA)	0.00000
Proportion of habitat loss	0.00000
Current distribution of Indigenous and non-indigenous peoples	0.00000
r2	-0.119

Picidae

Historical distribution of Indigenous family languages	0.00014
Current distribution of family languages	0.00006
Soil fertility	0.00006
Elevation	0.00006
Annual Net Primary Productivity (NPP)	0.00006
Annual Gross Primary Productivity (GPP)	0.00005
Urban-rural catchment areas (URCA)	0.00004
Height above the nearest drainage (HAND)	0.00003
Proportion of habitat loss	0.00003
Enhanced Vegetation Index (EVI)	0.00003
Proportion of flooded areas	0.00002
Current distribution of Indigenous and non-indigenous peoples	0.00001
r2	-0.253

Pionus

Soil fertility	0.00114
Historical distribution of Indigenous family languages	0.00103
Elevation	0.00038
Annual Net Primary Productivity (NPP)	0.00034
Enhanced Vegetation Index (EVI)	0.00028
Annual Gross Primary Productivity (GPP)	0.00025
Proportion of habitat loss	0.00021
Current distribution of family languages	0.00017
Proportion of flooded areas	0.00016
Height above the nearest drainage (HAND)	0.00013
Urban-rural catchment areas (URCA)	0.00012
Current distribution of Indigenous and non-indigenous peoples	0.00008
r2	0.521

Pipile

Current distribution of family languages	0.04954
Historical distribution of Indigenous family languages	0.04377
Elevation	0.02509
Proportion of flooded areas	0.01875
Proportion of habitat loss	0.01824
Soil fertility	0.01647
Height above the nearest drainage (HAND)	0.01600
Enhanced Vegetation Index (EVI)	0.01350
Urban-rural catchment areas (URCA)	0.01304
Annual Net Primary Productivity (NPP)	0.01272
Annual Gross Primary Productivity (GPP)	0.01163
Current distribution of Indigenous and non-indigenous peoples	0.00095
r2	0.027

Pithecia

Historical distribution of Indigenous family languages	0.00921
Enhanced Vegetation Index (EVI)	0.00818
Annual Gross Primary Productivity (GPP)	0.00638
Annual Net Primary Productivity (NPP)	0.00556
Proportion of habitat loss	0.00540
Height above the nearest drainage (HAND)	0.00538
Soil fertility	0.00529
Elevation	0.00478
Proportion of flooded areas	0.00440
Current distribution of family languages	0.00352
Urban-rural catchment areas (URCA)	0.00247
Current distribution of Indigenous and non-indigenous peoples	0.00066
r2	0.057

Platemys platycephala

Soil fertility	0.00119
Historical distribution of Indigenous family languages	0.00023
Annual Net Primary Productivity (NPP)	0.00023
Current distribution of family languages	0.00022
Elevation	0.00019
Enhanced Vegetation Index (EVI)	0.00015
Annual Gross Primary Productivity (GPP)	0.00014
Proportion of flooded areas	0.00008
Height above the nearest drainage (HAND)	0.00004
Proportion of habitat loss	0.00001
Current distribution of Indigenous and non-indigenous peoples	0.00001
Urban-rural catchment areas (URCA)	0.00001
r2	-0.077

Plecturocebus

Soil fertility	0.01307
Historical distribution of Indigenous family languages	0.01241
Current distribution of family languages	0.00609
Annual Net Primary Productivity (NPP)	0.00546
Enhanced Vegetation Index (EVI)	0.00358
Elevation	0.00343
Annual Gross Primary Productivity (GPP)	0.00299
Height above the nearest drainage (HAND)	0.00291
Proportion of flooded areas	0.00232
Proportion of habitat loss	0.00173
Current distribution of Indigenous and non-indigenous peoples	0.00038
Urban-rural catchment areas (URCA)	0.00027
r2	-0.16

Podocnemis erythrocephala

Elevation	0.28702
Annual Gross Primary Productivity (GPP)	0.24637
Proportion of flooded areas	0.24067
Soil fertility	0.20006
Height above the nearest drainage (HAND)	0.15381
Annual Net Primary Productivity (NPP)	0.11004
Enhanced Vegetation Index (EVI)	0.09917
Historical distribution of Indigenous family languages	0.04980
Current distribution of Indigenous and non-indigenous peoples	0.03628
Current distribution of family languages	0.03562
Proportion of habitat loss	0.00549
Urban-rural catchment areas (URCA)	0.00535
r2	0.546

Podocnemis expansa

Elevation	0.06471
Annual Net Primary Productivity (NPP)	0.06052
Current distribution of family languages	0.05695
Annual Gross Primary Productivity (GPP)	0.05676
Proportion of habitat loss	0.05619
Historical distribution of Indigenous family languages	0.05513
Soil fertility	0.05393
Height above the nearest drainage (HAND)	0.05264
Enhanced Vegetation Index (EVI)	0.04224
Proportion of flooded areas	0.04141
Urban-rural catchment areas (URCA)	0.01499
Current distribution of Indigenous and non-indigenous peoples	0.01006
r2	-0.057

Podocnemis sextuberculata

Historical distribution of Indigenous family languages	0.17690
Proportion of flooded areas	0.15674
Soil fertility	0.14410
Elevation	0.11556
Height above the nearest drainage (HAND)	0.11508
Enhanced Vegetation Index (EVI)	0.10291
Annual Net Primary Productivity (NPP)	0.06372
Annual Gross Primary Productivity (GPP)	0.05408
Proportion of habitat loss	0.03998
Current distribution of family languages	0.01444
Current distribution of Indigenous and non-indigenous peoples	0.00373
Urban-rural catchment areas (URCA)	0.00344
r2	0.299

Podocnemis unifilis

Elevation	1.40543
Current distribution of family languages	0.81754
Enhanced Vegetation Index (EVI)	0.66461
Proportion of flooded areas	0.54477
Annual Net Primary Productivity (NPP)	0.42633
Annual Gross Primary Productivity (GPP)	0.38175
Soil fertility	0.37221
Height above the nearest drainage (HAND)	0.28169
Historical distribution of Indigenous family languages	0.23138
Proportion of habitat loss	0.10332
Urban-rural catchment areas (URCA)	0.08331
Current distribution of Indigenous and non-indigenous peoples	0.07573
r2	0.571

Podocnemis vogli

Annual Net Primary Productivity (NPP)	0.00035
Soil fertility	0.00031
Proportion of habitat loss	0.00025
Elevation	0.00023
Annual Gross Primary Productivity (GPP)	0.00022
Enhanced Vegetation Index (EVI)	0.00020
Height above the nearest drainage (HAND)	0.00009
Historical distribution of Indigenous family languages	0.00007
Proportion of flooded areas	0.00001
Urban-rural catchment areas (URCA)	0.00000
Current distribution of family languages	0.00000
Current distribution of Indigenous and non-indigenous peoples	0.00000
r2	-0.091

Potos flavus

Annual Gross Primary Productivity (GPP)	0.00652
Historical distribution of Indigenous family languages	0.00408
Elevation	0.00363
Soil fertility	0.00330
Height above the nearest drainage (HAND)	0.00308
Annual Net Primary Productivity (NPP)	0.00258
Enhanced Vegetation Index (EVI)	0.00213
Current distribution of family languages	0.00195
Proportion of flooded areas	0.00149
Proportion of habitat loss	0.00146
Urban-rural catchment areas (URCA)	0.00096
Current distribution of Indigenous and non-indigenous peoples	0.00040
r2	0.082

Priodontes maximus

Enhanced Vegetation Index (EVI)	0.01984
Height above the nearest drainage (HAND)	0.01922
Elevation	0.00897
Urban-rural catchment areas (URCA)	0.00636
Proportion of habitat loss	0.00621
Proportion of flooded areas	0.00597
Annual Net Primary Productivity (NPP)	0.00512
Annual Gross Primary Productivity (GPP)	0.00501
Soil fertility	0.00486
Historical distribution of Indigenous family languages	0.00304
Current distribution of family languages	0.00023
Current distribution of Indigenous and non-indigenous peoples	0.00014
r2	-0.059

Procyon cancrivorus

Historical distribution of Indigenous family languages	0
Current distribution of family languages	0
Soil fertility	0
Annual Gross Primary Productivity (GPP)	0
Annual Net Primary Productivity (NPP)	0
Proportion of flooded areas	0
Proportion of habitat loss	0
Elevation	0
Height above the nearest drainage (HAND)	0
Enhanced Vegetation Index (EVI)	0
Urban-rural catchment areas (URCA)	0
Current distribution of Indigenous and non-indigenous peoples	0
r2	-0.057

Psarocolius

Current distribution of family languages	0.00050
Historical distribution of Indigenous family languages	0.00019
Annual Net Primary Productivity (NPP)	0.00016
Annual Gross Primary Productivity (GPP)	0.00013
Elevation	0.00011
Soil fertility	0.00008
Enhanced Vegetation Index (EVI)	0.00007
Height above the nearest drainage (HAND)	0.00005
Proportion of flooded areas	0.00004
Proportion of habitat loss	0.00002
Urban-rural catchment areas (URCA)	0.00001
Current distribution of Indigenous and non-indigenous peoples	0.00001
r2	0.003

Psittacidae (others)

Historical distribution of Indigenous family languages	0.00726
Height above the nearest drainage (HAND)	0.00686
Proportion of flooded areas	0.00645
Current distribution of family languages	0.00430
Soil fertility	0.00379
Elevation	0.00340
Annual Gross Primary Productivity (GPP)	0.00331
Annual Net Primary Productivity (NPP)	0.00313
Enhanced Vegetation Index (EVI)	0.00253
Proportion of habitat loss	0.00083
Urban-rural catchment areas (URCA)	0.00076
Current distribution of Indigenous and non-indigenous peoples	0.00013
r2	-0.198

Psophia

Annual Net Primary Productivity (NPP)	0.02925
Historical distribution of Indigenous family languages	0.02583
Annual Gross Primary Productivity (GPP)	0.02120
Current distribution of family languages	0.01716
Soil fertility	0.01634
Elevation	0.01487
Height above the nearest drainage (HAND)	0.01451
Enhanced Vegetation Index (EVI)	0.01380
Proportion of flooded areas	0.01224
Urban-rural catchment areas (URCA)	0.00908
Proportion of habitat loss	0.00661
Current distribution of Indigenous and non-indigenous peoples	0.00171
r2	0.04

Pteroglossus

Current distribution of family languages	0.00387
Historical distribution of Indigenous family languages	0.00294
Annual Net Primary Productivity (NPP)	0.00135
Annual Gross Primary Productivity (GPP)	0.00107
Soil fertility	0.00065
Proportion of flooded areas	0.00064
Height above the nearest drainage (HAND)	0.00051
Enhanced Vegetation Index (EVI)	0.00048
Elevation	0.00036
Proportion of habitat loss	0.00029
Urban-rural catchment areas (URCA)	0.00010
Current distribution of Indigenous and non-indigenous peoples	0.00003
r2	0.218

Pteronura brasiliensis

Soil fertility	0.00017
Elevation	0.00007
Enhanced Vegetation Index (EVI)	0.00006
Annual Gross Primary Productivity (GPP)	0.00005
Proportion of flooded areas	0.00005
Annual Net Primary Productivity (NPP)	0.00005
Height above the nearest drainage (HAND)	0.00005
Historical distribution of Indigenous family languages	0.00004
Current distribution of family languages	0.00003
Proportion of habitat loss	0.00003
Current distribution of Indigenous and non-indigenous peoples	0.00001
Urban-rural catchment areas (URCA)	0.00000
r2	0.06

Puma concolor

Historical distribution of Indigenous family languages	0.00176
Proportion of habitat loss	0.00153
Elevation	0.00127
Proportion of flooded areas	0.00113
Height above the nearest drainage (HAND)	0.00111
Urban-rural catchment areas (URCA)	0.00110
Soil fertility	0.00105
Annual Net Primary Productivity (NPP)	0.00100
Enhanced Vegetation Index (EVI)	0.00095
Annual Gross Primary Productivity (GPP)	0.00090
Current distribution of family languages	0.00006
Current distribution of Indigenous and non-indigenous peoples	0.00002
r2	0.015

Rallidae

Annual Gross Primary Productivity (GPP)	0.00086
Annual Net Primary Productivity (NPP)	0.00078
Urban-rural catchment areas (URCA)	0.00070
Historical distribution of Indigenous family languages	0.00056
Proportion of flooded areas	0.00052
Height above the nearest drainage (HAND)	0.00050
Elevation	0.00045
Soil fertility	0.00043
Enhanced Vegetation Index (EVI)	0.00043
Proportion of habitat loss	0.00029
Current distribution of family languages	0.00027
Current distribution of Indigenous and non-indigenous peoples	0.00002
r2	-0.116

Ramphastos

Annual Net Primary Productivity (NPP)	0.09481
Historical distribution of Indigenous family languages	0.08263
Annual Gross Primary Productivity (GPP)	0.05373
Current distribution of family languages	0.04442
Elevation	0.04403
Soil fertility	0.03557
Height above the nearest drainage (HAND)	0.02979
Proportion of flooded areas	0.02692
Enhanced Vegetation Index (EVI)	0.02689
Urban-rural catchment areas (URCA)	0.01366
Proportion of habitat loss	0.01060
Current distribution of Indigenous and non-indigenous peoples	0.00243
r2	-0.017

Rhea americana

Annual Net Primary Productivity (NPP)	0
Proportion of flooded areas	0
Elevation	0
Historical distribution of Indigenous family languages	0
Current distribution of family languages	0
Annual Gross Primary Productivity (GPP)	0
Urban-rural catchment areas (URCA)	0
Current distribution of Indigenous and non-indigenous peoples	0
Enhanced Vegetation Index (EVI)	0
Height above the nearest drainage (HAND)	0
Soil fertility	0
Proportion of habitat loss	0
r2	-0.492

Rhinemys rufipes

Elevation	0.00089
Enhanced Vegetation Index (EVI)	0.00038
Historical distribution of Indigenous family languages	0.00037
Annual Net Primary Productivity (NPP)	0.00028
Soil fertility	0.00026
Annual Gross Primary Productivity (GPP)	0.00025
Current distribution of family languages	0.00019
Proportion of flooded areas	0.00019
Height above the nearest drainage (HAND)	0.00015
Proportion of habitat loss	0.00008
Urban-rural catchment areas (URCA)	0.00001
Current distribution of Indigenous and non-indigenous peoples	0.00000
r2	0.011

Rhinoclemmys punctularia

Current distribution of family languages	0.00003
Elevation	0.00001
Proportion of flooded areas	0.00001
Soil fertility	0.00001
Enhanced Vegetation Index (EVI)	0.00000
Annual Gross Primary Productivity (GPP)	0.00000
Height above the nearest drainage (HAND)	0.00000
Proportion of habitat loss	0.00000
Current distribution of Indigenous and non-indigenous peoples	0.00000
Annual Net Primary Productivity (NPP)	0.00000
Urban-rural catchment areas (URCA)	0.00000
Historical distribution of Indigenous family languages	0.00000
r2	-0.188

Saguinus

Annual Net Primary Productivity (NPP)	0.00025
Soil fertility	0.00019
Annual Gross Primary Productivity (GPP)	0.00017
Historical distribution of Indigenous family languages	0.00014
Proportion of flooded areas	0.00012
Elevation	0.00012
Enhanced Vegetation Index (EVI)	0.00011
Current distribution of family languages	0.00010
Height above the nearest drainage (HAND)	0.00007
Urban-rural catchment areas (URCA)	0.00007
Proportion of habitat loss	0.00004
Current distribution of Indigenous and non-indigenous peoples	0.00001
r2	0.029

Saimiri

Annual Net Primary Productivity (NPP)	0.01812
Historical distribution of Indigenous family languages	0.01369
Annual Gross Primary Productivity (GPP)	0.01325
Soil fertility	0.00690
Current distribution of family languages	0.00630
Elevation	0.00516
Enhanced Vegetation Index (EVI)	0.00511
Height above the nearest drainage (HAND)	0.00401
Proportion of flooded areas	0.00302
Proportion of habitat loss	0.00210
Current distribution of Indigenous and non-indigenous peoples	0.00141
Urban-rural catchment areas (URCA)	0.00105
r2	0.07

Sapajus

Current distribution of family languages	0.20849
Elevation	0.17280
Proportion of habitat loss	0.14786
Annual Gross Primary Productivity (GPP)	0.14479
Soil fertility	0.11740
Historical distribution of Indigenous family languages	0.11565
Enhanced Vegetation Index (EVI)	0.11324
Annual Net Primary Productivity (NPP)	0.11041
Height above the nearest drainage (HAND)	0.10159
Proportion of flooded areas	0.09676
Urban-rural catchment areas (URCA)	0.04646
Current distribution of Indigenous and non-indigenous peoples	0.02473
r2	0.175

Sarcoramphus papa

Current distribution of family languages	0.00038
Historical distribution of Indigenous family languages	0.00021
Soil fertility	0.00018
Height above the nearest drainage (HAND)	0.00014
Annual Gross Primary Productivity (GPP)	0.00012
Annual Net Primary Productivity (NPP)	0.00011
Enhanced Vegetation Index (EVI)	0.00008
Proportion of flooded areas	0.00007
Elevation	0.00007
Current distribution of Indigenous and non-indigenous peoples	0.00004
Proportion of habitat loss	0.00002
Urban-rural catchment areas (URCA)	0.00001
r2	-0.05

Sciurus (Hadrosciurus)

Historical distribution of Indigenous family languages	0.06780
Elevation	0.04270
Soil fertility	0.04242
Height above the nearest drainage (HAND)	0.03176
Enhanced Vegetation Index (EVI)	0.02560
Annual Net Primary Productivity (NPP)	0.02487
Proportion of flooded areas	0.02186
Annual Gross Primary Productivity (GPP)	0.02184
Current distribution of family languages	0.01652
Proportion of habitat loss	0.01507
Current distribution of Indigenous and non-indigenous peoples	0.00411
Urban-rural catchment areas (URCA)	0.00324
r2	0.293

Sciurus (Notosciurus)

Elevation	0.00003
Enhanced Vegetation Index (EVI)	0.00003
Soil fertility	0.00003
Annual Gross Primary Productivity (GPP)	0.00002
Proportion of flooded areas	0.00002
Annual Net Primary Productivity (NPP)	0.00002
Current distribution of family languages	0.00002
Height above the nearest drainage (HAND)	0.00002
Historical distribution of Indigenous family languages	0.00001
Proportion of habitat loss	0.00001
Urban-rural catchment areas (URCA)	0.00000
Current distribution of Indigenous and non-indigenous peoples	0.00000
r2	0.266

Scolopacidae

Annual Gross Primary Productivity (GPP)	0.00357
Urban-rural catchment areas (URCA)	0.00186
Height above the nearest drainage (HAND)	0.00079
Proportion of flooded areas	0.00074
Elevation	0.00066
Proportion of habitat loss	0.00065
Historical distribution of Indigenous family languages	0.00042
Annual Net Primary Productivity (NPP)	0.00033
Enhanced Vegetation Index (EVI)	0.00018
Soil fertility	0.00010
Current distribution of family languages	0.00002
Current distribution of Indigenous and non-indigenous peoples	0.00000
r2	-0.348

Selenidera

Current distribution of family languages	0.01770
Proportion of habitat loss	0.01353
Historical distribution of Indigenous family languages	0.01060
Urban-rural catchment areas (URCA)	0.01019
Elevation	0.01009
Soil fertility	0.00592
Height above the nearest drainage (HAND)	0.00492
Annual Net Primary Productivity (NPP)	0.00453
Enhanced Vegetation Index (EVI)	0.00337
Proportion of flooded areas	0.00291
Annual Gross Primary Productivity (GPP)	0.00152
Current distribution of Indigenous and non-indigenous peoples	0.00011
r2	-0.256

Serpentes (others)

Soil fertility	0.00052
Enhanced Vegetation Index (EVI)	0.00044
Proportion of habitat loss	0.00034
Current distribution of family languages	0.00032
Proportion of flooded areas	0.00029
Historical distribution of Indigenous family languages	0.00023
Urban-rural catchment areas (URCA)	0.00021
Height above the nearest drainage (HAND)	0.00016
Annual Net Primary Productivity (NPP)	0.00015
Elevation	0.00012
Annual Gross Primary Productivity (GPP)	0.00004
Current distribution of Indigenous and non-indigenous peoples	0.00001
r2	-0.011

Sotalia fluviatilis

Soil fertility	0
Elevation	0
Annual Net Primary Productivity (NPP)	0
Enhanced Vegetation Index (EVI)	0
Annual Gross Primary Productivity (GPP)	0
Height above the nearest drainage (HAND)	0
Proportion of flooded areas	0
Historical distribution of Indigenous family languages	0
Proportion of habitat loss	0
Urban-rural catchment areas (URCA)	0
Current distribution of family languages	0
Current distribution of Indigenous and non-indigenous peoples	0
r2	-0.007

Spatula discors

Annual Net Primary Productivity (NPP)	0
Annual Gross Primary Productivity (GPP)	0
Enhanced Vegetation Index (EVI)	0
Soil fertility	0
Elevation	0
Height above the nearest drainage (HAND)	0
Proportion of flooded areas	0
Proportion of habitat loss	0
Urban-rural catchment areas (URCA)	0
Historical distribution of Indigenous family languages	0
Current distribution of Indigenous and non-indigenous peoples	0
Current distribution of family languages	0
r2	NaN

Speothos venaticus

Height above the nearest drainage (HAND)	0.00004
Current distribution of family languages	0.00003
Annual Net Primary Productivity (NPP)	0.00003
Elevation	0.00003
Proportion of habitat loss	0.00002
Annual Gross Primary Productivity (GPP)	0.00002
Proportion of flooded areas	0.00001
Current distribution of Indigenous and non-indigenous peoples	0.00001
Soil fertility	0.00001
Enhanced Vegetation Index (EVI)	0.00001
Historical distribution of Indigenous family languages	0.00000
Urban-rural catchment areas (URCA)	0.00000
r2	-0.148

Strigidae

Historical distribution of Indigenous family languages	0.00003
Current distribution of family languages	0.00002
Enhanced Vegetation Index (EVI)	0.00002
Annual Net Primary Productivity (NPP)	0.00002
Annual Gross Primary Productivity (GPP)	0.00002
Soil fertility	0.00002
Elevation	0.00001
Urban-rural catchment areas (URCA)	0.00001
Proportion of flooded areas	0.00000
Height above the nearest drainage (HAND)	0.00000
Proportion of habitat loss	0.00000
Current distribution of Indigenous and non-indigenous peoples	0.00000
r2	-0.272

Sylvilagus

Height above the nearest drainage (HAND)	0.00496
Enhanced Vegetation Index (EVI)	0.00434
Historical distribution of Indigenous family languages	0.00184
Annual Net Primary Productivity (NPP)	0.00170
Elevation	0.00169
Proportion of habitat loss	0.00167
Annual Gross Primary Productivity (GPP)	0.00153
Urban-rural catchment areas (URCA)	0.00108
Soil fertility	0.00106
Proportion of flooded areas	0.00073
Current distribution of family languages	0.00035
Current distribution of Indigenous and non-indigenous peoples	0.00012
r2	-0.154

Tamandua tetradactyla

Soil fertility	0.00948
Historical distribution of Indigenous family languages	0.00827
Annual Net Primary Productivity (NPP)	0.00598
Current distribution of family languages	0.00561
Enhanced Vegetation Index (EVI)	0.00388
Annual Gross Primary Productivity (GPP)	0.00368
Height above the nearest drainage (HAND)	0.00345
Elevation	0.00306
Proportion of flooded areas	0.00248
Urban-rural catchment areas (URCA)	0.00074
Proportion of habitat loss	0.00049
Current distribution of Indigenous and non-indigenous peoples	0.00032
r2	0.008

Tapirus terrestris

Annual Net Primary Productivity (NPP)	0.06801
Height above the nearest drainage (HAND)	0.06317
Current distribution of family languages	0.06206
Annual Gross Primary Productivity (GPP)	0.05863
Historical distribution of Indigenous family languages	0.04921
Soil fertility	0.04686
Enhanced Vegetation Index (EVI)	0.04655
Elevation	0.04538
Proportion of flooded areas	0.03962
Proportion of habitat loss	0.02377
Urban-rural catchment areas (URCA)	0.01097
Current distribution of Indigenous and non-indigenous peoples	0.00464
r2	-0.023

Tayassu pecari

Historical distribution of Indigenous family languages	1.86592
Annual Net Primary Productivity (NPP)	1.33171
Enhanced Vegetation Index (EVI)	1.30490
Annual Gross Primary Productivity (GPP)	1.29270
Current distribution of family languages	1.18595
Height above the nearest drainage (HAND)	1.17439
Proportion of flooded areas	1.16498
Soil fertility	1.12807
Elevation	1.02232
Proportion of habitat loss	0.61479
Urban-rural catchment areas (URCA)	0.47948
Current distribution of Indigenous and non-indigenous peoples	0.13137
r2	0.244

Theristicus caudatus

Current distribution of family languages	0.00027
Historical distribution of Indigenous family languages	0.00017
Height above the nearest drainage (HAND)	0.00008
Elevation	0.00005
Proportion of flooded areas	0.00003
Enhanced Vegetation Index (EVI)	0.00002
Current distribution of Indigenous and non-indigenous peoples	0.00001
Annual Net Primary Productivity (NPP)	0.00001
Soil fertility	0.00001
Annual Gross Primary Productivity (GPP)	0.00000
Urban-rural catchment areas (URCA)	0.00000
Proportion of habitat loss	0.00000
r2	-0.068

Threskiornithidae (others)

Annual Net Primary Productivity (NPP)	0
Annual Gross Primary Productivity (GPP)	0
Enhanced Vegetation Index (EVI)	0
Elevation	0
Urban-rural catchment areas (URCA)	0
Soil fertility	0
Height above the nearest drainage (HAND)	0
Proportion of habitat loss	0
Proportion of flooded areas	0
Historical distribution of Indigenous family languages	0
Current distribution of Indigenous and non-indigenous peoples	0
Current distribution of family languages	0
r2	-0.076

Tinamus

Proportion of habitat loss	0.15216
Elevation	0.06929
Historical distribution of Indigenous family languages	0.06566
Annual Gross Primary Productivity (GPP)	0.04883
Urban-rural catchment areas (URCA)	0.04854
Current distribution of family languages	0.04807
Proportion of flooded areas	0.04561
Annual Net Primary Productivity (NPP)	0.04547
Height above the nearest drainage (HAND)	0.03438
Soil fertility	0.03280
Enhanced Vegetation Index (EVI)	0.02539
Current distribution of Indigenous and non-indigenous peoples	0.00321
r2	-0.139

Tolypeutes tricinctus

Height above the nearest drainage (HAND)	0.00024
Proportion of habitat loss	0.00011
Elevation	0.00004
Soil fertility	0.00003
Urban-rural catchment areas (URCA)	0.00003
Historical distribution of Indigenous family languages	0.00002
Annual Net Primary Productivity (NPP)	0.00001
Annual Gross Primary Productivity (GPP)	0.00001
Proportion of flooded areas	0.00001
Enhanced Vegetation Index (EVI)	0.00001
Current distribution of Indigenous and non-indigenous peoples	0.00000
Current distribution of family languages	0.00000
r2	-0.191

Tremarctos ornatus

Height above the nearest drainage (HAND)	0
Urban-rural catchment areas (URCA)	0
Annual Gross Primary Productivity (GPP)	0
Current distribution of Indigenous and non-indigenous peoples	0
Elevation	0
Historical distribution of Indigenous family languages	0
Current distribution of family languages	0
Annual Net Primary Productivity (NPP)	0
Soil fertility	0
Enhanced Vegetation Index (EVI)	0
Proportion of habitat loss	0
Proportion of flooded areas	0
r2	-0.298

Trichechus inunguis

Annual Net Primary Productivity (NPP)	0.00115
Annual Gross Primary Productivity (GPP)	0.00112
Soil fertility	0.00099
Elevation	0.00087
Enhanced Vegetation Index (EVI)	0.00082
Proportion of flooded areas	0.00066
Historical distribution of Indigenous family languages	0.00050
Height above the nearest drainage (HAND)	0.00048
Current distribution of family languages	0.00020
Proportion of habitat loss	0.00016
Current distribution of Indigenous and non-indigenous peoples	0.00006
Urban-rural catchment areas (URCA)	0.00003
r2	-0.033

Trochilidae

Historical distribution of Indigenous family languages	0.00009
Soil fertility	0.00001
Annual Gross Primary Productivity (GPP)	0.00001
Elevation	0.00001
Annual Net Primary Productivity (NPP)	0.00001
Enhanced Vegetation Index (EVI)	0.00000
Height above the nearest drainage (HAND)	0.00000
Proportion of flooded areas	0.00000
Current distribution of family languages	0.00000
Urban-rural catchment areas (URCA)	0.00000
Current distribution of Indigenous and non-indigenous peoples	0.00000
Proportion of habitat loss	0.00000
r2	-0.029

Trogonidae

Current distribution of family languages	0.00010
Historical distribution of Indigenous family languages	0.00008
Height above the nearest drainage (HAND)	0.00002
Annual Net Primary Productivity (NPP)	0.00001
Elevation	0.00001
Annual Gross Primary Productivity (GPP)	0.00001
Soil fertility	0.00001
Enhanced Vegetation Index (EVI)	0.00000
Urban-rural catchment areas (URCA)	0.00000
Current distribution of Indigenous and non-indigenous peoples	0.00000
Proportion of habitat loss	0.00000
Proportion of flooded areas	0.00000
r2	-0.039

Tupinambis teguixin

Enhanced Vegetation Index (EVI)	0.00799
Current distribution of family languages	0.00505
Annual Gross Primary Productivity (GPP)	0.00430
Annual Net Primary Productivity (NPP)	0.00228
Soil fertility	0.00135
Historical distribution of Indigenous family languages	0.00093
Height above the nearest drainage (HAND)	0.00086
Elevation	0.00084
Proportion of flooded areas	0.00073
Current distribution of Indigenous and non-indigenous peoples	0.00061
Proportion of habitat loss	0.00059
Urban-rural catchment areas (URCA)	0.00015
r2	-0.094

undetermined

Annual Net Primary Productivity (NPP)	0.20131
Elevation	0.12959
Height above the nearest drainage (HAND)	0.12410
Annual Gross Primary Productivity (GPP)	0.11901
Soil fertility	0.07990
Historical distribution of Indigenous family languages	0.07937
Current distribution of family languages	0.07903
Proportion of flooded areas	0.06811
Enhanced Vegetation Index (EVI)	0.06390
Proportion of habitat loss	0.06381
Urban-rural catchment areas (URCA)	0.02901
Current distribution of Indigenous and non-indigenous peoples	0.00288
r2	-0.045

Supplementary Table 8. Estimated proportion of the number of animals hunted (Individual Animals Offtake) for the 30 most hunted in relation to the total offtake in areas with < 70% of habitat loss and areas with > 70% of habitat loss.

Areas with < 70% of habitat loss			Areas with > 70% of habitat loss	
Taxon	Individuals Offtake proportion		Taxon	Individuals Offtake proportion
Cuniculus paca	0.127		Cuniculus paca	0.134
Tayassu pecari	0.101		Tayassu pecari	0.088
Dasyprocta	0.075		Dicotyles tajacu	0.071
Dicotyles tajacu	0.068		Dasyprocta	0.065
undetermined	0.043		Dasypus novemcinctus	0.062
Chelonoidis	0.039		Penelope	0.055
Dasypus novemcinctus	0.037		Chelonoidis	0.041
Penelope	0.035		Tinamus	0.039
Mazama americana	0.031		undetermined	0.037
Mitu	0.023		Hydrochoerus hydrochaeris	0.032
Alouatta	0.022		Mazama americana	0.030
Podocnemis unifilis	0.021		Sapajus	0.028
Mazama nemorivaga	0.019		Mitu	0.026
Tinamus	0.018		Podocnemis unifilis	0.023
Sapajus	0.017		Mazama nemorivaga	0.020
Hydrochoerus hydrochaeris	0.016		Alouatta	0.012
Cairina moschata	0.015		Nasua nasua	0.011
Ateles	0.014		Tapirus terrestris	0.011
Tapirus terrestris	0.014		Crax	0.008
Ramphastos	0.012		Crypturellus	0.008

Nasua nasua	0.011		Columbidae (others)	0.008
Lagothrix	0.010		Cairina moschata	0.008
Nannopterum brasilianus	0.010		Caiman crocodilus	0.007
Caiman crocodilus	0.008		Dasypus septemcinctus	0.007
Crypturellus	0.008		Amazona	0.007
Psophia	0.008		Euphractus sexcinctus	0.006
Patagioenas	0.007		Patagioenas	0.006
Ardeidae (others)	0.007		Psophia	0.006
Podocnemis expansa	0.007		Iguana iguana	0.006
Podocnemis sextuberculata	0.007		Anura	0.006
Amazona	0.007		Ramphastos	0.006

Supplementary Discussion 1. The role of traditional wild meat food systems in advancing the Sustainable Development Goals (SDGs) in Amazonia.

SDG 1 – No Poverty

Wild meat plays a vital role in traditional Amazonian food systems, supporting nutrition and livelihoods, while sustainable wildlife management reinforces the territorial rights and biodiversity stewardship of Amazonian Peoples. With an estimated hidden economic value exceeding US$2 billion annually, wild meat contributes significantly to the economies and livelihoods of the region. As a critical ecosystem service, it offers affordable, nutrient-rich food and reduces household food expenditures in areas where cash income is scarce. Once deprived of their access to wild meat, Amazonian Peoples depend on costly market-based or domestic alternatives, exacerbating poverty and undermining food sovereignty.

SDG 2 – Zero Hunger

Wild meat is critical in ensuring food security for nearly 11 million rural inhabitants in Amazonia by providing essential calories and high-quality protein. It meets a substantial portion of daily nutritional needs, covering up to half of the recommended protein and iron intake, helping combat malnutrition and improving health outcomes, particularly among children, pregnant women, and lactating mothers. Rooted in forest-based traditions, sustainable hunting practices not only preserve biodiversity but also ensure the long-term viability of this vital food source. This is especially crucial in remote areas where access to market-based foods is limited or prohibitively expensive.

SDG 3 – Good Health and Well-Being

Wild meat plays a critical role in maintaining the dietary health of Amazonian Peoples, particularly through its high levels of essential micronutrients such as vitamin B12, iron, and zinc, deficiencies of which are associated with severe health conditions. Access to wild meat is positively linked to improved health outcomes in children, including higher haemoglobin levels and better growth indicators. There are lower malnutrition rates and anaemia among Amazonian Peoples, whose diets traditionally rely on wild meat. When access to wild meat is restricted, rural peoples are often forced to substitute it with less nutritious, frequently more expensive, alternatives, leading to poorer health and increased vulnerability to food insecurity.

SDG 4 – Quality Education

Preserving traditional food systems, the Amazonian system of knowledge, and sociocultural practices, including traditional hunting passed intergenerationally, supports the culturally respectful education of Amazonian Peoples.

SDG 5 – Gender Equality

Women play a central role in wild meat preparation, distribution, and governance, helping to maintain the community's social ties and cultural transmission. Wild meat also supports the nutritional needs of pregnant and lactating women.

SDG 8 – Decent Work and Economic Growth

The hidden multibillion-dollar value of traditional hunting and wild meat production contributes to the resilience and economic stability of Amazonian Peoples by supporting livelihoods and preventing similar costs in domestic meat purchases in a region where formal economic opportunities are limited.

SDG 10 – Reduced Inequalities

Recognising hunting and wild meat access rights reduces marginalisation and empowers the autonomy and food sovereignty of Amazonian Peoples.

SDG 12 – Responsible Consumption and Production

When grounded in Amazonian systems of knowledge and sociocultural practices, traditional hunting can contribute to forest conservation and support the sustainable use of wildlife, provided that local norms, taboos, and ecological feedbacks remain intact and are not disrupted by external pressures.

SDG 13– Climate Action

Traditional hunting and wild meat food systems rely on healthy forests and provide a low-carbon alternative to industrial livestock production, contributing to climate change mitigation. Replacing wild meat with beef would necessitate large-scale deforestation and could increase CO_2 emissions by as much as 12% of global annual emissions.

SDG 14 – Life Below Water

Traditional hunting and wild meat food systems depend on the interplay and conservation of terrestrial and aquatic systems. Aquatic and semi-aquatic wildlife are vital to wild meat food systems in Amazonia. Protected territories encompassing rivers, lakes, and floodplains play a crucial role in conserving freshwater biodiversity, sustaining healthy fish and other wildlife populations, and deterring illegal fishing through robust local governance and time-honoured stewardship practices.

SDG 15 – Life on Land

Preserving Amazon forests is essential for sustaining biodiversity and viable populations of hunted species. Safeguarding Indigenous and Traditional territories is vital to maintaining Amazonia's traditional wild meat food systems. Wild meat is a critical food source and a social cornerstone that motivates Amazonian Peoples to safeguard their territories. The health of Amazonian ecosystems and wildlife is inextricably linked to the well-being of Amazonian Peoples, underscoring the importance of recognising their land rights and supporting policies that enhance their autonomy and governance over their territories and biodiversity.

SDG 16 – Peace, Justice, and Strong Institutions

Recognizing traditional hunting and wild meat access rights reduces marginalization and empowers Amazonian Peoples. Supporting territory rights and community-led wildlife management strengthens their food sovereignty, self-determination, autonomy and governance.

SDG 17 – Partnerships for the Goals

Collaborative wildlife management and research are vital to enhance the preservation of Amazonian ecosystems and their rich cultural and biological diversity, ultimately contributing to achieving the Sustainable Development Goals (SDGs).

Supplementary Method 4. Overview of primary data collection methods and related ethical procedures.

Country	Person / Institution responsible	Number of communities involved	Period of data collection	Marupiara internal agreement signed	Data collection purpose			Ethical approval				Methodology description
					Community-based wildlife management initiative	Collaborative research initiative	Governmental wildlife monitoring program	Oral agreement with the communities involved	Written agreement with the communities involved	Official government approval	Ethics committee approval	
Brazil	Boubli J. University of Salford, Salford, UK	1	1994	X		X		X		Licença FUNAI (CGEP 93/065); Licença FUNAI (CGEP 94/112)		Boubli et al. (2020)
Brazil	Constantino PAL. Rede de Pesquisa em Conservação, Uso e Manejo da Fauna da Amazônia (RedeFauna), Manaus, Brasil	67	1999-2011	X	X	X		X	X	Licença FUNAI (No 2952/08)	Protocol #2009-U-489 / University of Florida	Constantino et al. (2008), Constantino et al. (2012a), Constantino et al. (2012b), Constantino (2020)
Brazil	Instituto Desenvolvimento Sustentável Mamirauá (IDSM), Tefé, Brazil	11	1999-2019	X	X		X	X		Licença SISBIO (No 29092-3)	CEUAP 001/2011 / Instituto Mamirauá	Constantino et al. (2012a), Morcatty & Valsecchi (2015); Pinho et al. (2022)
Brazil	Secretaria de Meio Ambiente e Desenvolvimento Sustentável do Estado do Amazonas (ProBUC program), Manaus, Brazil.	39	2007-2013	X			X	X		Lei Complementar Nº 53/2007 de 05/06/2007; Decreto No 8.505, de 20 de agosto de 2015		Constantino et al. (2012a); Costa et al. (2018, 2019), Bucheli & Marinelli (2014); Costa & Marchand GA (2014)
Brazil	Pezzuti J., Universidade Federal do Pará, Belém, Brazil.	20	2007-2022	X	X	X		X		Licença SISBIO (No 17323-3); Licença SISBIO (50927-5); Licença ICMBio (Ofício No 114/2013-RRX-NGI ATM/CR3/ICMBIO)		de Paula et al. (2022); Ribeiro et al. (2019); Ponce-Martins et al. (2022)
Brazil	Instituto Piagaçu, Manaus, Brazil.	11	2011-2014	X	X		X	X		Autorização CEUC/SDS-AM (No 052/2011)	CEP Processo nº 017-11 / Instituto Nacional de Pesquisas da Amazônia	Mattos Vieira et al. (2015)
Brazil	Instituto Chico Mendes de Conservação da Biodiversidade (ICMBio). Monitora program.	83	2014-2019	X			X	X		Instrução Normativa No 3/2017/GABIN/ICMBio; Instrução Normativa No 2/2022/GABIN/ICMBio		
Brazil	Antunes AP. Instituto Nacional de Pesquisas da Amazônia (INPA), Rede de Pesquisa em Conservação, Uso e Manejo da Fauna da Amazônia (RedeFauna), Manaus, Brasil	19	2015-2024	X	X	X		X	Ofício N° 366/2023-FOIRN.	Licença Funai (No 08620.002266/2019-10); Licença Funai (No 171/2023/CR-RNG/FUNAI)		Antunes et al. (2015); Antunes (2023, 2024)
Brazil	Valle AN. Universidade Federal de Mato Grosso do Sul, Brazil.	1	2016	X		X		X			CAAE: 60121316.8.0000.0021 / CONEP - Universidade	Nunes et al. (2020)

											Federal de Mato Grosso do Sul	
Peru	Mayor P, Bodmer RE. FundAmazonia, Museum of Amazonian Indigenous Cultures, Iquitos, Peru.	40	1991-2020	X	X	X		X		Carta No 008-2006-INRENA-J-IFFS; N" 03 - 2012 - SERNANP- RN Pucaruro-JEF; Resolución Directoral No 0127-2010-AG-DGFFS-DGEFFS; Resolución Directoral No 0229-2011-AG-DGFFS-DGEFFS; Resolución Directoral No 0350-2012-AG-DGFFS-DGEFFS; Resolución Directoral No 0249-2013-MINAGRI-AG-DGFFS-DGEFFS; Resolución de Dirección General No 258-2019-MINAGRI-SERFOR-DGGSPFFS; Autorización No 041-2007-INRENA-IFFS-DCB; Autorización No 108-2008-INRENA-IFFS-DCB.	CONSTANCIA 270-10-19; CONSTANCIA 029-03-19 / Universidade Peruana Cayetano Heredia	Puertas & Bodmer (2004)
French Guyana	Office Français de la Biodiversité (OFB) y Parc Amazonien de Guyane (PAG)	36	1999-2018	X			X	X		Decree No. 2007-266; Law No. 2019-773.		Richard-Hansen et al. (2019)

References of scientific publications with descriptions of methodologies applied in these wildlife management, research and monitoring initiatives

Antunes AP, Muhlen EMV, Rossoni FC, Ventincinque EM. Monitoramento de fauna Paumari nas Terras Indígenas Paumari. (Operação Amazônia Nativa, Cuiabá, 2015).

Antunes AP. Manejo Territorial de Fauna em Terras Indígenas da Amazônia. Relatório Final. (PCI/INPA/MCTI, 2023)

Antunes AP. Wildlife participatory management in two indigenous territories of Amazonia. Final Report. (National Geographic Society, 2024).

Boubli, J. P., Urbani, B., Caballero-Arias, H., Shepard, G. H., Lizarralde, M. Primates in the lives of the Yanomami people of Brazil and Venezuela in Neotropical Ethnoprimatology (eds Urbani, B. & Lizarralde, M.) 199–224 (Springer, Cham., 2020). doi.org/10.1007/978-3-030-27504-4_9

Bucheli MGA and Marinelli CE, 2014. Custo-eficiência de Programas de Monitoramento Participativo da Biodiversidade: o Caso do Probuc (Programa de Monitoramento da Biodiversidade e do Uso de Recursos Naturais em Unidades de Conservação Estaduais do Amazonas). Biodiversidade Brasileira 4(1): 47-68

Constantino, P. A. L. et al. Indigenous collaborative research for wildlife management in Amazonia: the case ofKaxinawá, Acre, Brazil. Biological Conservation 141, 2718-2729. (2008). doi.org/10.1016/j.biocon.2008.08.008

Constantino, P. A. L. et al. Empowering local people through community-based resource monitoring: a comparison of Brazil and Namibia. Ecology and Society 17(4): 22. (2012a). doi.org/10.5751/ES-05164-170422

Constantino, P. A. L. et al. Monitoramento e mapeamento participativo da caça na Terra Indígena Kaxinawá da Praia do Carapanã (Acre) in Conservação da Biodiversidade com SIG (eds Paese, A., Uezu, A., Lorini, M. L. & Cunha, A.) 141-53 (Oficina de Textos, São Paulo, 2012b).

Constantino, P. A. L. Challenges of forest citizen involvement in biodiversity monitoring in protected areas of Brazilian Amazonia in Handbook of Citizen Science in Ecology and Conservation (eds Lepczyk C. A., Bolye O. D. & Vargo T. L. V.) 237-295 (University of California Press, Berkeley, 2020). doi.org/10.2307/j.ctvz0h8fz.23.

Costa DC, Marchand GAEL, Pereira HS, 2019. Monitoramento participativo da biodiversidade em sistemas de unidades de conservação: o caso do ProBUC no estado do Amazonas. Amazônica - Revista de Antropologia, 11: 219-232

Costa DC, Pereira HS, Marchand GAEL, Silva CP 2018. Challenges of Participatory Community Monitoring of Biodiversity in Protected Areas in Brazilian Amazon. Diversity, 10: 61.

Costa DC and Marchand GA, 2014. O programa de monitoramento da biodiversidade e do uso sustentável de recursos naturais –ProBUC – como alternativa de monitoramento comunitário amazônico. Revista Monografias Ambientais 13(3): 3383-3391

Fagundes, I. S. F. Sistema de monitoramento de uso dos recursos naturais (SiMUR) na Reserva Extrativista Rio Unini - Barcelos/AM (2008-2020). PhD thesis (Universidade Federal do Amazonas, Manaus, 2021)

de Mattos Vieira, M. A. R., von Muhlen, E. M. & Shepard Jr, G. H. Participatory monitoring and management of subsistence hunting in the Piagaçu-Purus reserve, Brazil. Conservation and Society 13, 254-264.(2015). doi.org/10.4103/0972-4923.170399

Morcatty, T. Q. & Valsecchi, J. Social, biological, and environmental drivers of the hunting and trade of the endangered yellow-footed tortoise in the Amazon. Ecology and Society 20(3), 3. (2015). doi.org/10.5751/ES-07701-200303

Nunes, A. V., Oliveira-Santos, L. G. R., Santos, B. A. et al. Socioeconomic Drivers of Hunting Efficiency and Use of Space By Traditional Amazonians. Human Ecology 48, 307–315. (2020). doi.org/10.1007/s10745-020-00152-6

Oliveira, M. A. et al. The role of religion, wealth, and livelihoods in the hunting practices of urban and rural Inhabitants in Western Amazonia. Human Ecology 51, 1239–1252. (2023). doi.org/10.1007/s10745-023-00467-0

de Paula, M. J., Carvalho, E. A., Lopes, C. K. M., et al. Hunting sustainability within two eastern Amazon Extractive Reserves. Environmental Conservation. 49(2), 90-98. (2022). doi:10.1017/S0376892922000145

de Pinho, F. F. et al. Modelling the impact of hunting on the coexistence of congeneric deer species in Central Amazonia. Journal of Zoology 317, 195-204. (2022). doi.org/10.1111/jzo.12970

Ponce-Martins, M., Lopes, C.K.M., de Carvalho-Jr, E.A.R., dos Reis Castro, F.M., de Paula, M.J. and Pezzuti, J.C.B. Assessing the contribution of local experts in monitoring Neotropical vertebrates with camera traps, linear transects and track and sign surveys in the Amazon. Perspectives in Ecology and Conservation, 20(4), pp.303-313 (2022).

Puertas, P. & Bodmer, R. Hunting effort as a tool for community-based wildlife management in Amazonia in People in Nature: Wildlife Conservation in South and Central America (eds Silvius, K., Bodmer, R. & Fragoso J.) 123-138 (Columbia University Press, New York, 2004). doi.org/10.7312/silv12782-008

Ribeiro KT et al. 2021. Programa Monitora: Histórico de criação e sua relação com a agenda socioambiental. In: Encontro de saberes: uma nova forma de conversar a conservação. IPE.

Richard-Hansen, C. et al. Hunting in French Guiana across time, space and livelihoods. Frontiers in Ecology and Evolution 7, 289. (2019). doi.org/10.3389/fevo.2019.00289

Santos Junior, M. An. et al. SiMUR: Uma Experiência Participativa do Monitoramento do Uso de Recursos Naturais. Technical Report (Fundação Vitória Amazônica, Manaus, 2018). https://www.gov.br/icmbio/pt-br/assuntos/monitoramento/conteudo/Materiais-de-Apoio/Bernanrdesetal2023RoteiroMetodologicoQuelonios.pdf

Supplementary Method 5. List of literature containing hunting data in rural areas of Amazonia.

1. Abrahams MI, Peres CA, Costa HCM. 2017. Measuring local depletion of terrestrial game vertebrates by central-place hunters in rural Amazonia. Plos One 12, e0186653. https://doi.org/10.1371/journal.pone.0186653

2. Aguirre LF, Urioste R, Galarza I, Miranda JC, Guayao E, Vaca D. 1999. El monitoreo de aprovechamiento de fauna em la Estación Biológica del Beni: Un análisis crítico. In, Manejo y conservación de la fauna silvestre en América Latina (Eds. Fang TG, Montenegro OL, Bodmer RE). Instituto de Ecología, La Paz, Bolivia. Pp 97-108.

3. Almeida M. 1992. Reservas extrativistas como estratégia de conservação de fauna. In Manejo da Vida Silvestre para a Conservação na América Latina (Bodmer R.) Relatório Técnico.

4. Alonzo JA. 1995. Estado actual de la fauna silvestre em la propuesta de la Reserva Comunal Pucacuro. In, Manejo y conservación de la fauna silvestre en América Latina (Eds. Fang TG, Bodmer RE, Aquino R, Valqui MH). Pp: 93-103. Iquitos.

5. Alvard, M. 1993. Testing the "Ecologically noble savage" hypothesis: interespecific prey choice by Piro hunters of Amazonian Peru. Human Ecology 21, 355-387.

6. Andrade PCM, Oliveira PHG, Oliveira AB, Almeida Júnior CD, Rodrigues WS. 2008. Levantamento da fauna com potencial cinegético da resex baixo Juruá, análise de sustentabilidade e propostas de manejo comunitário. Anais do Congresso Internacional sobre Manejo de Fauna Silvestre na Amazônia e América Latina.

7. Anonimous. Undated. Expedición al río Yavarí del 30 de octubre al 08 de diciembre, 2003.

8. Aquino R, Bodmer RE, Navarro JGG. 2001. Mamiferos de la cuenca del rio Samiria: ecologia poblacional y sustentabilidad de la caza. Wildlife Conservation Society. Programa Integral de Desarrollo y Conservación Pacaya Samiria.

9. Aquino R, Calle A. 2003. Evaluación del estado de conservación de los mamíferos de caza: um modelo comparativo en comunidades de la Reserva Nacional Pacaya Samiria (Loreto, Peru). Revista Peruana de Biologia 10, 163-174.

10. Aquino R, Terrones C, Navarro R, Terrones W. 2007 Evaluación del impacto de la caza en mamíferos de la cuenca delrío Alto Itaya, Amazonía peruana. Revista Peruana de Biologia 14, 181-186.

11. Aquino TV, Iglesias MP. 1992. Kaxinawa do Rio Jordão: Historia, território, economia e desenvolvimento sustentado. Comissão Pró-Índio do Acre. Rio Branco, AC.

12. Araújo, SSS. 2019.Da floresta nacional do Tapajós à exclusão: um estudo de caso da comunidade São Jorge, Belterra - Pará. MSc Dissertation, Universidade Federal do Oeste do Pará, Santarém, Brazil.

13. Arco APOD. 2013. Cacería de subsistencia y etnoecología asociada en una Comunidad Nativa Amahuaca de la Provincia de Purús- Ucayali, Perú. Thesis. Universidad Nacional Agraria La Molina. Lima, Peru.

14. Arhem K. 1976. Fishing and hunting among the Makuna: Economy, ideology and ecological adaptation in the northwes Amazon. Goteborgs Etnogrfiska Museum, Annals. Goteborg. Pp 27-44.

15. Ascorra C. 1999. Cazadores amazónicos y conservación de la fauna silvestre. In Perú: el problema agrario en debate (Eds. Agreda V, Diez A, Glave M). Seminario Permanente de Investigación Agraria. Pp 253-550.

16. Ayres JM, Ayres C. 1979. Aspectos da caça no alto rio Aripuanã. Acta Amazonica 9, 287-298. https://doi.org/10.1590/1809-43921979092287

17. Ayres JM, Lima MD, Martins ES, Barreiros JL. 1991. On the track of the road: changes in subsistence hunting in a Brazilian Amazonian villages. In Netropical wildlife use and conservation (Eds. Robinson JG, Redford KH). University of Chicago Press. Pp 53-92.

18. Balée W. 1985. Ka'apor ritual hunting. Human Ecology 13, 485-510. https://doi.org/10.1007/BF01531156

19. Bardales-Alvites C, Torres-Oyarce L, Tirado-Herrera E, Antúnes-Correa M. 2017. Presión de caza em mamiferos de la reserva nacional matsés, al noreste de la amazonia peruana. Folia Amazonica 26, 75-84.

20. Beckerman S. 1980. Fishing and hunting by the Bari of Colombia. In: Working Papers On South American Indians - Volume 2 (Ed Hames R). Bennington. Pp 67-109.

21. Bellido NLMZ. 2020. Caracterización de caza de subsistencia de mamíferos en la comunidad nativa nueva esperanza, cuenca del río Yavarí-Mirí, Loreto-Perú. Universidad Nacional Agraria La Molina.

22. Bodmer R, Aquino R, Navarro JGG. 2000. Sustentabilidad de la caza de mamíferos en la cuenca del rio Samiria, Amazonia Peruana. In Manejo de Fauna Silvestre en Amazonía y Latinoamérica (Eds. Cabrera E, Mercolli C, Resquín R.). CITES. Assuncion.

23. Bodmer R, Fang T, Ibañez LM. 1988. Ungulate management and conservation in the Peruvian Amazon. Biological Conservation 45, 303-310. https://doi.org/10.1016/0006-3207(88)90061-4

24. Bodmer RE, Fang TG, Moya L, Gill R. 1994. Managing wildlife to conserve Amazonian forests: population biology and economic considerations of game hunting. Biological conservation, 67(1), 29-35. https://doi.org/10.1016/0006-3207(94)90005-1

25. Bodmer RE, Pezo E. 1999. Análisis econômico del uso de fauna terrestre en la Amazonía Peruana. Manejo y Conservación de Fauna Silvestre en América Latina. Santa Cruz, Bolivia, 171-182.

26. Bodmer RE, Puertas P, Pérez P, Ríos C, Escobedo A, Santos A, Recharte M, Flores W, Arévalo F, Ruck L, Antúnez M, Valverde Z, Moya Jr. L, Freitas G. 2006. Estado actual de las especies paisajísticas de fauna silvestre y del monitoreo a grupos de manejo de la cuenca del Samiria. Technical Report. WCS.

27. Bonaudo T, Le Pendu Y, Faure JF, Quanz D. 2005. The effects of deforestation on wildlife along the transamazon highway. European Journal of Wildlife Research 51, 199-206. https://doi.org/10.1007/s10344-005-0092-1

28. Bonaudo T. Le Pendu Y, Chardonnet P, Jori F. 2001. Chasse de subsistance sur un front pionnier amazonien : le cas d’Uruará. Revue Élev. Méd. vét. Pays trop. 54, 281-286

29. Cabao MA, Nogales J, Justiniano F, Martinez A, Paz J, Vargas P, Townsend WR. 2008. El uso de la fauna silvestre durante un año en un asentamiento nuevo en la tco del pueblo indígena baure, beni – bolivia. Anais do Congresso Internacional sobre Manejo de Fauna Silvestre na Amazônia e América Latina

30. Cajaiba, RL, Silva, WB, Piovesan, PRR. 2015. Animais silvestres utilizados como recurso alimentar em assentamentos rurais no município de Uruará, Pará, Brasil. Desenvolvimento e Meio Ambiente 34, 157-168. https://doi.org/10.5380/dma.v34i0.38889

31. Calouro AM. 1995. Caça de subsistência: sustentabilidade e padrões de uso entre seringueiros ribeirinhos e não ribeirinhos do estado do acre. Thesis. Universidade de Brasília.

32. Campos MAA. 2008. Cruzando ecologias com os caçadores do Rio Cuieiras: saberes e estratégias de caça no Baixo Rio Negro, Amazonas. Thesis. Instituto Nacional de Pesquisas da Amazônia.

33. Campos R. 1977.Producción de pesca y caza en una comunidad Shipibo en el Río Pisqui. Amazonia Peruana 2, 53-74. https://doi.org/10.52980/revistaamazonaperuana.vi2.246

34. Carneiro DB. 2015. Como eu vivo, me sustento: Formas indígenas de usos de recursos naturais. Tese de Mestrado. UFOPA.

35. Castellanos HG, Bertsch C, Veit A, Valeris C, Sarmiento W, Rodrĩguez F. 2010. Cosecha de fauna silvestre y acuática por comunidades ye'kwana y sanema del alto río caura. In Investigación y Manejo de Fauna Silvestre en Venezuela en Homenaje al" Dr. Juhani Ojasti " (Ed. Machado-Allison). Simposio. Embajada de Finlandia. Caracas. Pp. 133-148.

36. Chacon RJ. 2012. Conservation or resource maximization: Analysis of subsistence hunting among the Achuar (shiwiar) of Ecuador. In The ethics of anthropology and amerindian research (Eds. Chacon RJ, Mendoza RG). Springer. Pp. 311-360. https://doi.org/10.1007/978-1-4614-1065-2_13

37. Chaves WA, Valle DR, Monroe MC, Wilkie DS, Sieving KE, Sadowsky B. 2017. Changing Wild Meat Consumption: An Experiment in the Central Amazon, Brazil. Conservation letters 11, 1-10. https://doi.org/10.1111/conl.12391

38. Chichón A. 2000. Fauna em la subsistencia de los Tsimane, Reserva de la Biosfera Estacion Biologica del Beni, Bolivia. Biodiversidad, Conservación y Manejo en la Región de la Reserva de la Biosfera Estación Biológica del Beni, Bolivia (Eds. Herrera-MacBryde O, Dallmeier F, MacBryde B, Comiskey JA, Miranda C). Smithsonian Institute. Pp. 365-384.

39. Clagget PR. 1998. The spatial extent and composition of wildlife harvests among three villages In the Peruvian Amazon. Paper to Meeting of the Latin American Studies Association. Chicago, IL.

40. Copa ME, Townsend WE. 2004. APROVECHAMIENTO DE LA FAUNA POR DOS COMUNIDADES TSIMANE': UN SUBSIDIO DEL BOSQUE A LA ECONOMÍA FAMILIAR. Revista Boliviana de Ecologia 16, 41-48

41. Costa JF, Ríos-Choronto R, Peña-Candia LZ, Simões E. 2018. Wild birds and mammals used by villagers from Lower Urubamba River at Cusco, Peru. Revista Peruana de Biologia 25, 463-470. http://dx.doi.org/10.15381/rpb.v25i4.15534

42. Cruz DR. 2011. Cambio cultural, economia e instituciones: Analises de la sostenibilidad de la actividad de cacería em la comunidad de la Ceiba, Rio Inídira (Guainía-Colômbia). TCC. Pontífica Universidad Javeriana.

43. Cuéllar RL, Noss AJ, Arambiza A. 2004. El registro de la cacería como base para el monitoreo y manejo de fauna en Isoso. Revista Boliviana de Ecologia 16, 29-40

44. Cueva R, Ortiz A, Jorgenson JP. 2004. Caceria de fauna silvestre em el área de amortiguamiento del parque nacional yasuni, amazonia ecuatoriana. Memorias: Mane jo de Fauna Silvestre em Amazonia y Lationamerica. Universidad Nacional de la Amazonia Peruana.

45. Damaceno, AB, Ortega, GP, Turci, LCB. 2019. Uso da caça de subsistência no assentamento Santa Luzia, Cruzeiro do Sul, Acre. PUBVET 13(2), 1-8. https://doi.org/10.31533/pubvet.v13n2a262.1-8

46. de la Montaña E. 2013. Cacería de subsistencia de distintos grupos indígenas de la Amazonia Ecuatoriana. Ecosistemas 22, 84-96. https://doi.org/10.7818/ECOS. 2013.22-2.13

47. Delgado JBB. 2019. Patrones de cacería y consumo de proteína animal en cuatro comunidades Waorani, Reserva de Biósfera Yasuní. Creatividad e desarollo 3, 43-60. https://doi.org/10.33262/cienciadigital.v3i3.2.1.781

48. Descola P. 1994. In the society of nature: A native ecology in Amazonia. Thesis. Cambridge.

49. Dias CJ. 2004. Na floresta onde vivem mansos e brabos: economia simbolica de acesso à natureza praticada na Reserva Extrativista do Alto Juruá - Acre. Thesis Unicamp. 265 p

50. El Bizri, Morcatty, Ferreira, Mayor, Vasconcelos Neto, Valsecchi, Nijman, Fa. 2014. Social and biological correlates of wild meat consumption and trade by rural communities in the Jutaí River basin, central Amazonia. Journal of Ethnobiology 40, 183-201. https://doi.org/10.2993/0278-0771-40.2.

51. Emidio-Silva C. 1998. A caça de subsistência praticada pelos índios Parakanã (sudeste do Pará): Características e sustentabilidade. Master Thesis. UFPA.

52. Endo W. 2005. Campinarana e índios Baniwa: Influências ambientais e culturais sobre a comunidade de vertebrados terrestres no alto rio negro, AM. Thesis. Instituto Nacional de Pesquisa da Amazônia/Universidade Federal do Amazonas.

53. Escobedo AJG, Rios CCR. 2003. Uso de la fauna silvestre, peces y de otros productos forestales no maderables em las comunidades de las etnias quechua y achuar del rio Huasaga, Loreto-Peru. Thesis. Universidad Nacional de la Amazonia Peruana.

54. Espinosa S, Branch LC, Cueva R. 2014. Road Development and the Geography of Hunting by an Amazonian Indigenous Group: Consequences for Wildlife Conservation. PLOS One 9, e114916. https://doi.org/10.1371/journal.pone. 0114916.

55. Espinosa SR. 2012. Road development, bushmeat extraction and jaguar conservation in yasuni biosphere reserve - ecuador. Thesis. University of Florida

56. Fang TG, Rios CC, Bodmer RE. 2004. Implementación de un programa piloto de certificación de pieles de pecaríes (Tayassu tajacu y T. pecari) en la comunidad de Nueva Esperanza, río Yavarí Miri. Memorias: Mane jo de Fauna Silvestre en Amazonia y Lationamerica. Universidad Nacional de la Amazonia Peruana. Iquitos.

57. Fank J, Porta, E. 1996. A vida social e econômica dos Suruwaha (OPAN).

58. Félix-Silva, D, Vidal, MD, Alvarez-Júnior, JB, Pezzuti, JCB. 2018. Caracterização das Atividades de Caça e Pesca na Floresta Nacional de Caxiuanã, Pará, Brasil, com Ênfase no Uso de Quelônios. Biodiversidade Brasileira 8, 232-250.

59. Ferreira DSS, Campos CEC, Araujo AS 2012. Aspectos da atividade de caça no assentamento rural Nova Canaã, município de Porto Grande, estado do Amapá. Biota Amazônica 2, 22-31. http://dx.doi.org/10.18561/2179-5746/biotaamazonia.v2n1p22-31

60. Ferrer A, Lew D, Vispo C, Daza F. 2013. Uso de la fauna silvestre y acuática por comunidades del bajo río Caura (Guayana venezolana). Biota Colombiana 14, 33-44.

61. Ferrer A, Romero V, Lew D. 2012. Consumo de fauna silvestre en el eje agrícola Guarataro, Reserva Forestal El Caura, Estado Bolívar, Venezuela. Memoria de la Fundación La Salle de Ciencias Naturales 173-174: 239-251

62. Figueiredo RA, Barros FB. 2015. "A comida que vem da mata": conhecimentos tradicionais e práticas culturais de caçadores na Reserva Extrativista Ipaú-Anilzinho. Fragmentos de cultura 25, 193-212. https://doi.org/10.18224/frag.v25i2.4181

63. Flores FJ, Cardenas KZ, Chacón OM, Beltran JS, Alfaro-Tapia A, Cayo JLJ, Sacaro JLV, Cashiri PC, Chinoa RY, Mendoza RK, 2023. Caceria para autoconsumo durante la pandemia de COVID-19: el caso de las comunidades Matsigenkas del Parque Nacional Manu. Revista Peruana de Biología 30, e24901. http://dx.doi.org/10.15381/rpb.v30i2.24901

64. Flores FJ. 2023. Caracterización y sostenibilidad de la caceria de las comunidades nativas matsiguenkas en el parque nacional del Manu. Dissertação de mestrado. Universidad Nacional de San Antonio Abad del Cusco.

65. Fonseca RA. 2011. Estratégias de caça de subsistência entre comunidades ribeirinhas tradicionais da Floresta Nacional do Tapajós, Pará, Brasil. Dissertação de Mestrado. Museu Paraense Emílio Goeldi. Belém, Pará, Brasil.

66. Fonseca RA. 2022. Ecologia e conservação de Podocnemis expansa (Testudines, Podocnemidae) no médio Tapajós, Brasil. Tese de Doutorado. Universidade Federal do Oeste do Pará. Santarém, Brasil.

67. Forline, L. C. The persistence and cultural transformation of the Awá-Guajá indians: foragers of Maranhão state, Brazil. 1997. Tese (Doutorado em Antropologia) – University of Florida, Gainesville, 1997.

68. Fragoso JMV, Santos MC. 2000. Wildlife and hunting practices of seringueiros in Seringal São Salvador. Report. Pesacre. Acre.

69. Francesconi W, Bax V, Blundo-Canto G, Wilcox S, Cuadros S, Vanegas M, Quinteros M, Torres-Vitolas C. 2018. Hunters and hunting across indigenous and colonist communities at the forestagriculture interface: an ethnozoological study from the Peruvian Amazon. Journal of Ethnobiology and Ethnomedicine 14:54. https://doi.org/10.1186/s13002-018-0247-2

70. Franzen M. 2001. Las pautas de cacería de los Huaorani: la reacción a la economía-mercado en la Amazonía del Ecuador. In Jorgenson JP, Rodriguez MC (ed.) Conservación y desarrollo sostenible del Parque Nacional Yasuní y su área de influencia. Memorias del Seminario-Taller 2001.

71. Franzen M. 2006. Evaluating the sustainability of hunting: a comparison of harvest profiles across three Huaorani communities. Environmental Conservation 33: 36-45. https:/doi.org/10.1017/S0376892906002712

72. Gaitán MB. 1999. Patrones de cacería em uma comunidad indígena ticuna em la amazonía colombiana. Eds. Fang TG, Montenegro OL, Bodmer RE. Instituto de Ecología, La Paz, Bolivia. Pp. 71-75.

73. Gavin MC. 2007. Foraging in the fallows: Hunting patterns across a successional continuum in the Peruvian Amazon. Biological Conservation 134, 64-72. https://doi.org/10.1016/j.biocon.2006.07.011

74. Gomes ASR. 2017. Automonitoramento Paiter Surui sobre o uso de mamíferos de médio e grande porte na Terra Indígena Sete de Setembro, Cacoal, Rondônia, Brasil. ECAM. https://www.paiter-surui.com/_files/ugd/584408_e8517ef3ac834ab493b99f508106704c.pdf

75. Gonzales J, Vanegas T, Valencia F, Valencia I. 2010 Investigacion participativa sobre la actividad de caceria de subsistencia: comunidad indigena de santa isabel vaupes, colombia. In Manejo do mundo: Conhecimentos e práticas dos povos indígenas do Rio Negro (Org. Cabalzar A). Instituto Socioambiental. Pp. 104-113.

76. Gonzales JA. 2003. Patrones generales de caza y pesca em comunidades nativas y asentamientos de colonos aledaños a la Reserva Comunal Yanesha, Pasco, Peru. In Manejo de fauna silvestre em Amazonia y Latinoamerica (Ed. Polanco-Ochoa R). CITES. Cartagena.

77. Greaves RD. 1997. Hunting and multifuncional use of arrow and bows. In Projectile Technology (Ed. Knecht H). Springer. Boston.

78. Guarín MDPT. 2010. Evaluación de la sostenibilidad de la cacería de mamíferos en la comunidad de zancudo, reserva nacional natural puinawai, guainía-colombia.Tese. Universidad Nacional de Colombia. Thesis. Universidad Nacional de Colombia.

79. Guedes AG. 1981. La fauna silvestre y su aprovechamiento por las comunidades campa del Rio Pichis. Revista Forestal del Peru 10:1-12

80. Guimarães, CDO, Palha, MDC, Tourinho, MM. 2019. Estratégias e dinâmica de caça na ilha de Colares, Pará, Amazônia Oriental. Biota Amazônia 9, 5-10. http://dx.doi.org/10.18561/2179-5746/biotaamazonia.v9n1p5-10

81. Hames RB, Vickers WT. 1982. Optimal diet breadth theory as a model to explain variability in Amazonian hunting. American Ethnologist 9, 352-378. https://doi.org/10.1525/ae.1982.9.2.02a00090

82. Hames RB. 1979. A Comparison of the Efficiencies of the Shotgun and the Bow in Neotropical Forest Hunting. Human Ecology 7, 219-252. https://doi.org/10.1007/BF00889493

83. Hammen MC. 1992. El Manejo del Mundo: Naturaleza y sociedad entre los Yukunas de Amazonia Colombiana. Studies in the Colombian Amazonia. Tropenbos. 376p.

84. Herrera JC. 1999. Uso de fauna silvestre durante la extraccion de palmito. Boletín BOLFOR 17.

85. Hiraoka M. 1995. Aquatic and land fauna management among the floodplain ribereños of the Peruvian Amazon. In The fragile tropics of Latin America: Sustainable management of changing environments (Eds. Nishizawa T, Uitto JI). United Nations University Press. Pp. 201-225.

86. Hodl W., Gashe J. (undated) Indian agriculture as exemplified by a secoya village in the rio Yubineto in Peru. In Bittner A. Applied geography and develpment. Volume 20. pp 20-31.

87. Holmes R, Clark K. 1992. Diet, acculturation and nutritional status in Venezuela's Amazon territory. Ecology of Food and Nutrition 27, 163-187.

88. Huallpa MMT. 2017. Densidad de mamiferos mayores em el rio La Novia, Purus-Ucayali y su relación com la presión de caza, Abril-Julio 2015. Thesis. Universidad Nacional de San Agustín de Arequipa. Peru.

89. Hurtado LFJ. 2020. Incidencia de la comercialización de carne de monte en la percepción de la disponibilidad de fauna y regulación cultural de la cacería en comunidades indígenas del nororiente de la Amazonia colombiana. Pontífica Universidad Javeriana, Bogota. Master Thesis.

90. Kaplan H, Kopischke K. 1992. Resource use, traditional technology, and change among native peoples of lowland south America. In Conservation of Neotropical forests (Eds. Redford KH, Padoch C). Pp 83-103

91. Kirkland M, Eisenber C, Bicerra A, Bodmer RE, Mayor P, Axmacher JC. 2018. Sustainable wildlife extraction and the impacts of socio-economic change among the KukamaKukamilla people of the Pacaya-Samiria National Reserve, Peru. Oryx 54:2-10. https:/doi.org/10.1017/S0030605317001922

92. Klebelsberg E. 2005. Evaluación del estado de la fauna de caza en áreas de uso de la Comunidad Nativa Mushukllacta de Chipaota, Zona de Amortiguamiento del Parque Nacional Cordillera Azul (PNCAZ), Perú. Informe Final del trabajo de tesis realizado con CIMA entre el 7 de Diciembre del 2004 y el 24 de Junio del 2005.

93. Knoop 2018. An Investigation into Primate Diversity and Livelihoods of the Maraguá people in Central Amazonia, Amazonas, Brazil. MSc Dissertation, Oxford Brookes University, UK.

94. Kroemer G. 1989. A caminho das malocas Zuruahá. Edições Loyola, São Paulo.

95. Leewenberg, F. 1995. Estudo e manejo de fauna silvestre (Reserva Indígena Ashaninca-Kaxinawá/Breu, AC). Centro de Pesquisa Indígena.

96. Linke IHVV. 2009. Caracterização do uso da fauna cinegética em aldeias das etnias Wayana e Aparai na TI Parque Indígena do Tumucumaque. Tese. Universidad Federal do Pará.

97. Lizot J. 1979. Reply to On food taboos and Amazon cultural Ecology. Current Anthropology 20, 150-151.

98. Loja-Alemán JF, Ascorra C. 2004. Implementación y monitoreo de planes de manejo de fauna silvestre en Tambopata. Manejo de fauna silvestre em Amazonia y Latinoamerica. Iquitos.

99. Lopes, KGM, Oliveira, AD. 2018. ASPECTOS NUTRICIONAIS DAS COMUNIDADES RURAIS NA AMAZÔNIA SUL-OCIDENTAL: ANÁLISE DA DIETA ALIMENTAR PROVENIENTE DA CAÇA DE SUBSISTÊNCIA DOS MORADORES DO SERINGAL QUIXADÁ, NO ESTADO DO ACRE. 70a Reunião Anual da SBPC, Alagoas, Brasil.

100. Lozano REP. 2012. Diagnóstico de la comercialización de fauna silvestre terrestre en los principales mercados de la ciudad de Pucallpa –Región Ucayali. Tese. Universidad Nacional de Ucayali.

101. Lu F. 2010. Patterns of indigenous resilience in the Amazon: A case study of Huaorani hunting in Ecuador. Journal of Ecological Anthropology 14, 5-21. http://dx.doi.org/10.5038/2162-4593.14.1.1

102. Luz A. C. 2012. The role of acculturation of indigenous peoples' hunting patterns and wildlife availability: The case of Tsimane' in Bolivian Amazon. Thesis. Universidad Autonoma de Barcelona.

103. Martins ES. 1993. A caça de subsistencia de extrativistas na Amazonia: sustentabilidade, biodiversidade e extinção de espécies. Univesidade de Brasília.

104. Martins MB. 2009. Distribuição e abundância da fauna na área de vida da comunidade de Pini, em lugares selecionados por caçadores da Floresta Nacional do Tapajós/Pará. Thesis. Universidad Federal do Amazonas.

105. Medeiros MFS, Garcia L. 2006. O consumo e as estratégias de caça utilizadas pelas populações tradicionais da Reserva Extrativista Chico Mendes. Interações 7,121-134.

106. Melo ERA. 2012. O impacto da caça sobre a comunidade de mamíferos de médio e grande porte em Novo Paraíso, Roraima, Norte da Amazônia Brasileira. Thesis. Universidade Federal de Pernambuco.

107. Melo NJH. 1998. Caracterizacion de los patrones de caceria em la comunidad de Aduche y el Assentamiento de Puerto Santander-Araracuara, médio Caquetá, Amazonia Colombiana. TCC. Pontifica Universidad Javeriana, Bogota.

108. Mena P, Cueva R. 2001. Cacería de subsistencia en tres comunidades de la zona de amortiguamiento del Parque Nacional Yasuní. In Jorgenson JP, Rodriguez MC (ed.) Conservación y desarrollo sostenible del Parque Nacional Yasuní y su área de influencia. Memorias del Seminario-Taller 2001.

109. Mena PV, Stallings JR, Regalado J, Cueva R. 2000. The Sustainability of current hunting practices by the Huaorani. In Hunting for Sustainability in Tropical Forests. Eds. Robinson JG, Bennett EL. Columbia University Press, New York, USA. Pp. 57-78.

110. Mesquita GP, Rodriguez-Treijeiro JD, Barreto LN. 2018. Patterns of Mammal Subsistence Hunting in Eastern Amazon, Brazil. Wildlife Society Bulletin 42, 272-283. https://doi.org/10.1002/wsb.873

111. Milton K. 1984. Protein and Carbohydrate Resources of the Maku Indians of Northwestern Amazonia. American Anthropologist 86, 7-27. https://doi.org/10.1525/aa.1984.86.1.02a00020

112. Miranda JR, Mangabeira JAC. 2002. Extrativismo animal em zona de fronteira agrícola na Amazônia: O caso de Machadinho D'Oeste - RO. Embrapa Monitoramento por Satélie, Documento 16.

113. Montenegro OL. 2004. Natural licks as keystone resources for wildlife and people in Amazonia. Thesis. University of Florida.

114. Moura ALB. 2013. Caça de subsistência e os mamíferos da resex do alto tarauacá, acre. Thesis. Universidade Federal do Acre.

115. Muñoz MO, Dimian NA, Hurtado LFJ, Tunjano SS, Colmenares AB, Duque NR. 2014. La despensa del Tiquié: Diagnóstico y manejo comunitario de la fauna de consumo en la Guayana colombiana. Instituto Amazónico de Investigaciones Científicas “SINCHI”. Bogotá, D.C.

116. Naughton Treves S. 2002. Wild animals in the garden: conserving wildlife in Amazonian agroecosystems. AAA Geographers 9 488-506. https://doi.org/10.1111/1467-8306.00301

117. Navarro, JGG. 2004. Aprovechamiento de la fauna silvestre en comunidades cashinahua del río curanja y purus. Informe Tecnico I. Report. WWF.

118. Nunes, VS, Miranda JR, Barbosa MFS. 1997. Game hunting by rubber tappers of the tejo river basin, Acre state, Brazil. Embrapa. Grupo de Estudos Ecológicos 5, 1-15.

119. Ohl-Schacherer J, Shepard GH, Kaplan H, Peres CA, Yu DW. 2007. The Sustainability of Subsistence Hunting by Matsigenka Native Communities in Manu National Park, Peru. Conservation Biology 21, 1174-1185. https://doi.org/10.1111/j.1523-1739.2007.00759.x

120. Ojasti J, Fajardo G, Cova M. 1983. Consumo de fauna por una comunidad indígena en el Estado Bolívar, Venezuela. In Conservación y manejo de la fauna silvestre en Latinoamérica (Ed. Aguilar PG). Apeco, Arequipa; 45-50

121. Oliveira MA. 2012. Efeitos da caça de subsistência sobre a comunidade de mamíferos de uma reserva extrativista na amazônia sul-ocidental. Thesis. Universidade Federal do Acre.

122. Oliveira, T. G., Gerude, R. G., Dias, P. A., & Resende, L. B. 2011. Utilização de caça pelos índios Awá/Guajá e Ka’apor da Amazônia maranhense. In: M. B. Matin, & T. G. Oliveira (Eds.),

Amazônia Maranhense: diversidade e conservação. pp. 271–282. Belém: Museu Paraense Emílio Goeldi.

123. Ortega-Rincón MC. 2014. ECOLOGICAL SUSTAINABILITY OF MAMMAL HUNTING IN INÍRIDA REGION, COLOMBIAN AMAZON. Msc Thesis. Universidad Nacional de Colombia

124. Ouboter PE. 2023. Biological and socio-economic background of Hunting in Suriname: a review. Academic Journal of Suriname, 14(1), 1-32.

125. Pacheco V, Amanzo J. 2003. Análisis de datos de cacería en las comunidades nativas de pikiniki y nuevo belén, río alto purús. In Alto Purus: Biodiversidad, conservacion y manejo (Eds. Pitman RL, Pitman N, Alvarez P). Center for Tropical Conservation. Pp. 217-225.

126. Parry L, Barlow J, Peres AC. 2009. Allocation of hunting effort by Amazonian smallholders: implications for conserving wildlife in mixed-use landscapes. Biological Conservation 142, 1777-1786. https://doi.org/10.1016/j.biocon.2009.03.018

127. Payan E. 2007. Measuring impact and sustainability of Amazon hunting in Colombia. Report. University College of London.

128. Peres, CA, Nascimento HS. 2006. Impact of game hunting by the Kayapó of south-eastern Amazonia: implications for wildlife conservation in tropical forest indigenous reserves. In: Hawksworth, D.L., Bull, A.T. (eds) Human Exploitation and Biodiversity Conservation. Topics in Biodiversity and Conservation, vol 3. Springer, Dordrecht. https://doi.org/10.1007/978-1-4020-5283-5_16

129. Peres-Peña P, Puerta LR, Grandez AE, Lujan JGM. 2014. Plan de maestro de animales de caza em la Reserva Nacional de Pucacuro. SERNANP.

130. Perez-Peña P, Gonzales-Tanchiva C, Trigoso-Pinedo M. 2016. Evaluacion del plan de manejo de animales de caza em la reserva nacional pucacuro. Folia Amazonica 25, 1-16. https://doi.org/10.24841/fa.v25i1.377

131. Pezzuti J, Chaves RP. 2009. Etnografia e manejo de recursos naturais pelos índios Deni, Amazonas, Brasil. Acta Amazonica 39, 121-138. https://doi.org/10.1590/S0044-59672009000100013

132. Pezzuti JCB, Rebelo GH, Silva DF, Lima JP, Ribeiro MC. 2004. A caça e a pesca no Parque Nacional do Jaú. In Janelas para a Biodiversidade no Parque Nacional do Jaú (Eds. Borges SH, Iwanaga S, Durigan CC, Pinheiro M) Fundação Vitória amazônica, Manaus. Pp. 213-230.

133. Pierret PV, Dourojeanni MJ. 1966. La caza y la alimentacion humana em las ribeiras del rio Pachitea, Peru. Turrialba 16, 271-277.

134. Pierret PV, Dourojeanni MJ. 1967. Importancia de la caza para alimentacion humana em el curso inferior del rio Ucayali, Peru. Revista Forestal del Peru 1, 2. https://doi.org/10.21704/rfp.v1i2.834

135. Politis GG. 2007. Nukak: ethnoarcheology of an amazonian people. Universty College of London.

136. Portilla IAS. 2016. Comparación de los perfiles de cacería en tres comunidades Waorani del Parque Nacional Yasuní entre el periodo 2002 y 2015. Thesis. Pontífica Universidad Católica del Ecuador.

137. Prado, H. M., Forline, L. C., Kipnis, R. 2012. Hunting practices among the Awá-Guajá: towards a long-term analysis of sustainability in an Amazonian indigenous community. Bol. Mus. Para. Emílio Goeldi. Cienc. Hum., 7(2): 479-491. https://doi.org/10.1590/S1981-81222012000200010

138. ProPurus, sem data. Evaluación del Uso de los Recursos Naturales en Cuatro Comunidades Nativas de la Provincia de Purús - Ucayali, 2009-2011

139. Puertas PE, Pinedo A, Soplin S, Antunes M, Lopez L, Caro J, Chicave L, Panduro R, Vasquez R, Flores JL. 2017. Evaluación poblacional y uso sostenible de animales de caza por comunidades indígenas en el área de conservación regional ampiyacu apayacu, noreste de la amazonía peruana. Folia Amazonica 26, 37-50. https://doi.org/10.24841/fa.v26i1.417

140. Ramos RM. 2005. Estratégia de caça e uso de fauna na reserva extrativista do Alto Juruá-AC. Thesis. Universidade de São Paulo.

141. Ramos RM. 2013. Caça de subsistência e conservação na amazônia (reserva extrativista rio xingu, terra do meio, pará): ecologia da caça e avaliação de impactos na fauna. Thesis. Universidade de Brasília.

142. Ramos-Rodríguez MC, Pérez-Peña PE, Angulo-Perez NC. 2023. Aprovechamiento de recursos naturales. En: Biodiversidad en la cuenca baja del Putumayo, Perú. Pérez-Peña P. E., Zárate-Gómez R., Ramos-Rodríguez M. C. y Mejía K. (Eds.). Instituto de Investigaciones de la Amazonía Peruana (IIAP), Iquitos-Perú. 136-156 pp.

143. Rangel AMP. Undated. Importancia de la fauna silvestre en la etnia Sikuani, comunidad de Cumarianae, selva de Matavén, Vichada, Colombia. Thesis. Universidad Sergio Arboleda.

144. Read J, Fragoso JMV, Luzar J. 2013. Wowetta Community Atlas. Project Fauna.

145. Read JM, Fragoso JMV, Kirsten KM, Luzar J, Overman H, Cummings A, Giery ST, Oliveira LF. 2010. Space, place, and hunting patterns among indigenous peoples of the Guyanese Rupununi region. Journal of Latin American Geography 9, 213-243

146. Redford KH, Stearman AM. 1989. Local peoples and the Beni Biosphere Reserve, Bolivia. Vida Sylvestre Neotropical 2, 49-56.

147. Reis YS, Valsecchi J, Queiroz H. 2018. Caracterização do Uso da Fauna Silvestre para Subsistência em uma Unidade de Conservação no Oeste do Pará. Biodiversidade Brasileira 8, 187-202.

148. Reyes CJ, Bodmer RE, Garcia J, Diaz D. 2001. Presión de caza y bases para el manejo de fauna com participación comunitaria em la Reserva Nacional Pacaya-Samiria. In Manejo de fauna com comunidades rurales (Eds. Rozo CC, Ulloa A, Torgler HR).

149. Ribeiro, ASS, Palha, MDC, Tourinho, MM, Whiteman, CW, Silva, ASL. 2007. Utilização dos recursos naturais por comunidades humanas do Parque Ecoturístico do Guamá, Belém, Pará. Acta Amazonica 37(2), 235-240. https://doi.org/10.1590/S0044-59672007000200009

150. Rios GR, Puerta LR, Peres-Peña P. 2013. Reserva Naiconal Pucacuro: Diagnóstico del processo de elaboración del plan maestro 2013-2018. Ministerio del Ambiente. Peru.

151. Riveros TAB. 2014. Usos, prácticas e ideologías socioculturales de la cacería de dos comunidades Tikuna, ubicadas em el sur de la Amazonía colombiana. Thesis. Universidad Nacional de Colombia. Letícia.

152. Rodríguez A. 2006. Cacería en la comunidad Guayabal-Anapo a partir del uso indígena del territorio en el gran resguardo Selva del Matavén, Vichada. TCC. Pontificia Universidad Javeriana. Bogota.

153. Rodriguez AMM. 2010. The impact of subsistence hunting by Tikunas on game species in Amacayacu National Park, Colombian Amazon. Thesis. Oxford Brooks University.

154. Rodriguez CA, van Hammen MC. 1999. Non-timber forest products and indigenous management of the forest along the middle Caquetá river: Complementary and new options

155. Lorini, H. (2006). Siguiendo huellas en el monte: monitoreo participativo de fauna y cacería en el Madidi. Conservación Internacional-Bolivia. La Paz, Bolivia. 70 p.

156. Romanoff AS. 1984. Matsés Adaptations in the Peruvian Amazon. Ph.D. dissertation. Columbia University, New York

157. Rosas GKC, Drumond PM. 2007. Caracterização da Caça de Subsistência em Dois Seringais Localizados no Estado do Acre (Amazônia, Brasil). Documentos 109. Embrapa.

158. Rumiz D, Guinat D, Sloar LR, Herrera FJ. 2001. Logging and hunting in community forests and coorporate concessions: two contrasting case studies in Bolivia. In: The cutting edge: Conserving wildlife in logged tropical forests (Eds. Fimbel RA, Grajal A, Robinson JG). Columbia University Press.

159. Rumiz D, Painter L, Wallace R, Guinart D, Herrera JC. 1998. La Fauna Silvestre y sus Recursos: ¿Qué Sabemos y Cómo Podemos Aplicarlo al Manejo Sostenible de Bosques en Bolivia? Simposio Internacional "Posibilidades de Manejo Forestal Sostenible en América Tropical". Bolfor. Bolívia. Pp. 63-97.

160. Rumiz DI, Maglianesi MA. 2001. Hunting impacts associated with Brazil nut harvesting in the Bolivian Amazon. Vida Silvestre Neotropical 10, 19-29.

161. Saffirio G, Scaglion R. 1982. Hunting efficiency in acculturated and unacculturated yanomama villages. Journal of Anthropological Research 38, 315-327.

162. Salas MPM, Arevalo HFL, Palomino PS. 2016. Cacería de subsistencia de mamíferos en el sector oriental de la reserva de biósfera El Tuparro, vichada (Colombia). Acta Biológica Colombiana 21, 151-166. https://doi.org/10.15446/abc.v21n1.49882

163. Saldaña JSR, Saldaña VLH. 2011. La cacería de animales silvestres en la comunidad de Bretaña, río Puinahua, Loreto – Perú. Revista Colombiana de Ciência Animal 3, 225–237. https://doi.org/10.24188/recia.v3.n2.2011.369

164. Sanchez A, Vasquez P. 2007. Presión de caza de la comunidad nativa mushuckllacta de chipaota, zona de amortiguamiento del Parque Nacional Cordillera Azul, Perú. Ecologia Aplicada 6, 131-138.

165. Santos S. M. 2022. Pesquisa sobre caça na região Uaçá. In Lewkowicz RB (org) Olhares sobre o território: Pesquisas dos agentes ambientais indígenas do Oiapoque. Iepé - Instituto de Pesquisa e Formação Indígena. São Paulo. pp: 90-96

166. Santos Junior MA, Iwanaga S, Borges SH, Moreira MP, Durigan CC, Saldanha F (2018) SiMUR: Uma experiência participativa do monitoramento do uso de recursos naturais. Technical Report. Fundação Vitória Amazônica. Manaus, Brazil.

167. Saquiray MGI, Gonzales DG. 2007. Aprovechamiento de la fauna silvestre por los Kandozi, Quebrada Chapuri, Rio Pastaza. Thesis.

168. Schulte-Herbrüggen B, Rossiter H. 2003. Project Las Piedras A socio-ecological investigation into the impact of illegal logging activity in Las Piedras, Madre de Dios, Peru. The University of Edinburg.

169. Sena, RF, Oliveira, MA, Romagnoli, FC, Costa-Rodrigues, APV. 2021. Uso da fauna e flora por comunidades quilombolas do arquipélago do Marajó, Pará. Etnoscientia 3, 98-115. http://dx.doi.org/10.18542/ethnoscientia.v6i3.10502

170. Severino, WMS, Santos, ES, Albuquerque-Cunha, HF. 2021. Hydropower plant in Eastern Amazon and its impacts on the local population's eating habits. Revista Ambiente & Água 16(5), e2700. https://doi.org/10.4136/ambi-agua.2700

171. Shaffer CA, Milsten MS, Yakuma C, Marauanru E, Suse P. 2017. Sustainability and comanagement of subsistence hunting in an indigenous reserve in Guyana. Conservation Biology 31, 1119–1131. https://doi.org/10.1111/cobi.12891

172. Sheppard, SN. 2006. Evaluación y monitoreo del aprovechamiento de los recursos hidrobiológicos y de fauna terrestre en comunidades indígenas del purús. Technical Report. WWF.

173. Sheppard, SN. 2007a,b. Sheppard, SN. 2007a - Evaluación y monitoreo del aprovechamiento de los recursos hidrobiologicos y de fauna terrestre en comunidades indígenas de la cuenca del purús – ucayali, perú. Informe Técnico II. WWF.Sheppard, SN. 2007b - Evaluación y monitoreo del aprovechamiento de los recursos hidrobiologicos y de fauna terrestre en comunidades indígenas de la cuenca del purús – ucayali, perú. Informe Técnico III. WWF.

174. Silva AFS. 2008. O uso da fauna cinegética e o consumo de proteína animal em comunidades rurais na Amazônia oriental: Reserva Extrativista Tapajós-Arapiuns. Tese. Universidade Federal do Pará.

175. Silva, AB, Pereyra, PER, El Bizri, HR, Souto, WMS, Barboza, RSL. 2022. Patterns of wildlife hunting and trade by local communities in eastern Amazonian floodplains. Ethnobiology and Conservation 11, 16. https://doi.org/10.15451/ec2022-07-11.16-1-19

176. Silverwood-Cope PL. 1990. Os Makú: povo caçador do noroeste da Amazônia. UnB.

177. Sirén AH. 2004. Changing interactions between humans and nature in Sarayaku, Ecuadorian Amazon. PhD. University of Agricultural Sciences, Uppsala, Suecia.

178. Siskind J. 1973. To hunt in the morning. Galaxy Books.

179. Smith NJH. 1976. Utilization of game along Brazil's transamazon highway. Acta Amazonica 6, 455-466. https://doi.org/10.1590/1809-43921976064455

180. Sousa GS. 2015. Caça e segurança alimentar em comunidades ribeirinhas do médio xingu. Tese. Universidade Federal do Pará.

181. Souto T, Nuñez C, Palomino R, Deichmann JL, Alonso A. 2013. Uso de recursos naturales por 10 comunidades mestizas del rio Tapiche, Peru. In Biodiversidad y uso de recursos naturales en la cuenca baja del río Tapiche, Loreto, Perú (Eds. Palomino LR, Diechmann JL, Alonso A). Technical Report 31. Iquitos, Peru.

182. Souza-Mazurek RR, Pedrinho T, Feliciano X, Hilário W, Gerôncio S, Marclo E. 2000. Subsistence hunting among the Waimiri Atroari Indians in central Amazonia, Brazil. Biodiversity and Conservation 9, 579-596. https://doi.org/10.1023/A:1008999201747

183. Stearman AM. 1990. The effects of settler incursion on fish and game resources of the Yuquí, a native Amazonian Society of Eastern Bolivia. Human Organization 49, 373-385. https://doi.org/10.17730/humo.49.4.906547u862h5x566

184. Strong JN, Fragoso JMV, Oliveira LFB. 2010. Padrões de uso e escolha de caça pelos índios macuxi em roraima. In Roraima: homem, ambiente e ecologia (Ed. Barbosa RI). FEMACT.

185. Tamayo ZJS. 2017 Impacto de la caceria em quatro espcies de fauna silvestre efectuadas por comunidades locales em la zona de aprovechamiento directo de la reserva nacional matses, loreto Peru. Thesis. Universidad Nacional de la Amazonia Peruana.

186. Terra AK. 2007. A caça de subsistência na reserva de desenvolvimento sustentável piagaçu-purus e na terra indígena lago ayapuá, amazônia central, brasil. Thesis. Universidade Federal do Amazonas.

187. Townsend WR. 2000. The Sustainability of Subsistence Hunting by the Sirionó Indians of Bolivia. In Hunting or sustainability in tropical forests (Eds. Robinson J, Bennett EL).

188. Tregidgo D. 2016. Fishing and hunting in the Amazon floodplain: linkages among biodiversity conservation, rural livelihoods and food security. PhD Lancaster University.

189. Trinca CT, Ferari SF. 2006. Caça em assentamento rural na Amazônia mato-grossense. Diálogos em ambiente e sociedade no Brasil. ANPPAS.

190. Tuesta JC. 2012. Impacto de la caza sobre la población de animales silvestres, en la zona de aprovechamiento directo y amortiguamiento del área de conservación regional comunal tamshiyacu- tahuayo (acrctt) loreto – perú. TCC. Universidad Nacional de la Amazonia Peruana.

191. Tuesta ZEE. 2012. Caza y comercializacion de fauna silvestre em la region de Ucayali. Investigación Universitária 6, 81-100.

192. Vadjunec JM, Rocheleau D. 2009. Beyond forest cover: land use and biodiversity in rubber trail forests of the Chico Mendes Extractive Reserve. Ecology and Society 14, 29.

193. Vanegas M. 2006. Caracterización de la cacería y su importancia en la seguridad alimentaria familiar de una comunidad indígena Piaroa en el Resguardo Unificado Selva de Matavén, Vichada Colombia. TCC. Pontífica Universidad Javeriana.

194. Vargas LA. 2011. Manejo comunitario de fauna silvestre en las comunidades vecinas al Parque Nacional Noel Kempff Mercado. Technical Report. Bolfor. Bolivia.

195. Vickers W. 1994. From opportunism to nascent conservation: The Case of the Siona-Secoya. Human Nature 5, 307-337. https://doi.org/10.1007/BF02734164

196. Vivan J. 2005. Etnozoneamento TI Mamoadate. Rio Branco, AC.

197. Watanabe J, Pequeño T, Martinez J, Gavin M. 2004. Uso de fauna silvestre por las comunidades de la zona de amortiguamiento del Parque Nacional Cordillera Azul, Perú. Manejo de fauna silvestre em Amazonia y Latinoamerica. Iquitos.

198. Watanabe J. 2006. Manejo comunal de fauna silvestre en el Parque Nacional Cordillera Azul, San Martín – Perú. Biotempo 6, 38-45. https://doi.org/10.31381/biotempo.v6i0.881

199. Winter KA. 2002. Subsistence use of terrestrial and aquatic animal resources in the tierra comunitaria de origen itonama of lowland Bolivia. PhD. University of Georgia

200. Yost JA, Kelley PM. 1983. Shotguns, blowguns, and spears: the analysis of technological efficiency. In Adaptive Response of Native Amazonians (Ed. Vickers WT). Academic Press, New York, USA. Pp. 189-224.

201. Zapata-Ríos G, Urgilés C, Suárez E. 2009. Mammal hunting by the Shuar of the Ecuadorian Amazon: is it sustainable? Oryx 43, 375-385. https:/doi.org/10.1017/S0030605309001914

202. Zapata-Ríos G. 2001. Sustentabilidad de la cacería de subsistencia: el caso de cuatro comunidades Quichuas en la Amazonía nororiental ecuatoriana. Mastozoología Neotropical 8, 59-66.

203. Zumba FRS. 2018. A pressão de caça por indígenas e não indígenas no Parque Estadual Chandless (AC). Tese de Mestrado UFAC.

Supplementary Data 5. Estimated densities (individuals per km^2) for 139 hunted taxa recorded in this study.

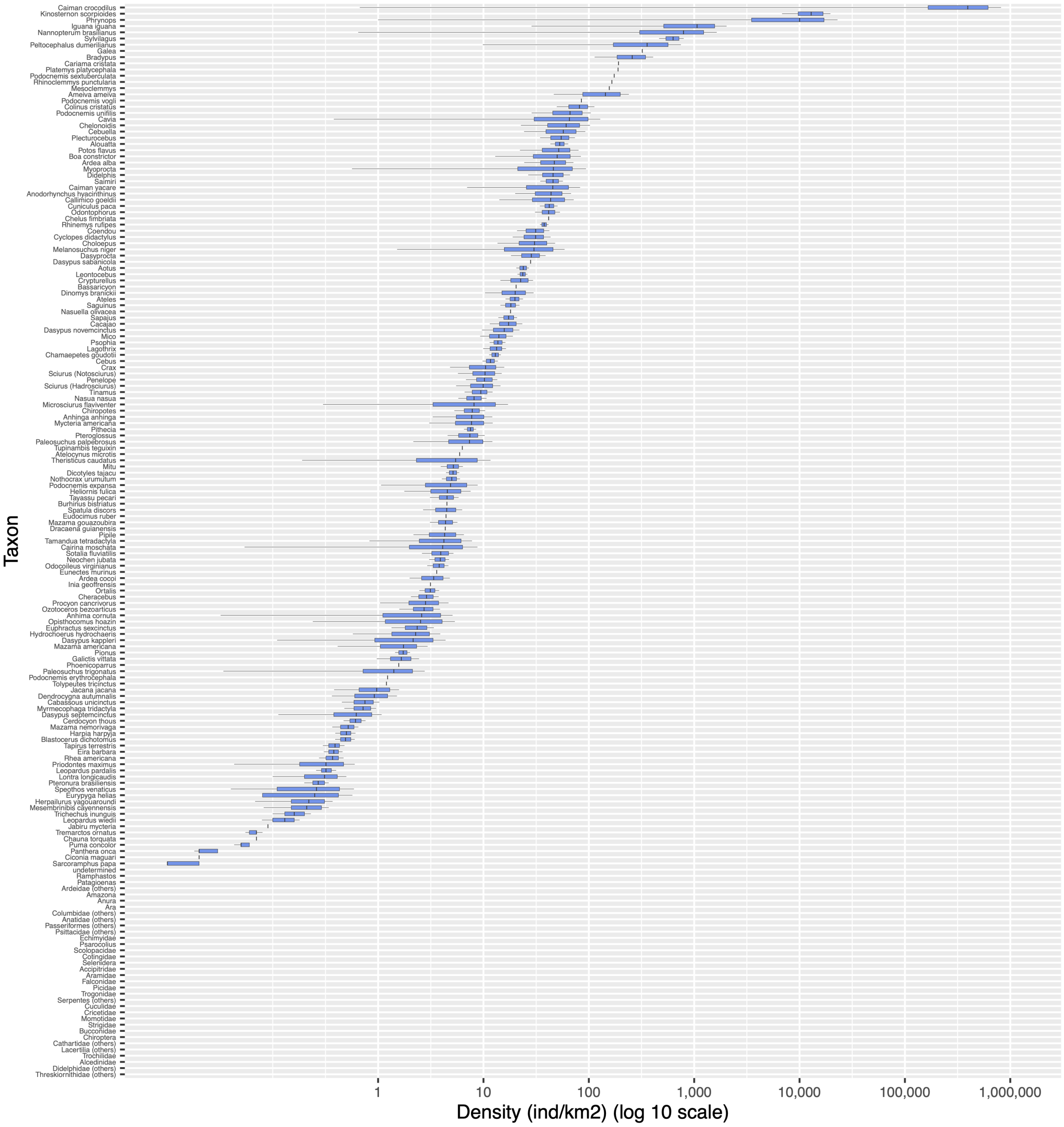

Supplementary Table 9. Mean, standard deviation, and sample size of energy, macro- and micronutrients per 100 g of wild meat in 26 hunted Amazonian species.

Taxon	Calories (Kcal)	Protein (g)	Total fat (g)	Iron (mg)	Zinc (mg)	Selenium (µg)	Vitamin B1 (mg)	Vitamin B2 (mg)	Vitamin B3 (mg)	Vitamin B12 (µg)
Caiman crocodilus	91.6 (1)	21.9 (1)	0.46 (1)	–	–	–	–	–	–	–
Caiman sp.	108 (1)	21 ± 2.3 (3)	3.17 ± 2.07 (3)	1 (1)	–	–	0.04 (1)	0.18 (1)	2.8 (1)	–
Cairina moschata	126 (1)	23.7 (1)	2.7 (1)	1.8 (1)	–	–	0.26 (1)	0.26 (1)	5 (1)	–
Cavia aperea	116 (1)	26.3 (1)	0.4 (1)	1.9 (1)	–	–	0.07 (1)	0.21 (1)	4 (1)	–
Chelonoidis denticulatus	114 ± 2.12 (2)	18 ± 4.74 (2)	1.04 ± 1.07 (2)	1 ± 0.42 (2)	0.95 (1)	–	0.22 ± 0.03 (2)	0.39 ± 0.08 (2)	1.56 ± 2.03 (2)	–
Cuniculus paca	109 ± 10.1 (4)	21.8 ± 3.47 (5)	1.75 ± 0.63 (4)	1.67 ± 0.40 (3)	0.71 ± 0.03 (2)	–	0.23 ± 0.18 (3)	0.29 ± 0.13 (3)	2.36 ± 3.59 (3)	–
Dasyprocta sp.	93.9 (1)	19.3 (1)	1.21 (1)	–	–	–	–	–	–	–
Dasypus novemcinctus	162 ± 4.24 (2)	28.6 ± 0.58 (2)	4.88 ± 0.73 (2)	6.01 ± 6.92 (2)	3 (1)	12.9 (1)	–	–	–	0.7 (1)
Dicotyles tajacu	123 ± 31 (5)	20.5 ± 2.94 (10)	4.91 ± 3.17 (9)	1.95 ± 0.21 (2)	0.82 (1)	–	0.30 ± 0.12 (2)	0.21 ± 0.14 (2)	2.06 ± 2.74 (2)	–
Hydrochoerus hydrochaeris	115 ± 17.5 (3)	22.2 ± 1.39 (6)	1.93 ± 1.38 (6)	2.7 (1)	–	–	–	–	–	–
Iguana iguana	106 (1)	22.6 ± 2.55 (2)	2.2 ± 1.83 (2)	2.67 ± 1.04 (2)	2.53 (1)	–	–	–	–	–
Mazama sp.	131 ± 23.2 (5)	25.6 ± 5.39 (6)	1.74 ± 1.29 (5)	2.43 ± 0.92 (3)	–	–	0.14 ± 0.08 (3)	0.39 ± 0.08 (3)	8.77 ± 2.14 (3)	–
Nasua nasua	274 (1)	14.5 (1)	23.5 (1)	3.6 (1)	–	–	0.04 (1)	0.08 (1)	1.8 (1)	–
Odocoileus virginianus	193 (1)	28.1 (1)	8.28 (1)	3.8 (1)	3.37 (1)	12.4 (1)	–	–	–	2.92 ± 1.95 (6)
Patagioenas sp.	279 (1)	18.6 (1)	22.1 (1)	1.8 (1)	–	–	0.1 (1)	0.28 (1)	5.3 (1)	–
Peltocephalus dumerilianus	105 (1)	16.8 (1)	3.53 (1)	–	–	–	–	–	–	–
Podocnemis expansa	86.9 ± 6.56 (4)	19.2 ± 2.56 (5)	2.12 ± 2.05 (5)	1.2 ± 0.46 (3)	–	–	0.02 (1)	0.09 (1)	5.25 (1)	–
Podocnemis sextuberculata	111 (1)	23.8 (1)	5.56 (1)	–	–	–	–	–	–	–
Podocnemis unifilis	96.3 (1)	20.3 (1)	1.68 (1)	–	–	–	–	–	–	–
Rhea americana	–	–	1.23 ± 0.08 (2)	–	–	–	–	–	–	–
Salvator merianae	–	23.6 (1)	4 (1)	–	–	–	–	–	–	–
Tapirus terrestris	127 (1)	22.1 (1)	3.54 (1)	–	–	–	–	–	–	–
Tayassu pecari	96 ± 8.49 (2)	22.6 ± 3.32 (2)	2.34 ± 1.79 (2)	–	–	–	–	–	–	–
Tolypeutes tricinctus	172 (1)	29 (1)	5.4 (1)	10.9 (1)	–	–	0.1 (1)	0.4 (1)	6 (1)	–
Tupinambis teguixin	112 (1)	24.4 (1)	0.9 (1)	3.4 (1)	–	–	0.05 (1)	0.24 (1)	8.2 (1)	–
Zenaida auriculata	279 (1)	18.6 (1)	22.1 (1)	1.8 (1)	–	–	0.1 (1)	0.24 (1)	5.6 (1)	–

Supplementary Table 10. Daily values of Acceptable Macronutrient Distribution Range (AMDR), Estimated Average Requirement (EAR), Adequate Intake (AI), and Recommended Dietary Allowances (RDA) for protein, vitamin, and minerals per life stage group. Source: IOM (1998[90], 2002[91], 2005[92]).

Life Stage Group	Protein in g/d (AI*, RDA)	Total fat in % (AMDR) or g/d (AI*)	Iron in mg/d (EAR or AI*)	Zinc in mg/d (EAR or AI*)	Selenium in mcg/d (AI* or EAR)	Vitamin B1 in mg/d (AI* or EAR)	Vitamin B2 in mg/d (AI* or EAR)	Vitamin B3 in mg/d (AI* or EAR)	Vitamin B12 in mcg/d (EAR or AI*)
Babies									
0-6 mo	9.1*	31*	0.27*	2*	15*	0.2*	0.3*	2*	0,4*
7-12 mo	11	30*	6.9	2.5	20*	0.3*	0.4*	4*	0,5*
Children									
1-3 y	13	30-40	3	2.5	17	0.4	0.4	5	0.7
4-8 y	19	25-35	4.1	4	23	0.5	0.5	6	1.0
Males									
9-13 y	34	25-35	5.9	7	35	0.7	0.8	9	1.5
14-18 y	52	25-35	7.7	8.5	45	1	1.1	12	2.0
19-30 y	56	20-35	6	9.4	45	1	1.1	12	2.0
31-50 y	56	20-35	6	9.4	45	1	1.1	12	2.0
51-70 y	56	20-35	6	9.4	45	1	1.1	12	2.0
>70 y	56	20-35	6	9.4	45	1	1.1	12	2.0
Females									
9-13 y	34	25-35	5.7	7	35	0.7	0.8	9	1.5
14-18 y	46	25-35	7.9	7.3	45	0.9	0.9	11	2.0
19-30 y	46	20-35	8.1	6.8	45	0.9	0.9	11	2.0
31-50 y	46	20-35	8.1	6.8	45	0.9	0.9	11	2.0
51-70 y	46	20-35	5	6.8	45	0.9	0.9	11	2.0
>70 y	46	20-35	5	6.8	45	0.9	0.9	11	2.0
Pregnancy									
<18 y	71	20-35	23	10.5	49	1.2	1.2	14	2.2
19-30 y	71	20-35	22	9.5	49	1.2	1.2	14	2.2
31-50 y	71	20-35	22	9.5	49	1.2	1.2	14	2.2
Lactation									
<18 y	71	20-35	7	10.9	59	1.2	1.3	13	2.4
19-30 y	71	20-35	6.5	10.4	59	1.2	1.3	13	2.4
31-50 y	71	20-35	6.5	10.4	59	1.2	1.3	13	2.4

Supplementary Table 11. Daily values for energy per life stage based on Estimated Energy Requirements (EER). Source: IOM (2005)[92]

Life Stage Group	Energy, kcal/day, active individuals	
	Female	Male
0-6 mo	520 (3 mo)	570
7-12 mo	676 (9 mo)	743
1-2 y	992 (24 mo)	1046
3-8 y	1642 (6 y)	1742
9-13 y	2071 (11 y)	2279
14-18 y	2368 (16 y)	3152
>18 y	2403* (19y)	3067*
Pregnancy		
14 – 18 y		
1st trimester	2368 (16 y)	
2nd trimester	2708 (16 y)	
3rd trimester	2820 (16 y)	
19 – 50 y		
1st trimester	2403 (19 y)	
2nd trimester	2743 (19 y)	
3rd trimester	2855 (19 y)	
Lactation		
14 – 18 y		
1st 6 mo	2698 (16 y)	
2nd 6 mo	2768 (16 y)	
19 – 50 y		
1st 6 mo	2733 (19 y)	
2nd 6 mo	2803 (19 y)	

* Subtract 10 calories per day for men and 7 calories per day for women for each year above 19 years old.

Supplementary Method 6. Formal endorsement by the Coordination of Indigenous Organizations of the Brazilian Amazon (COIAB) for the ethical aspects of primary data collection, article content, and participation in the Evaluation Committee for future research utilizing the Marupiara Dataset.

Movimento Indígena do Acre

Carta Nº 354/SEC/COIAB/2025

Manaus-AM, 16 de junho de 2025.

Prezado Editor e revisores da Nature,

Prezados,

A Coordenação das Organizações Indígenas da Amazônia Brasileira (COIAB), que representa nove organizações indígenas de cento e oitenta, povos da Amazônia Brasileira, expressa seu apoio à publicação do artigo "Healthy forests safeguard traditional wild meat food systems in Amazonia". Este estudo é de grande relevância, pois analisa dados sobre a caça praticada por povos indígenas e comunidades tradicionais na Amazônia.

Estudos como este, que destacam a importância do uso e manejo sustentável da fauna para nossos povos, estão em total alinhamento com nossos esforços para preservar a relação tradicional que mantemos com a natureza. Os resultados desta pesquisa têm o potencial de fortalecer nosso debate em escalas nacional e amazônica, reforçando a necessidade de proteção de nossos territórios para a continuidade de nossas práticas tradicionais sustentáveis, essenciais para a segurança alimentar e o bem-estar nutricional de nossos povos.

Adicionalmente, comprometemo-nos a colaborar com os autores do artigo e outras organizações representativas de povos indígenas e comunidades tradicionais na formação de um Comitê de Avaliação. Este grupo será responsável por analisar eventuais solicitações de pesquisadores externos para acesso aos dados de caça compilados no Banco de Dados mencionado no estudo, assegurando que quaisquer compartilhamentos sejam feitos de forma ética e respeitosa.

Agradecemos a oportunidade de contribuir para este diálogo científico e reafirmamos nosso compromisso com a produção de conhecimento que valorize as práticas indígenas e tradicionais.

Atenciosamente,

Elcio Severino Da Silva Manchineri
Assinado digitalmente na ZapSign por
Elcio Severino da Silva Manchineri
Data: 16/06/2025 16:12:00.650 (UTC-0300)

Elcio Severino da Silva Machineri
Coordenador Geral da COIAB

Alcebias Mota Constantino
Assinado digitalmente na ZapSign por
Alcebias Mota Constantino
Data: 16/06/2025 16:12:24.441 (UTC-0300)

Alcebias Mota Constantino
Vice-Coordenador Geral da COIAB

Marciely Ayap Tupari
Assinado digitalmente na ZapSign por
Marciely Ayap Tupari
Data: 16/06/2025 16:12:48.235 (UTC-0300)

Marciely Ayap Tupari
Coordenadora Secretária da COIAB

Dineva Maria Kayabi
Assinado digitalmente na ZapSign por
Dineva Maria Kayabi
Data: 16/06/2025 16:13:12.806 (UTC-0300)

Dineva Maria Kayabi
Coordenadora Tesoureira da COIAB

CNPJ: 63.692.479/0001-94 | ENDEREÇO: AV. AYRÃO, 235 CEP: 69025-050 - PRES. VARGAS, MANAUS-AM | SECRETARIA@COIAB.COM.BR
ZapSign 58fb88ab-b3dc-4427-bade-15a92d8b89c6. Documento assinado eletronicamente, conforme MP 2.200-2/2001 e Lei 14.063/2020.

Supplementary Method 7. Formal endorsement by the National Council of Extractive Populations (CNS) for the ethical aspects of primary data collection, article content, and participation in the Evaluation Committee for future research utilizing the Marupiara Dataset.

Conselho Nacional das Populações Extrativistas - CNS

Brasília/DF, 12 de maio de 2025.

Dear Editor and Reviewers of Nature,

The National Council of Extractivist Populations (CNS), which represents traditional extractivist populations in Brazil, has the mission of representing, organizing and guaranteeing the territories of collective use of traditional extractivist populations, articulating, proposing and demanding policies and promoting socio-economic, environmental and cultural sustainability for present and future generations, based on traditional extractivist populations, in accordance with Decree 6.040/2007, which recognizes the rights of traditional peoples and communities in Brazil, with a focus on working with grassroots organizations in the Brazilian Amazon, expresses its support for the publication of the article entitled "Healthy forests safeguard traditional wild meat food systems in Amazonia", which analyses data on hunting practiced by indigenous peoples and traditional communities in Amazonia.

Studies like this, which highlight the importance of sustainable wildlife use and management for our peoples, align fully with our efforts to preserve the traditional relationship we maintain with nature. The findings of this research have the potential to strengthen advocacy at both national and Amazonian scales, underscoring the need to protect our territories to ensure the continuity of our sustainable traditional practices—practices that are vital for our peoples' food security and nutritional well-being.

Furthermore, we commit to collaborating with the article's authors and other representative organizations of Indigenous peoples and traditional communities to establish an Evaluation Committee. This committee will assess potential requests from external researchers for access to the hunting dataset compiled in the study's Database, ensuring any data sharing is conducted ethically and respectfully.

We appreciate the opportunity to contribute to this scientific dialogue and reaffirm our commitment to advancing knowledge that values Indigenous and traditional practices.

Sincerely,

Júlio Barbosa de Aquino
Presidente -CNS

National Council of Extractivist Populations (CNS)

SNQ 111, BLOCO 1, BRASÍLIA/DF
CEP: 70.754-090
E - MAIL: cns.secretarianacional@gmail.com
