# Supplementary material for: Healthy forests safeguard traditional wild meat food systems in Amazonia
Source: Nature. 2025 Nov 26;648(8094):625–33. doi: 10.1038/s41586-025-09743-z (PMC12711560; doi:10.1038/s41586-025-09743-z)
Supplement: Supplementary file 2 — Reporting Summary [file 41586_2025_9743_MOESM2_ESM.pdf]

nature portfolio

Corresponding author(s): André Pinassi Antunes (aapardalis@gmail.com)

Last updated by author(s): Sep 19, 2025

Reporting Summary

Nature Portfolio wishes to improve the reproducibility of the work that we publish. This form provides structure for consistency and transparency in reporting. For further information on Nature Portfolio policies, see our Editorial Policies and the Editorial Policy Checklist.

Statistics

For all statistical analyses, confirm that the following items are present in the figure legend, table legend, main text, or Methods section.

n/a	Confirmed	
☒	☐	The exact sample size (*n*) for each experimental group/condition, given as a discrete number and unit of measurement
☒	☐	A statement on whether measurements were taken from distinct samples or whether the same sample was measured repeatedly
☐	☒	The statistical test(s) used AND whether they are one- or two-sided<br>*Only common tests should be described solely by name; describe more complex techniques in the Methods section.*
☐	☒	A description of all covariates tested
☐	☒	A description of any assumptions or corrections, such as tests of normality and adjustment for multiple comparisons
☐	☒	A full description of the statistical parameters including central tendency (e.g. means) or other basic estimates (e.g. regression coefficient) AND variation (e.g. standard deviation) or associated estimates of uncertainty (e.g. confidence intervals)
☒	☐	For null hypothesis testing, the test statistic (e.g. *F*, *t*, *r*) with confidence intervals, effect sizes, degrees of freedom and *P* value noted<br>*Give P values as exact values whenever suitable.*
☒	☐	For Bayesian analysis, information on the choice of priors and Markov chain Monte Carlo settings
☐	☒	For hierarchical and complex designs, identification of the appropriate level for tests and full reporting of outcomes
☒	☐	Estimates of effect sizes (e.g. Cohen's *d*, Pearson's *r*), indicating how they were calculated

Our web collection on statistics for biologists contains articles on many of the points above.

Software and code

Policy information about availability of computer code

Data collection	no software was used
Data analysis	The analyses were conducted using R software (version 4.1.0), employing several packages for statistical and spatial modelling, including terra (version 1.3-17), sf (version 1.0-8), raster (version 3.4-13), and randomForest (version 4.6-14). Geospatial analyses and map production were performed using QGIS (version 3.22.4), with final editing done in Inkscape (version 1.1) and GIMP (version 2.10.28).

For manuscripts utilizing custom algorithms or software that are central to the research but not yet described in published literature, software must be made available to editors and reviewers. We strongly encourage code deposition in a community repository (e.g. GitHub). See the Nature Portfolio guidelines for submitting code & software for further information.

Data

Policy information about availability of data

All manuscripts must include a data availability statement. This statement should provide the following information, where applicable:

- Accession codes, unique identifiers, or web links for publicly available datasets
- A description of any restrictions on data availability
- For clinical datasets or third party data, please ensure that the statement adheres to our policy

The dataset analyzed during the current study is not publicly available due to sensitivity and privacy concerns related to hunters and their communities. However,

the data can be made available from the corresponding author on reasonable request, contingent on the permission of both the authors and local representatives. Please note, under no circumstances, can the dataset be used in a manner that puts hunters and their communities at risk.

Research involving human participants, their data, or biological material

Policy information about studies with human participants or human data. See also policy information about sex, gender (identity/presentation), and sexual orientation and race, ethnicity and racism.

Reporting on sex and gender	The study does not involve direct research on human participants. Instead, we worked directly with, or utilized published data on, hunting practices by traditional and indigenous communities in Amazonia. Where data was gathered by the authors, hunting communities gave their consent to participate in sharing information on the animal species and numbers of individuals hunted. All communities involved were subjected to clear agreements, including Free, Prior, and Informed Consent (FPIC) procedures, ensuring ethical engagement and respect for their rights and autonomy. No specific information on gender or sex in these communities was necessary to be considered in our study, as the inclusive nature of the hunting practices, where wild meat is shared among families and communities, ensures that the data represents all members of the community.
Reporting on race, ethnicity, or other socially relevant groupings	Although each community where hunting data was collected (from both primary and secondary sources) was characterised according to its ethnic group, this information was not used in the primary analyses. However, we recognize that distinct ethnic groups may hunt different species due to cultural aspects and preferences. To investigate how different ethnic groups influence hunting patterns in Amazonia, we used a raster of the Amazonian language families as a covariate. This raster was constructed from maps provided by Loukotka (1967) and Eriksen (2011).
Population characteristics	The study focuses on wildlife hunting practices and does not involve collecting demographic data on human populations.
Recruitment	Most communities from which our hunting data were extracted were actively and formally involved in hunting monitoring schemes, some of which have been active for decades. Each of these initiatives followed an explicit process to encourage hunters or monitors to participate voluntarily. The selection procedures, along with details of the communities and individuals involved, are documented elsewhere. These reports provide comprehensive insights into the engagement processes and methodologies employed, ensuring accurate and reliable data collection.
Ethics oversight	This study created and analyzed the most comprehensive dataset to date on hunting and wild meat extraction in Amazonia, without involving human participants or human data. As a result, ethical approval specific to human subjects was not required.

Note that full information on the approval of the study protocol must also be provided in the manuscript.

Field-specific reporting

Please select the one below that is the best fit for your research. If you are not sure, read the appropriate sections before making your selection.

☐ Life sciences ☐ Behavioural & social sciences ☒ Ecological, evolutionary & environmental sciences

For a reference copy of the document with all sections, see nature.com/documents/nr-reporting-summary-flat.pdf

Ecological, evolutionary & environmental sciences study design

All studies must disclose on these points even when the disclosure is negative.

Study description	This study analyses the hunting practices of Amazonian peoples—including rural Indigenous, traditional, and farming communities—over nearly six decades, with a focus on the composition of hunted taxa and hunter harvest rates. Utilizing both primary and secondary data on wild terrestrial vertebrates, the study derives key metrics such as Taxon-Specific Offtake Proportion (TSOP) and Hunter Harvest Rate (HHR). Additionally, random forest models are employed to spatially predict these metrics in relation to environmental and anthropogenic variables.
Research sample	The research sample includes data on 447,438 individual animal kills recorded across 647 rural localities in Amazonia. The communities involved in this data collection consist of Indigenous, traditional, and farming peoples residing in rural areas of the region. These localities were selected due to the presence of existing hunting monitoring projects. Data sources encompass primary data from long-term monitoring efforts and shorter studies conducted by local monitors, as well as secondary data from published literature. This information details species, the number of animals, biomass hunted, and hunting effort.
Sampling strategy	The sampling strategy included both long-term monitoring initiatives and shorter studies conducted by the authors, supplemented by secondary data extracted from the literature. The selection of communities, hunters, or monitors for primary data collection was based on the specific objectives of each monitoring initiative, rather than being tailored specifically for this study.
Data collection	Secondary data were obtained from published sources, ensuring comprehensive coverage across different regions of Amazonia.
Timing and spatial scale	The study encompasses data collected from 1965 to 2023, covering a range of temporal scales. Spatially, it includes 647 localities across the entire Amazon basin, offering a comprehensive view of hunting practices on a large scale.

Data exclusions	Data on wild meat trade and urban consumption were excluded to maintain a focus solely on hunting practices within rural Amazonian communities. Additionally, any records with missing or unclear information were omitted from the analysis.
Reproducibility	Measures to ensure reproducibility included the use of established methodologies for data collection and analysis, such as standardized monitoring protocols and widely recognized statistical and spatial analysis tools. Detailed descriptions of data sources, sampling strategies, and analytical procedures are provided in the Methods section to facilitate replication. Furthermore, the R scripts used for the analysis are available from the corresponding author upon reasonable request.
Randomization	Randomization was not applicable in this observational study, as the data reflect natural hunting practices recorded over time. All possible available data were included to ensure comprehensive coverage and representation.
Blinding	Blinding was not applicable as this study involved the analysis of preexisting data on wildlife hunting. The data were collected through observational monitoring of natural hunting practices by local communities, where blinding is not feasible or relevant.

Did the study involve field work? ☒ Yes ☐ No

Field work, collection and transport

Field conditions	Field data collection at the various sites reported in our research was conducted under a range of environmental conditions typical of the Amazon region, including dense forests, rivers, and floodplains.
Location	Data were collected from 647 rural localities across the Amazon basin, representing a variety of ecological zones. More detailed information about these localities can be found in the Methods section.
Access & import/export	Access to field sites was coordinated with local communities and authorities. Since the study focused on data collection within the Amazon region, there was no need for import or export of materials.
Disturbance	In all reported study sites, minimal disturbance to wildlife and habitats was prioritized, with data collection methods designed to be non-intrusive and respectful of local customs and regulations. All research was conducted in areas where a prior trust relationship had been established with local communities.

Reporting for specific materials, systems and methods

We require information from authors about some types of materials, experimental systems and methods used in many studies. Here, indicate whether each material, system or method listed is relevant to your study. If you are not sure if a list item applies to your research, read the appropriate section before selecting a response.

Materials & experimental systems

n/a	Involved in the study	
☒	☐	Antibodies
☒	☐	Eukaryotic cell lines
☒	☐	Palaeontology and archaeology
☒	☐	Animals and other organisms
☒	☐	Clinical data
☒	☐	Dual use research of concern
☒	☐	Plants

Methods

n/a	Involved in the study	
☒	☐	ChIP-seq
☒	☐	Flow cytometry
☒	☐	MRI-based neuroimaging

Plants

Seed stocks	N/A
Novel plant genotypes	N/A
Authentication	N/A
